# 1,2,3-Benzoxathiazine-2,2-dioxides – effective inhibitors of human carbonic anhydrases

**DOI:** 10.1080/14756366.2022.2142787

**Published:** 2022-11-13

**Authors:** Jekaterina Ivanova, Morteza Abdoli, Alessio Nocentini, Raivis Žalubovskis, Claudiu T. Supuran

**Affiliations:** a Latvian Institute of Organic Synthesis, Riga, Latvia; b Faculty of Materials Science and Applied Chemistry, Institute of Technology of Organic Chemistry, Riga Technical University, Riga, Latvia; c Neurofarba Department, Università degli Studi di Firenze, Florence, Italy

**Keywords:** Carbonic anhydrase, 123-benzoxathiazine 22-dioxide, inhibitors

## Abstract

A series of 1,2,3-benzoxathiazine-2,2-dioxides possessing various substituents in the 5, 7, or 8 position was obtained from corresponding 2-hydroxybenzaldehydes in their reaction with sulfamoyl chloride. 5-, 7-, and 8-aryl substituted 1,2,3-benzoxathiazine-2,2-dioxides were prepared from aryl substituted 2-hydroxybenzaldehydes obtained from 3-, 4-, or 6-bromo-2-hydroxybenzaldehydes *via* two-step protocol. 1,2,3-Benzoxathiazine-2,2-dioxides were investigated for the inhibition of four human carbonic anhydrase (hCA, EC 4.2.1.1) isoforms, cytosolic hCA I and II and tumour-associated transmembrane hCA IX and XII. Twenty four derivatives of 1,2,3-benzoxathiazine 2,2-dioxide were obtained. Most of them act as nanomolar inhibitors of hCA IX and XII. Almost all compounds except **2d** and **5a-e** also express nanomolar inhibitory activity for hCA II. hCA I is poorly inhibited or not inhibited by 1,2,3-benzoxathiazine 2,2-dioxides. Some of the new derivatives exhibit promising selectivity towards CA IX/XII over hCA I, although none of the compounds are selective towards CA IX/XII over both hCA I and II.

## Introduction

Human carbonic anhydrases (hCAs, EC 4.2.1.1) are a superfamily of widespread metalloenzymes in most living organisms that catalyse a reversible hydration of carbon dioxide (CO_2_) into bicarbonate ion (HCO_3_^–^) and proton (H^+^)^1–5^.

CAs are involved in such important physiological processes as respiration, metabolism, pH regulation, ion transport, bone resorption, secretion of gastric, cerebrospinal fluid, and pancreatic juices, biosynthetic reactions such as gluconeogenesis and ureagenesis, etc^1–3^.

Sixteen different CA isoforms have been identified and characterised in mammals so far^1^^,^^3^.

Human CAs are considered as promising therapeutic targets for a wide range of diseases, such as epilepsy, cerebral and retinal oedema, glaucoma (hCA I and hCA II), haemolytic anaemia, obesity, sterility, and cancer^1^^,^^6^.

Most efforts in the last few decades have been focussed on the tumour-associated isoforms (hCA IX and XII) that were shown to possess an important role in hypoxic tumour physiopathology and thus validated as biomarkers and therapeutic targets for various cancer types. Their inhibition has been related to the reduction of primary tumour growth, inhibition of invasion and metastasis, and a reduction in the cancer stem cell population^7–9^.

hCA IX expression is associated with a bad prognosis in cancer, whereas hCA XII is expressed in normal tissues and overexpressed in a number of malignant tumors^4^^,^^10^.

hCA IX has been considered as a valuable marker for cancer, and the development of hCA IX inhibitors with selectivity over widely distributed isoforms hCA I/II is a potential strategy for designing anticancer agents^3^.

During the last decade, a wide range of novel CA inhibitors has been discovered, such as saccharin derivatives^11–13^, thiophene moiety containing sulfonamides^14^, furagin derivatives^15^, and many more.

Sulphonamide moiety is the most widespread zinc-binding group (ZBG) of CA inhibitors. Non-classical CA inhibitors such as coumarins, carboxylic acids, phenols, polyamines inhibit the catalytic activity of CA by different mechanisms rather than coordinating to the zinc^10^.

Coumarin ring showed exceptional anticancer properties acting through various mechanisms of action^10^^,^^16^^,^^17^. Coumarin derivatives were introduced by Supuran’s group as a non-classical type of CAIs^18^.

The mechanism of action of coumarin as CA inhibitor is based on hydrolysis forming cis-2-hydroxy-cinnamic acid, instead of binding the CA active site with its intact coumarin moiety^10^.

The ability of coumarin to inhibit CA triggered the investigation of coumarin derivatives and its bioisosteres as CA inhibitors.^19^

In 2013, coumarin bioisosteres 1,2-benzoxathiine 2,2-dioxides, also referred as sulfocoumarins, were reported as a new class of prodrug-type CA inhibitors, some of them showing low nanomolar inhibitory activities.^20^ It was demonstrated that sulfocoumarins (1,2-benzoxathiine 2,2-dioxides) possess a similar mechanism of action as coumarins, acting as effective CA inhibitors. The sulfocoumarins were hydrolysed by the esterase CA activity to 2-hydroxyphenyl-vinylsulfonic acids, which then bind to the enzyme in a region rarely occupied by other classes of inhibitors.^20^ Later a wide range of sulfocoumarin derivatives has been investigated and tested, showing significant isoform-selective CA inhibition properties^21–26^.

In 2017, homologs of sulfocoumarin-3*H*-1,2-benzoxathiepine 2,2-dioxides, i.e. homo-sulfocoumarins, were introduced as CA inhibitors for the first time. As the results of the biological screening show, homo-sulfocoumarins possess very high level of CA isoform selectivity as well^27–30^.

**Figure 1.**
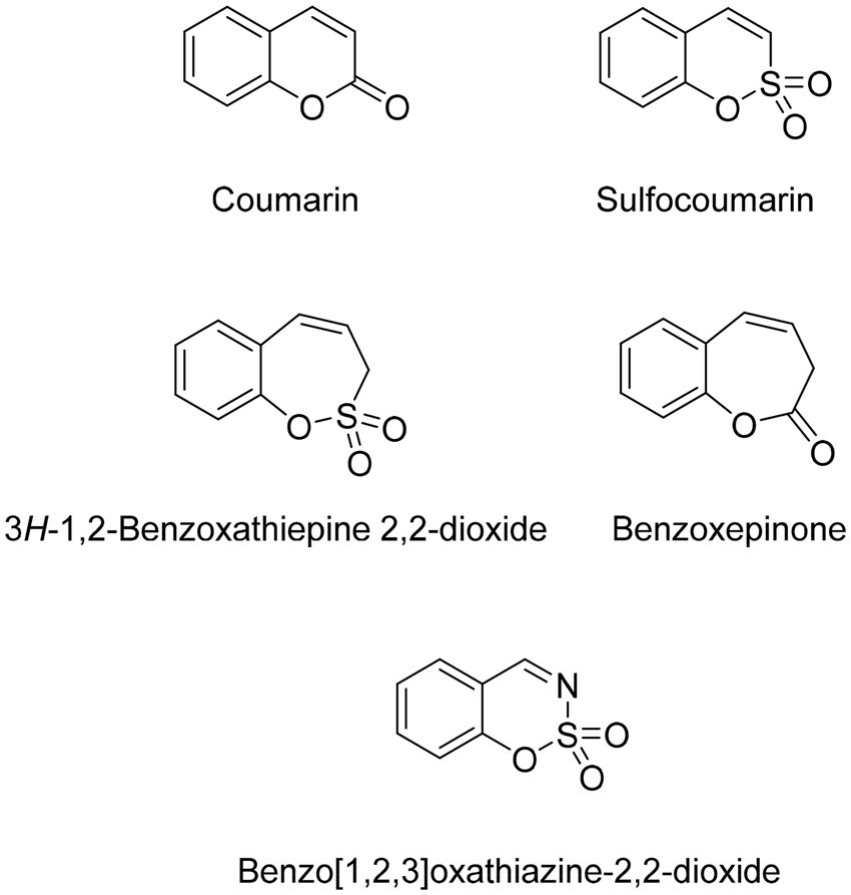
Structure of CA inhibitors.

In 2020, one more novel isoform-selective class of compounds – the bioisosteres of homo-sulfocoumarins, namely benzoxepinones (Figure 1), were reported as CA inhibitors for the first time.^31^

Taking into account that coumarin bioisosteres and their homologs described above show excellent CA inhibitory properties along with significant selectivity for the inhibition of hCA IX and hCA XII over hCA I and hCA II, we report here a series of nitrogen-containing analogues of 1,2-benzoxathiine 2,2-dioxides, namely derivatives of 1,2,3-benzoxathiazine 2,2-dioxide.

## Materials and methods

### Chemistry

Reagents, starting materials, and solvents were obtained from commercial sources and used as received. Thin-layer chromatography was performed on silica gel, spots were visualised with UV light (254 and 365 nm). Melting points were determined on an OptiMelt automated melting point system. IR spectra were recorded on Shimadzu FTIR IR Prestige-21 spectrometer. NMR spectra were recorded on Bruker Avance Neo (400 MHz) spectrometer with chemical shifts values (δ) in ppm relative to TMS using the residual DMSO-d_6_ signal (^1^H 2.50; ^13^C 39.52) or CDCl_3_ signal (^1^H 7.26; ^13^C 77.16) as an internal standard. High-resolution mass spectra (HRMS) were recorded on a mass spectrometer with a Q-TOF micro mass analyser using the ESI technique. Elemental analyses were performed on Carlo Erba CHNS-O EA-1108 apparatus. GC-MS analyses were performed on *Agilent Technologies* 7890 A gas chromatograph, column – *HP-5HS* (df = 0.25 μm, ID = 0.25 mm, length − 30 m) with *Agilent Technologies* 5975C masselective detector.

### Synthesis

#### Sulfamoyl chloride


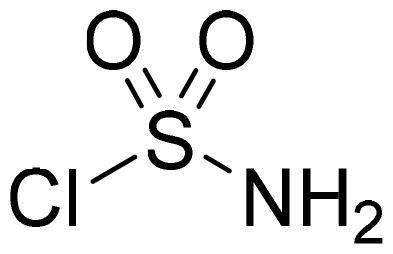


Chlorosulfonylisocyanate (3.80 ml, 43.3 mmol) was cooled to +5 °C. Formic acid (1.67 ml, 43.3 mmol) was added dropwise in temperature range between +5 - +13 °C. Reaction mixture was stirred at 0 °C for additional 45 min, and then allowed to warm to room temperature. Toluene (30 ml) was added to the reaction mixture, precipitate was filtered, filtrate was evaporated. Obtained as yellow oily solid (6.58 g, 98%). Mp 36–37 °C.

#### General procedure for the preparation of 1,2,3-benzoxathiazine 2,2-dioxides 2a–k

The derivative of 2-hydroxybenzaldehyde (1 equiv) was dissolved in dry DMA (6 ml). Reaction mixture was cooled to 0 °C. Sulfamoyl chloride (2.5 equiv) was slowly added to the reaction mixture. The stirring was continued at room temperature under argon atmosphere for 24–72 h. Reaction mixture was then poured into ice-water (25 ml), extracted with DCM (3 × 25 ml), washed with satd. NaHCO_3_ (3 × 25 ml) and brine (3 × 25 ml). Organic phase was dried over Na_2_SO_4_, filtered, evaporated. The product was purified by column chromatography on silica gel with PE/EtOAc (2:1) followed by recrystallisation from EtOH.

#### 6-Methyl-1,2,3-benzoxathiazine 2,2-dioxide (2a)


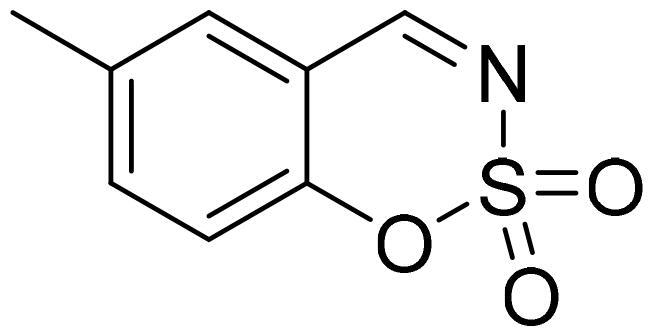


Compound **2a** was obtained from 2-hydroxy-4-methylbenzaldehyde (0.20 g, 1.47 mmol) and sulfamoyl chloride (0.42 g, 3.68 mmol). Reaction mixture was stirred for 48 h. Obtained as white solid (0.19 g, 64%). Mp 94–95 °C.

^1^H NMR (400 MHz, CDCl_3_): δ 8.61 (1H, s), 7.55 (1H, dd, *J*= 0.7 Hz, *J*= 2.2 Hz), 7.46 (1H, dd, *J*= 0.4 Hz, *J*= 1.6 Hz), 7.19 (1H, d, *J*= 8.5 Hz), 2.44 (3H, s) ppm.

^13^C NMR (100 MHz, CDCl_3_): δ 167.9, 152.4, 138.6, 136.5, 130.7, 118.5, 115.3, 20.8 ppm.

Anal. Calcd. for C_8_H_7_NO_3_S: C, 48.72; H, 3.58; N 7.10. Found: C, 48.24; H, 3.57; N, 7.00.

GC-MS (m/z, %): 51 (21), 77 (27), 78 (53), 104 (40), 106 (20), 132 (87), 197 (100).

IR (KBr, cm^−1^), ν_max_ = 1382 (S = O), 1185 (S = O).

#### 6-Methoxy-1,2,3-benzoxathiazine 2,2-dioxide (2b)


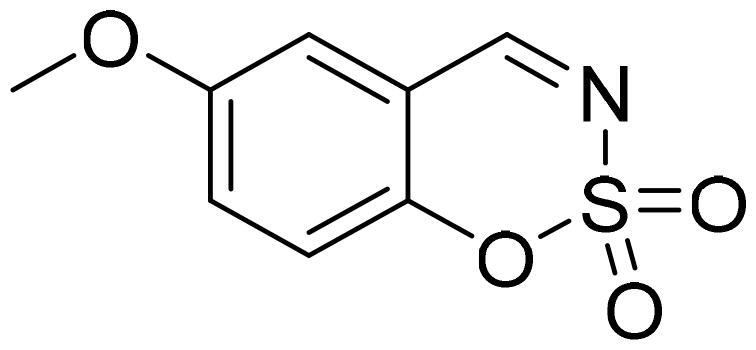


Compound **2b** was obtained from 2-hydroxy-5-methoxybenzaldehyde (0.16 ml, 1.32 mmol) and sulfamoyl chloride (0.38 g, 3.30 mmol). Reaction mixture was stirred for 72 h. Purified by column chromatography with PE/EtOAc (1:1). Obtained as light yellow solid (0.10 g, 36%). Mp 121–122 °C.

^1^H NMR (400 MHz, CDCl_3_): δ 8.67 (1H, s), 7.33 (1H, dd, *J*= 2.9 Hz, *J*= 9.1 Hz), 7.29 (1H, d, *J*= 8.5 Hz), 7.14 (1H, d, *J*= 2.9 Hz), 3.92 (3H, s) ppm.

^13^C NMR (100 MHz, CDCl_3_): δ 167.7, 157.3, 148.2, 124.6, 119.9, 115.8, 113.2, 56.3 ppm.

Anal. Calcd. for C_8_H_7_NO_4_S: C, 45.07; H, 3.31; N 6.57. Found: C, 45.04; H, 3.32; N, 6.50.

GC-MS (m/z, %): 79 (39), 107 (26), 134 (100), 149 (40), 213 (51).

IR (KBr, cm^−1^), ν_max_ = 1375 (S = O), 1362 (S = O), 1183 (S = O), 1168 (S = O).

#### 6-Fluoro-1,2,3-benzoxathiazine 2,2-dioxide (2c)


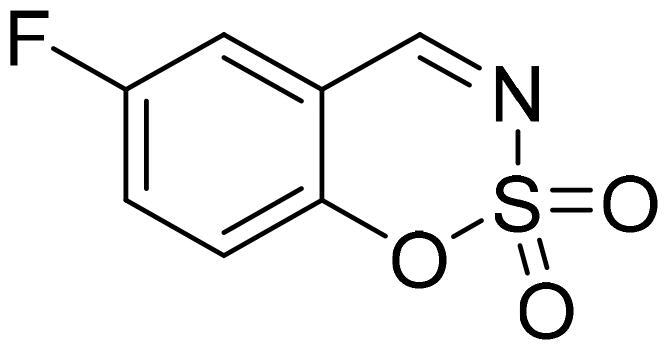


Compound **2c** was obtained from 5-fluoro-2-hydroxybenzaldehyde (0.20 g, 1.43 mmol) and sulfamoyl chloride (0.41 g, 3.58 mmol). Reaction mixture was stirred for 72 h. Obtained as white solid (0.14 g, 48%). Mp 142–143 °C.

^1^H NMR (400 MHz, CDCl_3_): δ 8.64 (1H, s), 7.48 (1H, ddd, *J*= 3.0 Hz, *J*= 7.6 Hz, *J*= 9.1 Hz), 7.39 (1H, dd, *J*= 3.3 Hz, *J*= 6.9 Hz), 7.32 (1H, dd, *J*= 4.1 Hz, *J*= 9.1 Hz) ppm.

^13^C NMR (100 MHz, CDCl_3_): δ 166.7 (d, *J*= 2.3 Hz), 159.3 (d, *J*= 249 Hz), 150.4 (d, *J*= 2.5 Hz), 125.0 (d, *J*= 24.0 Hz), 120.8 (d, *J*= 7.9 Hz), 116.5 (d, *J*= 24.5 Hz), 116.0 (d, *J*= 8.1 Hz) ppm.

Anal. Calcd. for C_7_H_4_FNO_3_S: C, 41.79; H, 2.00; N 6.96. Found: C, 41.51; H, 2.18; N, 6.70.

GC-MS (m/z, %): 81 (31), 109 (92), 137 (54), 201 (100).

IR (KBr, cm^−1^), ν_max_ = 1388 (S = O), 1353 (S = O), 1189 (S = O), 1154 (S = O).

#### 6-Bromo-1,2,3-benzoxathiazine 2,2-dioxide (2d)


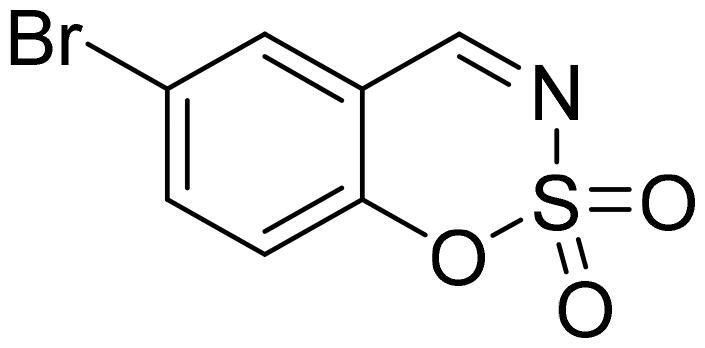


Compund **2d** was obtained from 2-hydroxy-6-bromobenzaldehyde (0.20 g, 1.00 mmol) and sulfamoyl chloride (0.29 g, 2.50 mmol). Reaction mixture was stirred for 48 h. Obtained as white solid (0.12 g, 47%). Mp 154–155 °C.

^1^H NMR (400 MHz, CDCl_3_): δ 8.62 (1H, s), 7.85 (1H, dd, *J*= 2.2 Hz, *J*= 8.7 Hz), 7.82 (1H, d, *J*= 2.2 Hz), 7.21 (1H, d, *J*= 8.7 Hz) ppm.

^13^C NMR (100 MHz, CDCl_3_): δ 166.4, 153.3, 140.4, 133.1, 120.6, 118.9, 116.6 ppm.

Anal. Calcd. for C_7_H_4_BrNO_3_S: C, 32.08; H, 1.54; N 5.34. Found: C, 32.08; H, 1.40; N, 5.20.

GC-MS (m/z, %): 62 (30), 63 (100), 90 (27), 171 (56), 197 (53), 263 (83).

IR (KBr, cm^−1^), ν_max_ = 1381 (S = O), 1345 (S = O), 1179 (S = O).

#### 7-Methyl-1,2,3-benzoxathiazine 2,2-dioxide (2e)


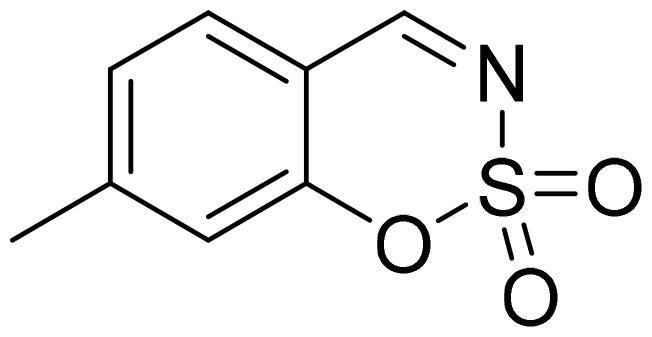


Compound **2e** was obtained from 2-hydroxy-4-methylbenzaldehyde (0.20 g, 1.47 mmol) and sulfamoyl chloride (0.42 g, 3.68 mmol). Reaction mixture was stirred for 72 h. Purified by column chromatography with PE/EtOAc (1:1). Obtained as light yellow solid (0.20 g, 70%). Mp 79–80 °C.

^1^H NMR (400 MHz, CDCl_3_): δ 8.60 (1H, s), 7.55 (1H, d, *J*= 7.9 Hz), 7.20–7.24 (1H, m), 7.10 (1H, s), 2.50 (3H, s) ppm.

^13^C NMR (100 MHz, CDCl_3_): δ 167.6, 154.5, 150.5, 130.7, 127.3, 118.9, 113.3, 22.5 ppm.

Anal. Calcd. for C_8_H_7_NO_3_S: C, 48.72; H, 3.58; N 7.10. Found: C, 48.70; H, 3.67; N, 7.05.

GC-MS (m/z, %): 77 (22), 78 (49), 104 (50), 132 (55), 197 (100).

IR (KBr, cm^−1^), ν_max_ = 1381 (S = O), 1195 (S = O).

#### 7-Methoxy-1,2,3-benzoxathiazine 2,2-dioxide (2f)


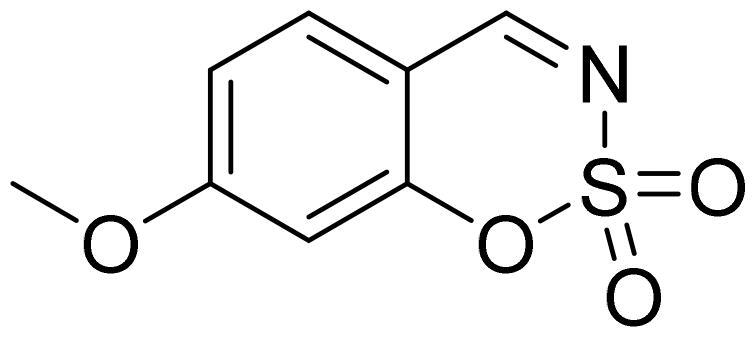


Compound **2f** was obtained from 2-hydroxy-4-methoxybenzaldehyde (0.20 g, 1.34 mmol) and sulfamoyl chloride (0.39 g, 3.35 mmol). Reaction mixture was stirred for 48 h. Obtained as light brown solid (0.19 g, 67%). Mp 125–126 °C.

^1^H NMR (400 MHz, CDCl_3_): δ 8.51 (1H, s), 7.56 (1H, d, *J*= 8.7 Hz), 6.90 (1H, dd, *J*= 2.4 Hz, *J*= 8.7 Hz), 6.73 (1H, d, *J*= 2.4 Hz), 3.94 (3H, s) ppm.

^13^C NMR (100 MHz, CDCl_3_): δ 167.4, 166.9, 157.0, 132.7, 113.7, 109.3, 103.0, 56.6 ppm.

Anal. Calcd. for C_8_H_7_NO_4_S: C, 45.07; H, 3.31; N 6.57. Found: C, 45.15; H, 3.27; N, 6.51.

GC-MS (m/z, %): 106 (44), 134 (49), 149 (18), 213 (100).

IR (KBr, cm^−1^), ν_max_ = 1383 (S = O), 1357 (S = O), 1184 (S = O), 1156 (S = O).

#### 7-Fluoro-1,2,3-benzoxathiazine 2,2-dioxide (2g)


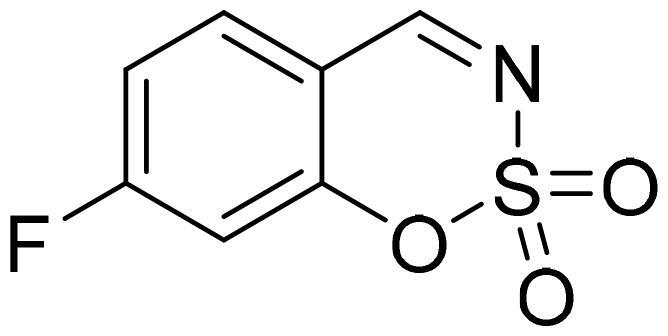


Compund **2g** was obtained from 2-hydroxy-4-fluorobenzaldehyde (0.20 g, 1.43 mmol) and sulfamoyl chloride (0.41 g, 3.58 mmol). Reaction mixture was stirred for 72 h. Purified by column chromatography with PE/EtOAc (1:1). Obtained as light brown solid (0.15 g, 51%). Mp 67–68 °C.

^1^H NMR (400 MHz, CDCl_3_): δ 8.62 (1H, s), 7.72 (1H, dd, *J*= 5.8 Hz, *J*= 8.5 Hz), 7.14 (1H, ddd, *J*= 2.4 Hz, *J*= 7.9 Hz, *J*= 8.5 Hz), 7.03 (1H, dd, *J*= 2.4 Hz, *J*= 8.5 Hz) ppm.

^13^C NMR (100 MHz, CDCl_3_): δ 167.7 (d, *J*= 263.8 Hz), 166.8, 156.3 (d, *J*= 13.7 Hz), 133.4 (d, *J*= 11.4 Hz), 114.5 (d, *J*= 22.9 Hz), 112.4 (d, *J*= 3.1 Hz), 107.0 (d, *J*= 26.1 Hz) ppm.

Anal. Calcd. for C_7_H_4_FNO_3_S: C, 41.79; H, 2.00; N 6.96. Found: C, 41.75; H, 2.00; N, 6.90.

GC-MS (m/z, %): 81 (21), 82 (49), 109 (74), 110 (26), 137 (37), 201 (100).

IR (KBr, cm^−1^), ν_max_ = 1395 (S = O), 1385 (S = O), 1142 (S = O).

#### 7-Bromo-1,2,3-benzoxathiazine 2,2-dioxide (2h)


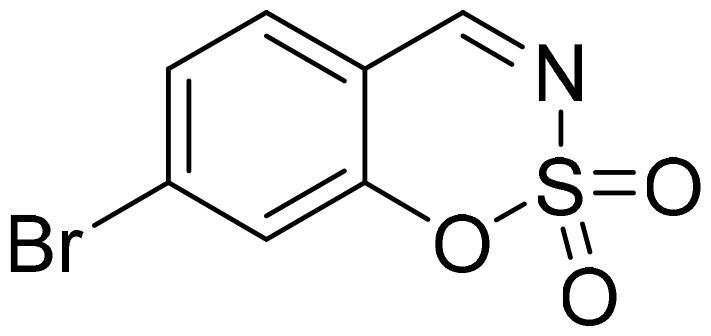


Compound **2h** was obtained from 4-bromo-2-hydroxybenzaldehyde (0.20 g 1.00 mmol) and sulfamoyl chloride (0.29 g, 2.50 mmol). Reaction mixture was stirred for 72 h. Obtained as white solid (0.14 g, 55%). Mp 120–121 °C.

^1^H NMR (400 MHz, CDCl_3_): δ 8.63 (1H, s), 7.58 (1H, dd, *J*= 1.6 Hz, *J*= 8.2 Hz), 7.54 (1H, d, *J*= 8.2 Hz), 7.50 (1H, d, *J*= 1.6 Hz) ppm.

^13^C NMR (100 MHz, CDCl_3_): δ 167.0, 154.5, 132.6, 131.6, 129.9, 122.3, 114.2 ppm.

Anal. Calcd. for C_7_H_4_BrNO_3_S: C, 32.08; H, 1.54; N 5.34. Found: C, 32.28; H, 1.57; N, 5.17.

GC-MS (m/z, %): 62 (27), 63 (83), 90 (42), 169 (60), 197 (34), 263 (100).

IR (KBr, cm^−1^), ν_max_ = 1384 (S = O).

#### 8-Methoxy-1,2,3-benzoxathiazine 2,2-dioxide (2i)


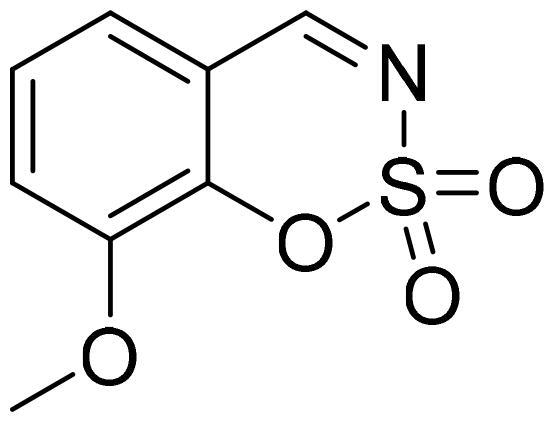


Compound **2i** was obtained from 2-hydroxy-3-methoxybenzaldehyde (0.20 g, 1.34 mmol) and sulfamoyl chloride (0.39 g, 3.35 mmol). Reaction mixture was stirred for 24 h. Obtained as white solid (0.09 g, 30%). Mp 140–141 °C.

^1^H NMR (400 MHz, CDCl_3_): δ 8.57 (1H, s), 7.22–7.31 (2H, m), 7.15–7.20 (1H, m), 3.90 (3H, s) ppm.

^13^C NMR (100 MHz, CDCl_3_): δ 168.1, 148.5, 143.7, 126.2, 121.6, 120.0, 116.1, 56.8 ppm.

Anal. Calcd. for C_8_H_7_NO_4_S: C, 45.07; H, 3.31; N 6.57. Found: C, 44.97; H, 3.26; N, 6.42.

GC-MS (m/z, %): 51 (32), 106 (37), 107 (57), 119 (22), 134 (38), 149 (35), 213 (100).

IR (KBr, cm^−1^), ν_max_ = 1383 (S = O), 1359 (S = O), 1185 (S = O), 1171 (S = O).

#### 8-Fluoro-1,2,3-benzoxathiazine 2,2-dioxide (2j)


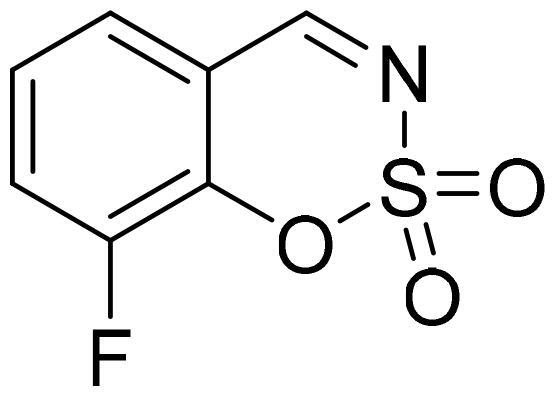


Compound **2j** was obtained from 2-hydroxy-3-fluorobenzaldehyde (0.20 g, 1.43 mmol) and sulfamoyl chloride (0.41 g, 3.58 mmol). Reaction mixture was stirred for 72 h. Purified by column chromatography with PE/EtOAc (1:1). Obtained as white solid (0.12 g, 42%). Mp 83–84 °C.

^1^H NMR (400 MHz, CDCl_3_): δ 8.69 (1H, d, *J*= 1.2 Hz), 7.53–7.59 (1H, m), 7.47–7.51 (1H, m), 7.39 (1H, dq, *J*= 4.3 Hz, *J*= 7.9 Hz) ppm.

^13^C NMR (100 MHz, CDCl_3_): δ 167.3 (d, *J*= 3.0 Hz), 150.3 (d, *J*= 256.5 Hz), 142.5 (d, *J*= 13.7 Hz), 126.4 (d, *J*= 6.6 Hz), 125.9 (d, *J*= 3.8 Hz), 124.6 (d, *J*= 17.6 Hz), 117.0 ppm.

Anal. Calcd. for C_7_H_4_FNO_3_S: C, 41.79; H, 2.00; N 6.96. Found: C, 41.80; H, 1.98; N, 6.73.

GC-MS (m/z, %): 81 (30), 82 (59), 109 (74), 137 (63), 201 (100).

IR (KBr, cm^−1^), ν_max_ = 1399 (S = O), 1363 (S = O), 1191 (S = O).

#### 8-Bromo-1,2,3-benzoxathiazine 2,2-dioxide (2k)


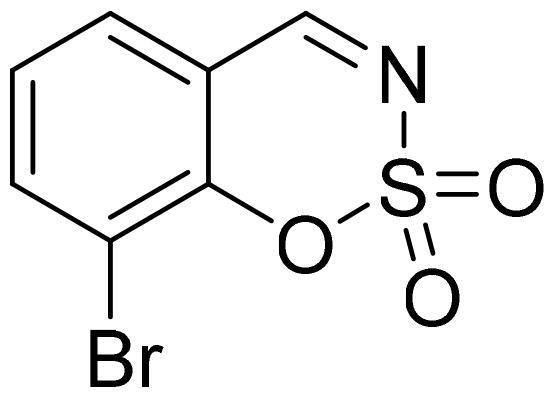


Compound **2k** was obtained from 3-bromo-2-hydroxybenzaldehyde (0.20 g, 1.00 mmol) and sulfamoyl chloride (0.29 g, 2.50 mmol). Reaction mixture was stirred for 72 h. Purified by column chromatography with PE/EtOAc (1:1). Obtained as white solid (0.10 g, 39%). Mp 130–131 °C.

^1^H NMR (400 MHz, CDCl_3_): δ 8.63 (1H, s), 7.96 (1H, dd, *J*= 1.5 Hz, *J*= 8.1 Hz), 7.65 (1H, dd, *J*= 1.5 Hz, *J*= 7.7 Hz), 7.33 (1H, t, *J*= 7.8 Hz) ppm.

^13^C NMR (100 MHz, CDCl_3_): δ 167.3, 151.5, 141.0, 129.9, 126.9, 116.7, 112.6 ppm.

Anal. Calcd. for C_7_H_4_BrNO_3_S: C, 32.08; H, 1.54; N 5.34. Found: C, 32.22; H, 1.58; N, 5.30.

GC-MS (m/z, %): 62 (33), 63 (100), 64 (27), 90 (54), 118 (57), 169 (36), 263 (99).

IR (KBr, cm^−1^), ν_max_ = 1378 (S = O), 1355 (S = O), 1180 (S = O).

#### General procedure for the preparation of 2-hydroxybenzaldehydes 4a-m

The derivative of 2-hydroxybenzaldehyde (1 equiv), the derivative of benzeneboronic acid (1.3 equiv), K_2_CO_3_ (2.5 equiv), and Pd(PPh_3_)_4_ (0.05 equiv) were suspended in toluene/water (5:1, 20 ml) mixture in pressure tube. Reaction mixture was heated to 90 °C and stirred for 24 h, then cooled to room temperature, filtered through celite. EtOAc (30 ml) was added, reaction mixture was washed with satd. NaHCO_3_ (3× 25 ml) and brine (2× 25 ml). Organic phase was dried over Na_2_SO_4_, filtered, evaporated. The product was purified by column chromatography with PE/EtOAc (10:1) followed by recrystallisation from EtOH.

#### 3-Hydroxy-1,1'-biphenyl-2-carbaldehyde (4a)


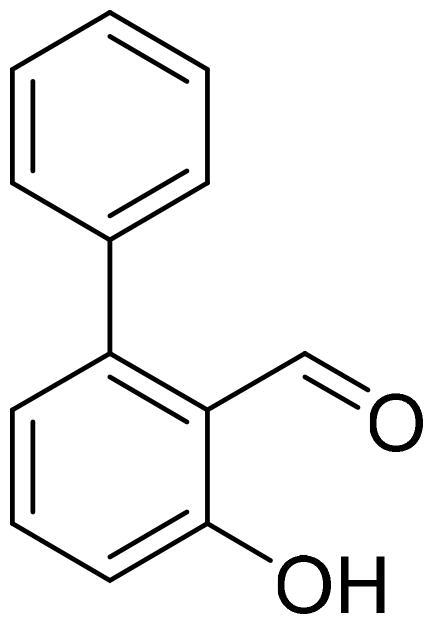


Compound **4a** was obtained from 6-bromo-2-hydroxybenzaldehyde (0.50 g, 2.49 mmol), benzeneboronic acid (0.39 g, 3.23 mmol), K_2_CO_3_ (0.86 g, 6.22 mmol), and Pd(PPh_3_)_4_ (0.14 g, 0.12 mmol). Reaction mixture was stirred for 48 h, the product was purified by column chromatography with PE/EtOAc (2:1) followed by EtOAc (100%). Obtained as yellow solid (0.33 g, 67%). Mp 32–33 °C.

^1^H NMR (400 MHz, CDCl_3_): δ 11.92 (1H, s), 9.84 (1H, d, *J*= 0.6 Hz), 7.50–7.60 (1H, m), 7.43–7.49 (3H, m), 7.35–7.40 (2H, m), 6.98–7.02 (1H, m), 6.89 (1H, dd, *J*= 1.1 Hz, *J*= 7.5 Hz) ppm.

^13^C NMR (100 MHz, CDCl_3_): δ 197.3, 162.9, 147.6, 137.6, 136.7, 130.2, 128.5, 128.4, 121.6, 118.1, 117.1 ppm.

HRMS (ESI, m/z): calcd for C_13_H_9_O_2_ [M-H]^-^ 197.0603, found 197.0606.

GC-MS (m/z, %): 115 (25), 141 (22), 152 (20), 197 (100).

IR (KBr, cm^−1^), ν_max_ = 3058 (OH), 1656 (C = O).

#### 4'-Fluoro-3-hydroxy-1,1'-biphenyl-2-carbaldehyde (4b)


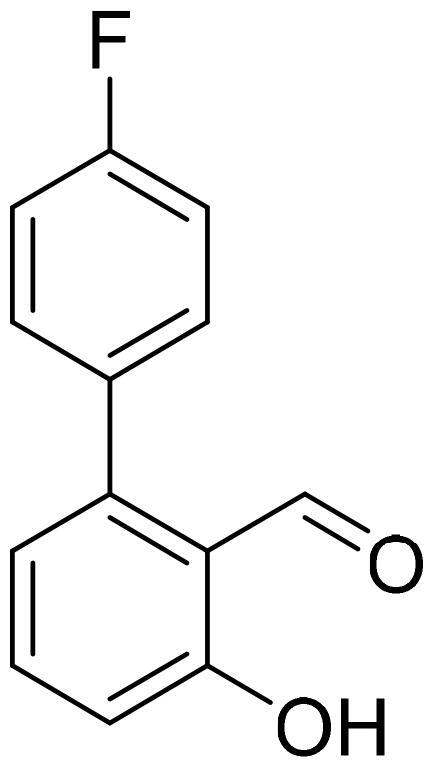


Compound **4b** was obtained from 6-bromo-2-hydroxybenzaldehyde (0.50 g, 2.49 mmol), 4-fluorobenzeneboronic acid (0.45 g, 3.23 mmol), K_2_CO_3_ (0.86 g, 6.22 mmol), and Pd(PPh_3_)_4_ (0.14 g, 0.12 mmol). Obtained as white solid (0.49 g, 91%). Mp 72–73 °C.

^1^H NMR (400 MHz, CDCl_3_): δ 11.89 (1H, s), 9.82 (1H, d, *J*= 0.6 Hz), 7.53 (1H, dd, J= 7.7 Hz, *J*= 8.5 Hz), 7.31–7.38 (2H, m), 7.16 (2H, t, *J*= 8.6 Hz), 6.99–7.02 (1H, m), 6.86 (1H, dd, *J*= 1.1 Hz, *J*= 7.5 Hz) ppm.

^13^C NMR (100 MHz, CDCl_3_): δ 197.0, 163.1, 163.0 (d, *J*= 248.9 Hz), 146.5, 136.9, 133.7 (d, *J*= 3.5 Hz), 131.9 (d, *J*= 8.3 Hz), 121.8, 118.3, 117.4, 115.8 (d, *J*= 21.5 Hz) ppm.

HRMS (ESI, m/z): calcd for C_13_H_8_O_2_F [M-H]^-^ 215.0508, found 215.0510.

GC-MS (m/z, %): 120 (22), 133 (24), 159 (20), 170 (25), 215 (78), 216 (100).

IR (KBr, cm^−1^), ν_max_ = 3044 (OH), 1653 (C = O).

#### 4'-Methoxy-3-hydroxy-1,1'-biphenyl-2-carbaldehyde (4c)


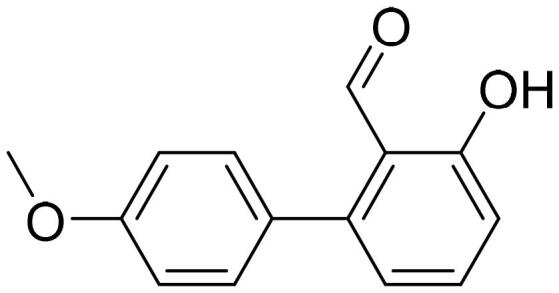


Compound **4c** was obtained from 6-bromo-2-hydroxybenzaldehyde (0.50 g, 2.49 mmol), 4-methoxybenzeneboronic acid (0.49 g, 3.23 mmol), K_2_CO_3_ (0.86 g, 6.22 mmol) and Pd(PPh_3_)_4_ (0.14 g, 0.12 mmol). Obtained as yellow solid (0.53 g, 93%). Mp 99–100 °C.

^1^H NMR (400 MHz, CDCl_3_): δ 11.91 (1H, s), 9.85 (1H, d, *J*= 0.5 Hz), 7.51 (1H, dd, *J*= 7.7 Hz, *J*= 8.3 Hz), 7.29 (2H, d, *J*= 8.8 Hz), 6.99 (2H, d, *J*= 8.8 Hz), 6.94–6.98 (1H, m), 6.87 (1H, dd, *J*= 1.1 Hz, *J*= 7.5 Hz), 3.87 (3H, s) ppm.

^13^C NMR (100 MHz, CDCl_3_): δ 197.4, 163.0, 159.9, 147.4, 136.7, 131.4, 129.8, 121.7, 118.3, 116.6, 114.1, 55.5 ppm.

HRMS (ESI, m/z): calcd for C_14_H_13_O_3_ [M+ H]^+^ 229.0865, found 229.0863.

GC-MS (m/z, %): 128 (22), 157 (26), 185 (21), 213 (23), 227 (37), 228 (100).

IR (KBr, cm^−1^), ν_max_ = 3007 (OH), 1652 (C = O).

#### 3',4'-Dichloro-3-hydroxy-1,1'-biphenyl-2-carbaldehyde (4d)


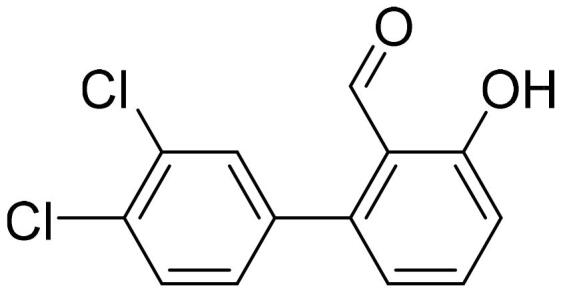


Compound **4d** was obtained from 6-bromo-2-hydroxybenzaldehyde (0.50 g, 2.49 mmol), 3,4-dichlorobenzeneboronic acid (0.62 g, 3.23 mmol), K_2_CO_3_ (0.86 g, 6.22 mmol) and Pd(PPh_3_)_4_ (0.14 g, 0.12 mmol). Obtained as white solid (0.58 g, 88%). Mp 144–145 °C.

^1^H NMR (400 MHz, CDCl_3_): δ 11.87 (1H, s), 9.82 (1H, d, *J*= 0.5 Hz), 7.52–7.58 (2H, m), 7.49 (1H, d, *J*= 2.1 Hz), 7.21 (1H, dd, *J*= 2.1 Hz, *J*= 8.3 Hz), 7.01–7.05 (1H, m), 6.85 (1H, dd, *J*= 1.1 Hz, *J*= 7.5 Hz) ppm.

^13^C NMR (100 MHz, CDCl_3_): δ 196.3, 163.1, 144.7, 137.5, 137.0, 133.1, 133.0, 131.8, 130.5, 129.4, 121.5, 118.1, 117.9 ppm.

HRMS (ESI, m/z): calcd for C_13_H_7_O_2_Cl_2_ [M-H]^-^ 264.9823, found 254.9820.

GC-MS (m/z, %): 92 (24), 120 (57), 139 (57), 168 (24), 202 (24), 230 (21), 231 (88), 233 (28), 266 (100), 267 (59), 268 (71).

IR (KBr, cm^−1^), ν_max_ = 3072 (OH), 1652 (C = O).

#### Ethyl 2'-formyl-3'-hydroxy-1,1'-biphenyl-4-carboxylate (4e)


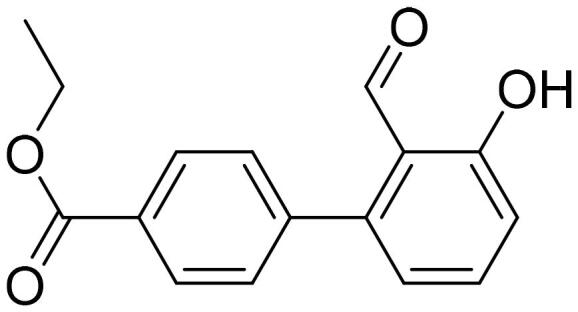


Compound **4e** was obtained from 6-bromo-2-hydroxybenzaldehyde (0.50 g, 2.49 mmol), 4-(ethoxycarbonyl)benzeneboronic acid (0.63 g, 3.23 mmol), K_2_CO_3_ (0.86 g, 6.22 mmol) and Pd(PPh_3_)_4_ (0.14 g, 0.12 mmol). Obtained as yellow solid (0.52 g, 78%). Mp 92–93 °C.

^1^H NMR (400 MHz, CDCl_3_): δ 11.89 (1H, s), 9.80 (1H, d, *J*= 0.5 Hz), 8.14 (2H, d, *J*= 8.5 Hz), 7.55 (1H, dd, *J*= 7.6 Hz, *J*= 8.3 Hz), 7.45 (2H, d, *J*= 8.5 Hz), 7.02–7.06 (1H, m), 6.89 (1H, dd, *J*= 1.1 Hz, *J*= 7.5 Hz), 4.43 (2H, q, *J*= 7.1 Hz), 1.42 (3H, t, *J*= 7.1 Hz) ppm.

^13^C NMR (100 MHz, CDCl_3_): δ 196.6, 166.2, 163.1, 146.4, 142.1, 136.8, 130.6, 130.2, 129.8, 121.5, 118.0, 117.8, 61.4, 14.5 ppm.

HRMS (ESI, m/z): calcd for C_16_H_13_O_4_ [M-H]^-^ 269.0814, found 269.0816.

GC-MS (m/z, %): 115 (22), 139 (32), 197 (100), 225 (46), 241 (46), 270 (91).

IR (KBr, cm^−1^), ν_max_ = 3056 (OH), 1716 (C = O), 1651 (C = O).

#### 3-Hydroxy-1,1'-biphenyl-4-carbaldehyde (4f)


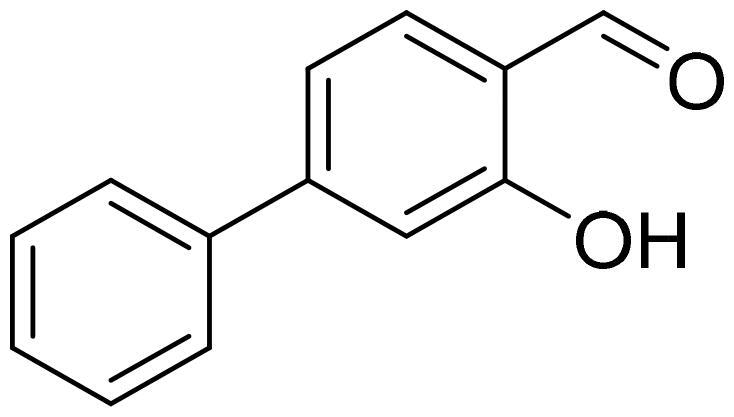


Compound **4f** was obtained from 4-bromo-2-hydroxybenzaldehyde (0.50 g, 2.49 mmol), benzeneboronic acid (0.39 g, 3.23 mmol), K_2_CO_3_ (0.86 g, 6.22 mmol) and Pd(PPh_3_)_4_ (0.14 g, 0.12 mmol). Obtained as white solid (0.45 g, 92%). Mp 81–82 °C.

^1^H NMR (400 MHz, CDCl_3_): δ 11.05 (1H, s), 9.86 (1H, d, *J*= 0.6 Hz), 7.53–7.58 (3H, m), 7.33–7.44 (3H, m), 7.19 (1H, dd, *J*= 1.6 Hz, *J*= 8.0 Hz), 7.14–7.16 (1H, m) ppm.

^13^C NMR (100 MHz, CDCl_3_): δ 196.2, 162.1, 150.0, 139.5, 134.2, 129.1, 129.0, 127.5, 119.7, 119.1, 116.0 ppm.

HRMS (ESI, m/z): calcd for C_13_H_9_O_2_ [M-H]^-^ 197.0603, found 197.0603.

GC-MS (m/z, %): 115 (22), 197 (100).

IR (KBr, cm^−1^), ν_max_ = 3057 (OH), 1683 (C = O).

#### 4'-Fluoro-3-hydroxy-1,1'-biphenyl-4-carbaldehyde (4g)


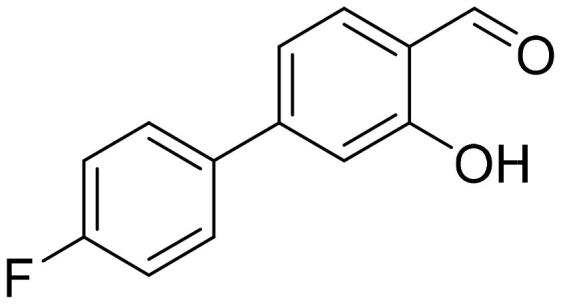


Compound **4g** was obtained from 4-bromo-2-hydroxybenzaldehyde (0.50 g, 2.49 mmol), 4-fluorobenzeneboronic acid (0.45 g, 3.23 mmol), K_2_CO_3_ (0.86 g, 6.22 mmol) and Pd(PPh_3_)_4_ (0.14 g, 0.12 mmol). Obtained as light yellow solid (0.48 g, 90%). Mp 108–109 °C.

^1^H NMR (400 MHz, CDCl_3_): δ 11.12 (1H, s), 9.92 (1H, d, *J*= 0.6 Hz), 7.57–7.63 (3H, m), 7.13–7.23 (4H, m) ppm.

^13^C NMR (100 MHz, CDCl_3_): δ 196.1, 163.5 (d, *J*= 248.6 Hz), 162.1, 148.9, 135.6 (d, *J*= 3.2 Hz), 134.3, 129.2 (d, *J*= 8.3 Hz), 119.7, 118.8, 116.1 (d, *J*= 21.5 Hz), 115.8 ppm.

HRMS (ESI, m/z): calcd for C_13_H_8_O_2_F [M-H]^-^ 215.0508, found 215.0513.

GC-MS (m/z, %): 133 (19), 215 (100).

IR (KBr, cm^−1^), ν_max_ = 3057 (OH), 1652 (C = O).

#### 3-Hydroxy-4'-methoxy-1,1'-biphenyl-4-carbaldehyde (4h)


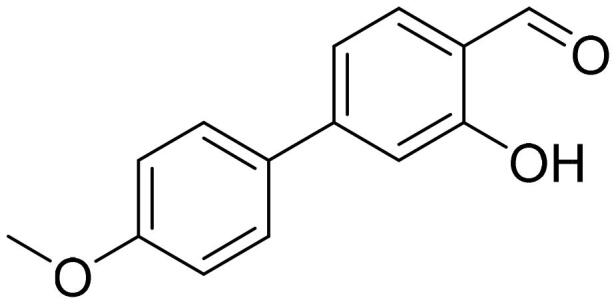


Compound **4h** was obtained from 4-bromo-2-hydroxybenzaldehyde (0.50 g, 2.49 mmol), 4-methoxybenzeneboronic acid (0.49 g, 3.23 mmol), K_2_CO_3_ (0.86 g, 6.22 mmol) and Pd(PPh_3_)_4_ (0.14 g, 0.12 mmol). Obtained as white solid (0.43 g, 75%). Mp 129–130 °C.

^1^H NMR (400 MHz, CDCl_3_): δ 11.12 (1H, s), 9.89 (1H, d, *J*= 0.4 Hz), 7.56–7.61 (3H, m), 7.22 (1H, dd, *J*= 1.7 Hz, *J*= 8.1 Hz), 7.18 (1H, d, *J*= 1.7 Hz), 7.00 (2H, d, *J*= 8.9 Hz), 3.87 (3H, s) ppm.

^13^C NMR (100 MHz, CDCl_3_): δ 195.8, 162.1, 160.6, 149.6, 134.2, 131.7, 128.7, 119.3, 118.5, 115.1, 114.6, 55.5 ppm.

HRMS (ESI, m/z): calcd for C_14_H_13_O_3_ [M+ H]^+^ 229.0865, found 229.0868.

IR (KBr, cm^−1^), ν_max_ = 3453 (OH), 1671 (C = O).

GC-MS (m/z, %): 227 (53), 228 (100).

#### Ethyl 4'-formyl-3'-hydroxy-1,1'-biphenyl-4-carboxylate (4i)


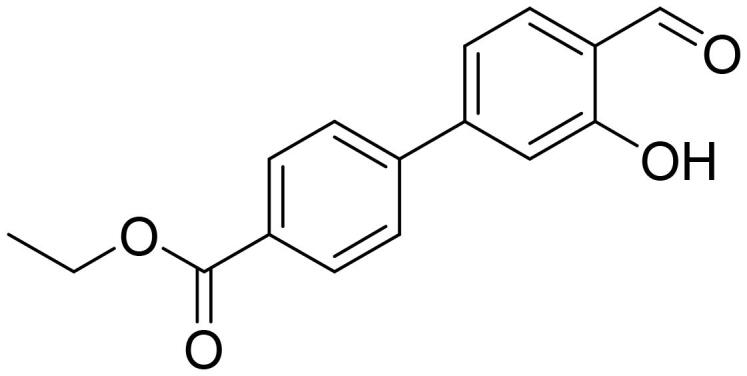


Compound **4i** was obtained from 4-bromo-2-hydroxybenzaldehyde (0.50 g, v2.49 mmol), 4-(ethoxycarbonyl)benzeneboronic acid (0.63 g, 3.23 mmol), K_2_CO_3_ (0.86 g, 6.22 mmol) and Pd(PPh_3_)_4_ (0.14 g, 0.12 mmol). Obtained as white solid (0.52 g, 78%). Mp 110–111 °C.

^1^H NMR (400 MHz, CDCl_3_): δ 11.11 (1H, s), 9.95 (1H, d, *J*= 0.6 Hz), 8.14 (2H, d, *J*= 8.6 Hz), 7.68 (2H, d, *J*= 8.6 Hz), 7.65 (1H, d, *J*= 8.0 Hz), 7.28 (1H, d, *J*= 1.7 Hz, *J*= 8.0 Hz), 7.24 (1H, d, *J*= 1.7 Hz), 4.41 (2H, q, *J*= 7.2 Hz), 1.42 (3H, t, *J*= 7.2 Hz) ppm.

^13^C NMR (100 MHz, CDCl_3_): δ 196.2, 166.3, 162.0, 148.7, 143.7, 134.3, 130.8, 130.3, 127.5, 120.1, 119.1, 116.4, 61.3, 14.5 ppm.

HRMS (ESI, m/z): calcd for C_16_H_15_O_4_ [M+ H]^+^ 271.0970, found 271.0975.

GC-MS (m/z, %): 225 (100), 242 (25), 270 (64).

IR (KBr, cm^−1^), ν_max_ = 3421 (OH), 1711 (C = O), 1665 (C = O).

#### 2-Hydroxy-1,1'-biphenyl-3-carbaldehyde (4j)


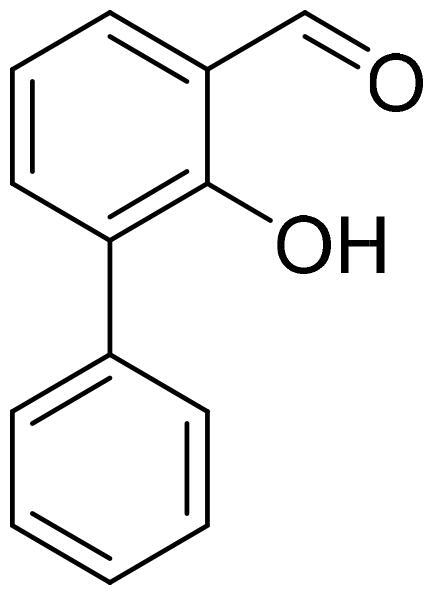


Compound **4j** was obtained from 3-bromo-2-hydroxybenzaldehyde (0.50 g, 2.49 mmol), benzeneboronic acid (0.39 g, 3.23 mmol), K_2_CO_3_ (0.86 g, 6.22 mmol) and Pd(PPh_3_)_4_ (0.14 g, 0.12 mmol). Purified by column chromatography with PE/EtOAc (10:1) → (5:1). Obtained as light yellow solid (0.39 g, 80%). Mp 46–47 °C.

^1^H NMR (400 MHz, CDCl_3_): δ 11.54 (1H, d, *J*= 0.5 Hz), 9.96 (1H, s), 7.55–7.65 (4H, m), 7.43–7.52 (2H, m), 7.34–7.41 (1H, m), 7.11 (1H, t, *J*= 7.6 Hz) ppm.

^13^C NMR (100 MHz, CDCl_3_): δ 197.0, 159.0, 138.0, 136.4, 133.3, 130.6, 129.4, 128.4, 127.8, 121.0, 120.0 ppm.

HRMS (ESI, m/z): calcd for C_13_H_11_O_2_ [M+ H]^+^ 199.0759, found 199.0759.

GC-MS (m/z, %): 115 (28), 141 (22), 152 (42), 169 (24), 197 (62), 198 (100).

IR (KBr, cm^−1^), ν_max_ = 3051 (OH), 1656 (C = O).

#### 4'-Fluoro-2-hydroxy-1,1'-biphenyl-3-carbaldehyde (4k)


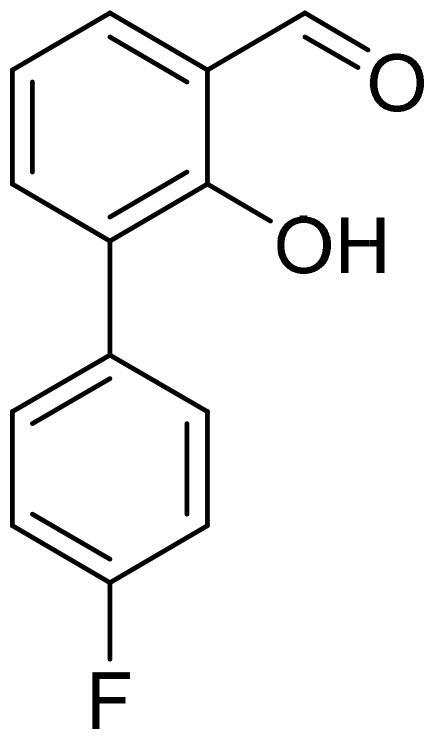


Compound **4k** was obtained from 3-bromo-2-hydroxybenzaldehyde (0.50 g, 2.49 mmol), 4-fluorobenzeneboronic acid (0.45 g, 3.23 mmol), K_2_CO_3_ (0.86 g, 6.22 mmol), and Pd(PPh_3_)_4_ (0.14 g, 0.12 mmol). Obtained as light yellow solid (0.42 g, 78%). Mp 95–96 °C.

^1^H NMR (400 MHz, CDCl_3_): δ 11.55 (1H, d, *J*= 0.5 Hz), 9.96 (1H, s), 7.54–7.61 (4H, m), 7.08–7.18 (3H, m) ppm.

^13^C NMR (100 MHz, CDCl_3_): δ 196.9, 162.5 (d, *J*= 247.3 Hz), 158.9, 137.8, 133.4, 132.3 (d, *J*= 3.8 Hz), 131.1 (d, *J*= 7.9 Hz), 129.6, 121.0, 120.1, 115.4 (d, *J*= 21.3 Hz) ppm.

HRMS (ESI, m/z): calcd for C_13_H_8_O_2_F [M-H]^-^ 215.0508, found 215.0509.

GC-MS (m/z, %): 138 (25), 159 (24), 170 (33), 215 (60), 216 (100).

IR (KBr, cm^−1^), ν_max_ = 3039 (OH), 1658 (C = O).

#### 2-Hydroxy-4'-methoxy-1,1'-biphenyl-3-carbaldehyde (4l)


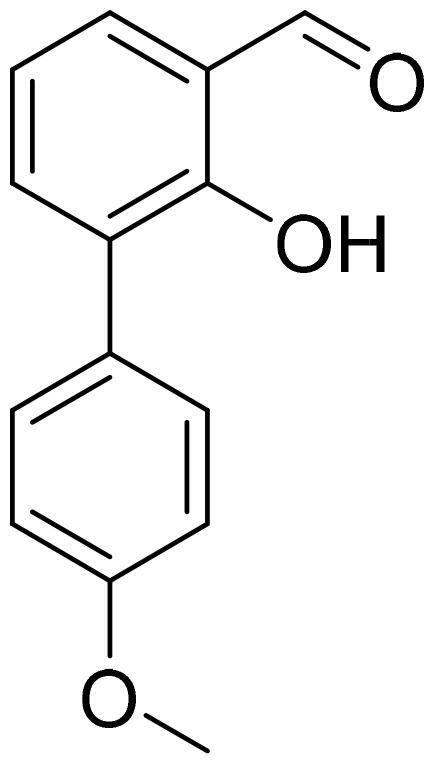


Compound **4l** was obtained from 3-bromo-2-hydroxybenzaldehyde (0.50 g, 2.49 mmol), 4-methoxybenzeneboronic acid (0.49 g, 3.23 mmol), K_2_CO_3_ (0.86 g, 6.22 mmol) and Pd(PPh_3_)_4_ (0.14 g, 0.12 mmol). Obtained as light yellow solid (0.47 g, 83%). Mp 81–82 °C.

^1^H NMR (400 MHz, CDCl_3_): δ 11.53 (1H, d, *J*= 0.6 Hz), 9.95 (1H, s), 7.60 (1H, ddd, *J*= 0.6 Hz, *J*= 1.7 Hz, *J*= 7.6 Hz), 7.54 (2H, d, *J*= 8.8 Hz), 7.53 (1H, dd, *J*= 1.7 Hz, *J*= 7.6 Hz), 7.09 (1H, t, *J*= 7.6 Hz), 6.99 (2H, d, *J*= 8.8 Hz), 3.86 (3H, s) ppm.

^13^C NMR (100 MHz, CDCl_3_): δ 197.0, 159.3, 159.0, 137.6, 132.9, 130.5, 130.3, 128.8, 121.0, 120.0, 113.9, 55.5 ppm.

HRMS (ESI, m/z): calcd for C_14_H_13_O_3_ [M+ H]^+^ 229.0865, found 229.0870.

GC-MS (m/z, %): 128 (22), 228 (100).

IR (KBr, cm^−1^), ν_max_ = 3037 (OH), 1658 (C = O).

#### 3',4'-Dichloro-2-hydroxy-1,1'-biphenyl-3-carbaldehyde (4m)


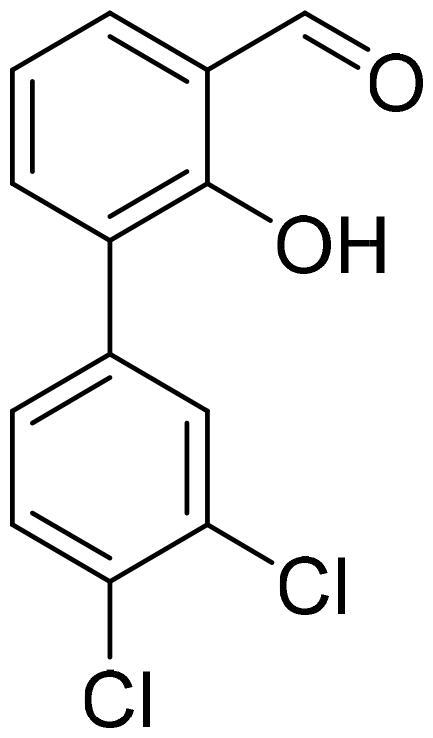


Compound **4m** was obtained from 3-bromo-2-hydroxybenzaldehyde (0.50 g, 2.49 mmol), 3,4-dichlorobenzeneboronic acid (0.62 g, 3.23 mmol), K_2_CO_3_ (0.86 g, 6.22 mmol), and Pd(PPh_3_)_4_ (0.14 g, 0.12 mmol). Purified by column chromatography with PE/EtOAc (10:1) → (5:1). Obtained as white solid (0.57 g, 85%). Mp 143–144 °C.

^1^H NMR (400 MHz, CDCl_3_): δ 11.59 (1H, d, *J*= 0.5 Hz), 9.96 (1H, s), 7.71 (1H, d, *J*= 2.1 Hz), 7.57–7.63 (2H, m), 7.51 (1H, d, *J*= 8.3 Hz), 7.45 (1H, dd, *J*= 2.1 Hz, *J*= 8.3 Hz), 7.12 (1H, t, *J*= 7.7 Hz) ppm.

^13^C NMR (100 MHz, CDCl_3_): δ 196.9, 158.9, 137.5, 136.3, 134.1, 132.5, 131.9, 131.3, 130.3, 128.7, 128.1, 121.1, 120.2 ppm.

HRMS (ESI, m/z): calcd for C_13_H_7_O_2_Cl_2_ [M-H]^-^ 264.9823, found 264.9823.

GC-MS (m/z, %): 139 (31), 168 (23), 202 (23), 220 (18), 265 (43), 266 (100), 268 (63).

IR (KBr, cm^−1^), ν_max_ = 3045 (OH), 1657 (C = O).

#### General procedure for the preparation of 1,2,3-benzoxathiazine 2,2-dioxides 5a-m

The derivative of 2-hydroxybenzaldehyde **4a–m** (1 equiv) was dissolved in dry DMA (6 ml). Reaction mixture was cooled to 0 °C. Sulfamoyl chloride (2.5 equiv) was slowly added to the reaction mixture. The stirring was continued at room temperature under argon atmosphere for 24 h. Reaction mixture was then poured into ice-water (25 ml), extracted with DCM (3× 25 ml), washed with satd. NaHCO_3_ (3× 25 ml) and brine (3× 25 ml). Organic phase was dried over Na_2_SO_4_, filtered and evaporated. The product was purified by column chromatography with PE/EtOAc (2:1) followed by recrystallisation from EtOH.

#### 5-Phenyl-1,2,3-benzoxathiazine 2,2-dioxide (5a)


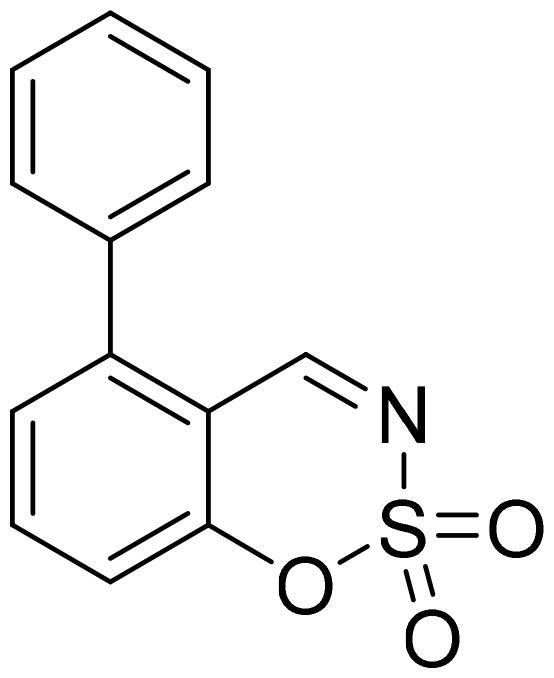


Compound **5a** was obtained from 3-hydroxy-1,1′-biphenyl-2-carbaldehyde (**4a**) (0.20 g, 1.01 mmol) and sulfamoyl chloride (0.29 g, 2.53 mmol). Obtained as white solid (0.12 g, 46%). Mp 156–157 °C.

^1^H NMR (400 MHz, CDCl_3_): δ 8.59 (1H, d, *J*= 0.4 Hz), 7.77 (1H, dd, *J*= 7.8 Hz, *J*= 8.3 Hz), 7.51–7.57 (3H, m), 7.37–7.43 (3H, m), 7.28–7.32 (1H, m) ppm.

^13^C NMR (100 MHz, CDCl_3_): δ 167.7, 155.0, 145.7, 137.0, 135.7, 130.2, 129.6, 129.3, 127.4, 117.5, 114.1 ppm.

HRMS (ESI, m/z): calcd for C_13_H_10_NO_3_S [M+ H]^+^ 260.0381, found 260.0381.

GC-MS (m/z, %): 139 (42), 166 (39), 167 (80), 195 (30), 259 (100).

IR (KBr, cm^−1^), ν_max_ = 1389 (S = O), 1193 (S = O), 1183 (S = O).

#### 5-(4-Fluorophenyl)-1,2,3-benzoxathiazine 2,2-dioxide (5b)


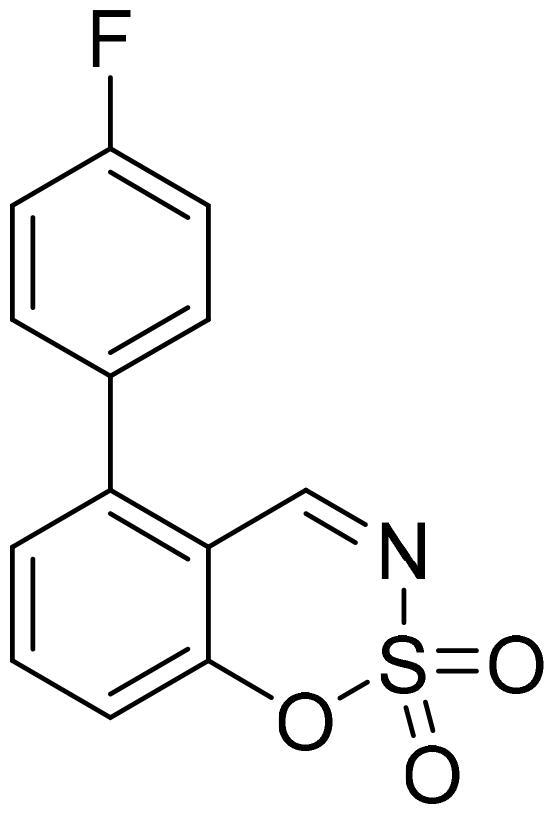


Compound **5b** was obtained from 4′-fluoro-3-hydroxy-1,1′-biphenyl-2-carbaldehyde (**4b**) (0.28 g, 1.28 mmol) and sulfamoyl chloride (0.37 g, 3.20 mmol). Reaction mixture was stirred for 48 h. Obtained as white solid (0.14 g, 42%). Mp 186–187 °C.

^1^H NMR (400 MHz, CDCl_3_): δ 8.56 (1H, d, *J*= 0.4 Hz), 7.77 (1H, dd, *J*= 7.8 Hz, *J*= 8.3 Hz), 7.35–7.42 (3H, m), 7.29–7.33 (1H, m), 7.21–7.28 (2H, m) ppm.

^13^C NMR (100 MHz, CDCl_3_): δ 167.3, 163.7 (d, *J*= 250.8 Hz), 155.1, 144.5, 137.1, 132.0 (d, *J*= 8.4 Hz), 131.8 (d, *J*= 3.1 H), 127.4, 117.7, 116.5 (d, *J*= 22.1 Hz), 114.1 ppm.

HRMS (ESI, m/z): calcd for C_13_H_9_NO_3_SF [M+ H]^+^ 278.0287, found 278.0283.

GC-MS (m/z, %): 157 (41), 184 (37), 185 (72), 213 (31), 277 (100).

IR (KBr, cm^−1^), ν_max_ = 1388 (S = O), 1191 (S = O).

#### 5–(4-Methoxyphenyl)-1,2,3-benzoxathiazine 2,2-dioxide (5c)


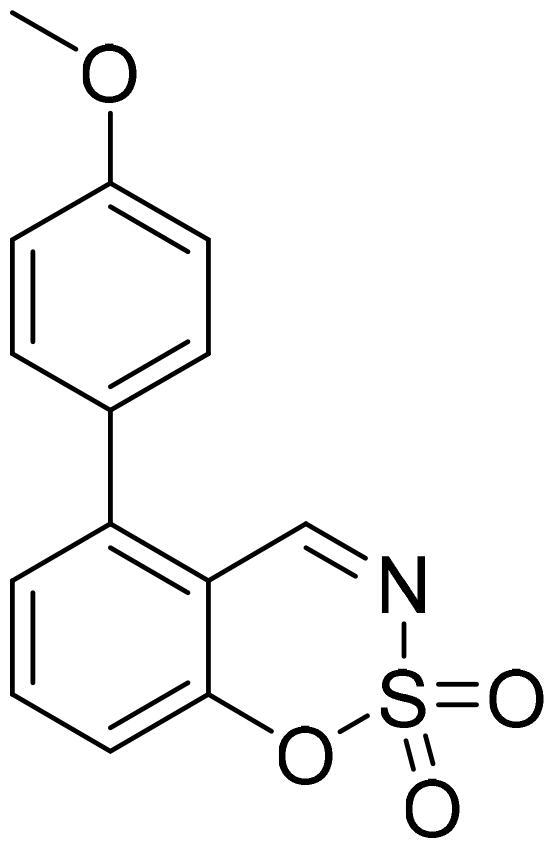


Compound **5c** was obtained from 4′-methoxy-3-hydroxy-1,1′-biphenyl-2-carbaldehyde (**4c**) (0.30 g, 1.31 mmol) and sulfamoyl chloride (0.38 g, 3.28 mmol). Obtained as yellow solid (0.10 g, 26%). Mp 177–178 °C.

^1^H NMR (400 MHz, CDCl_3_): δ 8.59 (1H, d, *J*= 0.5 Hz), 7.74 (1H, dd, *J*= 7.8 Hz, *J*= 8.3 Hz), 7.38 (1H, dd, *J*= 1.1 Hz, *J*= 7.8 Hz), 7.31 (2H, d, *J*= 8.8 Hz), 7.23–7.27 (1H, m), 7.06 (2H, d, *J*= 8.8 Hz), 3.89 (3H, s) ppm.

^13^C NMR (100 MHz, CDCl_3_): δ 168.0, 160.9, 155.1, 145.6, 137.0, 131.6, 128.0, 127.2, 116.9, 114.8, 114.1, 55.7 ppm.

HRMS (ESI, m/z): calcd for C_14_H_12_NO_4_S [M+ H]^+^ 290.0487, found 290.0494.

GC-MS (m/z, %): 127 (22), 154 (28), 182 (23), 289 (100).

IR (KBr, cm^−1^), ν_max_ = 1378 (S = O), 1376 (S = O), 1184 (S = O).

#### 5-(3,4-Dichlorophenyl)-1,2,3-benzoxathiazine 2,2-dioxide (5d)


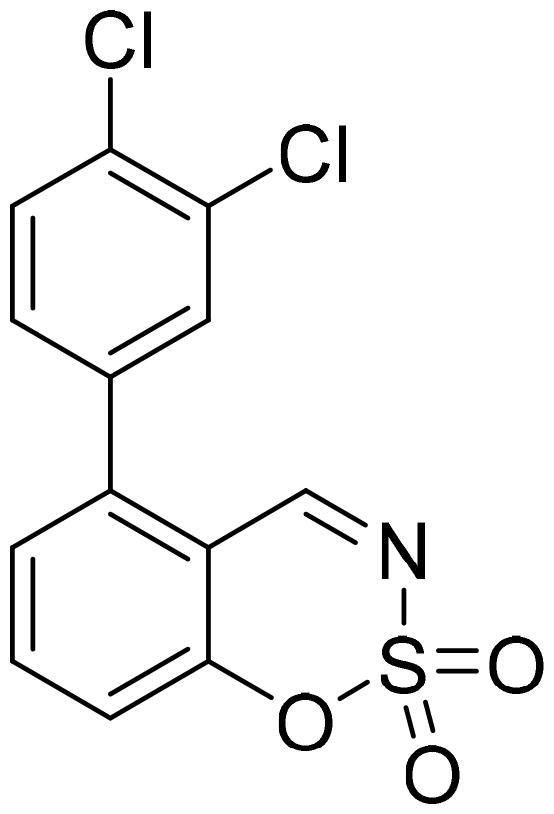


Compound **5d** was obtained from 3′,4′-dichloro-3-hydroxy-1,1′-biphenyl-2-carbaldehyde (**4d**) (0.30 g, 1.12 mmol) and sulfamoyl chloride (0.32 g, 2.8 mmol). Obtained as white solid (0.12 g, 32%). Mp 230–231 °C.

^1^H NMR (400 MHz, CDCl_3_): δ 8.56 (1H, d, *J*= 0.5 Hz), 7.79 (1H, dd, *J*= 7.8 Hz, *J*= 8.3 Hz), 7.63 (1H, d, *J*= 8.3 Hz), 7.52 (1H, d, *J*= 2.2 Hz), 7.33–7.39 (2H, m), 7.22 (1H, dd, *J*= 2.2 Hz, *J*= 8.2 Hz) ppm.

^13^C NMR (100 MHz, CDCl_3_): δ 166.7, 155.1, 142.7, 137.3, 135.5, 134.6, 133.9, 131.7, 131.3, 129.4, 127.3, 118.5, 113.9 ppm.

HRMS (ESI, m/z): calcd for C_13_H_8_NO_3_SCl_2_ [M+ H]^+^ 327.9602, found 327.9605.

IR (KBr, cm^−1^), ν_max_ = 1384 (S = O), 1190 (S = O), 1166 (S = O).

#### Ethyl 4-(2,2-dioxido-1,2,3-benzoxathiazin-5-yl)benzoate (5e)


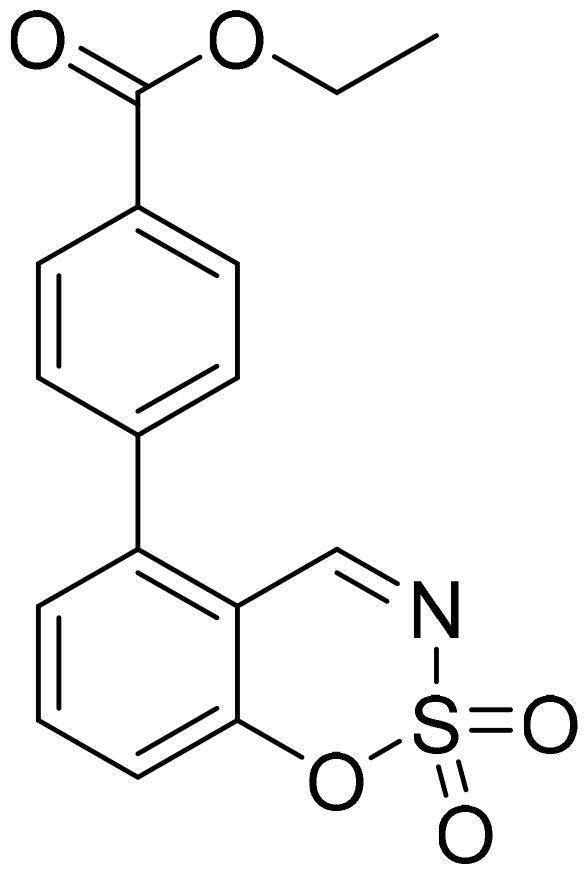


Compound **5e** was obtained from ethyl 2′-formyl-3′-hydroxy-1,1′-biphenyl-4-carboxylate (**4e**) (0.20 g, 0.74 mmol) and sulfamoyl chloride (0.21 g, 1.85 mmol). Reaction mixture was stirred for 48 h. Obtained as white solid (0.08 g, 34%). Mp 135–136 °C.

^1^H NMR (400 MHz, CDCl_3_): δ 8.55 (1H, d, *J*= 0.5 Hz), 8.21 (2H, d, *J*= 8.4 Hz), 7.80 (1H, dd, *J*= 7.8 Hz, *J*= 8.4 Hz), 7.48 (2H, d, *J*= 8.4 Hz), 7.41 (1H, dd, *J*= 1.1 Hz, *J*= 7.7 Hz), 7.33–7.36 (1H, m), 4.42 (2H, q, *J*= 7.1 Hz), 1.44 (3H, t, *J*= 7.1 Hz) ppm.

^13^C NMR (100 MHz, CDCl_3_): δ 167.0, 165.9, 155.1, 144.4, 139.9, 137.2, 131.7, 130.4, 130.2, 127.3, 118.2, 114.0, 61.6, 14.5 ppm.

HRMS (ESI, m/z): calcd for C_16_H_14_NO_5_S [M+ H]^+^ 332.0593, found 332.0590.

GC-MS (m/z, %): 216 (20), 253 (100), 254 (23), 331 (40).

IR (KBr, cm^−1^), ν_max_ = 1715 (C = O), 1383 (S= O), 1197 (S= O).

#### 7-Phenyl-1,2,3-benzoxathiazine 2,2-dioxide (5f)


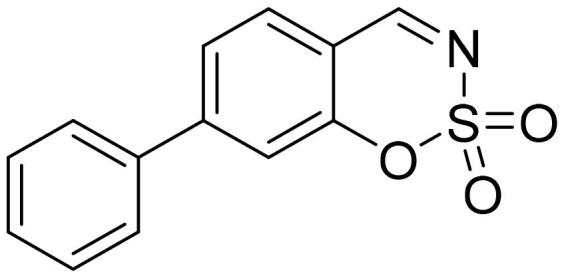


Compound **5f** was obtained from ethyl 3-hydroxy-1,1′-biphenyl-4-carbaldehyde (**4f**) (0.20 g, 1.01 mmol) and sulfamoyl chloride (0.29 g, 2.52 mmol). Obtained as white solid (0.14 g, 55%). Mp 171–172 °C.

^1^H NMR (400 MHz, CDCl_3_): δ 8.68 (1H, d, *J*= 0.4 Hz), 7.73 (1H, d, *J*= 8.1 Hz), 7.61–7.66 (3H, m), 7.48–7.55 (4H, m) ppm.

^13^C NMR (100 MHz, CDCl_3_): δ 167.4, 154.9, 151.2, 137.9, 131.3, 130.0, 129.5, 127.5, 124.8, 116.8, 114.1 ppm.

HRMS (ESI, m/z): calcd for C_13_H_10_NO_3_S [M+ H]^+^ 260.0381, found 260.0382.

GC-MS (m/z, %): 139 (50), 167 (24), 195 (57), 259 (100).

IR (KBr, cm^−1^), ν_max_ = 1379 (S= O), 1193 (S= O).

#### 7-(4-Fluorophenyl)-1,2,3-benzoxathiazine 2,2-dioxide (5g)


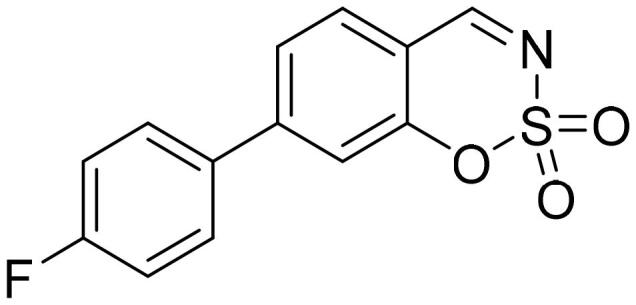


Compound **5g** was obtained from 4′-fluoro-3-hydroxy-1,1′-biphenyl-4-carbaldehyde (**4g**) (0.20 g, 0.93 mmol) and sulfamoyl chloride (0.27 g, 2.31 mmol). The product was purified by column chromatography with PE/EtOAc (1:1). Obtained as white solid (0.11 g, 43%). Mp 164–165 °C.

^1^H NMR (400 MHz, CDCl_3_): δ 8.68 (1H, d, *J*= 0.4 Hz), 7.72 (1H, d, *J*= 8.1 Hz), 7.57–7.65 (3H, m), 7.45 (1H, d, *J*= 1.6 Hz), 7.22 (2H, t, *J*= 8.6 Hz) ppm.

^13^C NMR (100 MHz, CDCl_3_): δ 167.3, 164.0 (d, *J*= 251.1 Hz), 154.9, 150.0, 134.1 (d, *J*= 3.4 Hz), 131.3, 129.4 (d, *J*= 8.6 Hz), 124.7, 116.6, 116.7 (d, *J*= 21.4 Hz), 114.1 ppm.

HRMS (ESI, m/z): calcd for C_13_H_9_NO_3_SF [M+ H]^+^ 278.0287, found 278.0288.

GC-MS (m/z, %): 157 (57), 158 (21), 185 (28), 213 (36), 277 (100).

IR (KBr, cm^−1^), ν_max_ = 1381 (S = O), 1195 (S = O), 1162 (S = O).

#### 7-(4-Methoxyphenyl)-1,2,3-benzoxathiazine 2,2-dioxide (5h)


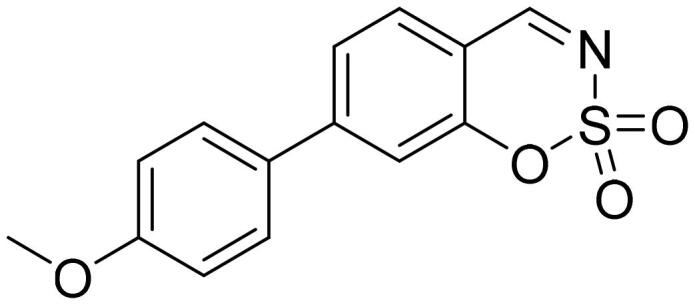


Compound **5h** was obtained from 3-hydroxy-4′-methoxy-1,1′-biphenyl-4-carbaldehyde (**4h**) (0.20 g, 0.88 mmol) and sulfamoyl chloride (0.25 g, 2.19 mmol). The product was purified by column chromatography with PE/EtOAc (1:1). Obtained as light yellow solid (0.07 g, 25%). Mp 181–182 °C.

^1^H NMR (400 MHz, CDCl_3_): δ 8.65 (1H, d, *J*= 0.5 Hz), 7.68 (1H, d, *J*= 8.0 Hz), 7.57–7.62 (3H, m), 7.45 (1H, d, *J*= 1.7 Hz), 7.03 (2H, d, *J*= 8.8 Hz), 3.88 (3H, s) ppm.

^13^C NMR (100 MHz, CDCl_3_): δ 167.3, 161.4, 155.0, 150.8, 131.2, 130.1, 128.9, 124.1, 115.9, 115.0, 113.6, 55.6 ppm.

HRMS (ESI, m/z): calcd for C_14_H_12_NO_4_S [M+ H]^+^ 290.0487, found 290.0498.

IR (KBr, cm^−1^), ν_max_ = 1377 (S= O), 1363 (S= O), 1188 (S= O).

#### Ethyl 4-(2,2-dioxido-1,2,3-benzoxathiazin-7-yl)benzoate (5i)


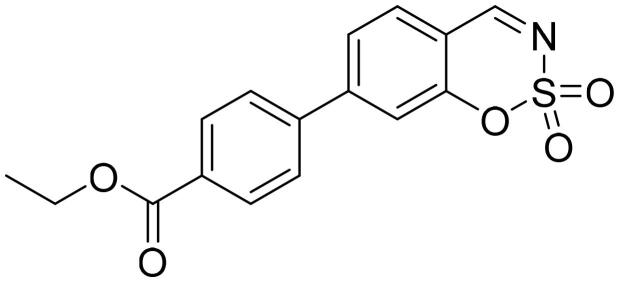


Compound **5i** was obtained from ethyl 4′-formyl-3′-hydroxy-1,1′-biphenyl-4-carboxylate (**4i**) (0.20 g, 0.74 mmol) and sulfamoyl chloride (0.21 g, 1.85 mmol). The product was purified by column chromatography with PE/EtOAc (1:1). Obtained as white solid (0.16 g, 64%). Mp 204–205 °C.

^1^H NMR (400 MHz, CDCl_3_): δ 8.71 (1H, d, *J*= 0.4 Hz), 8.18 (2H, d, *J*= 8.6 Hz), 7.76 (1H, d, *J*= 8.0 Hz), 7.69 (2H, d, *J*= 8.6 Hz), 7.66 (1H, dd, *J*= 1.6 Hz, *J*= 8.0 Hz), 7.53 (1H, d, *J*= 1.6 Hz), 4.43 (2H, q, *J*= 7.1 Hz), 1.43 (3H, t, *J*= 7.1 Hz) ppm.

^13^C NMR (100 MHz, CDCl_3_): δ 167.3, 166.0, 154.9, 149.8, 142.0, 131.7, 131.3, 130.6, 127.5, 125.0, 117.2, 114.7, 61.5, 14.5 ppm.

HRMS (ESI, m/z): calcd for C_16_H_14_NO_5_S [M+ H]^+^ 332.0593, found 332.0611.

IR (KBr, cm^−1^), ν_max_ = 1701 (C = O), 1377 (S= O), 1194 (S= O).

#### 8-Phenyl-1,2,3-benzoxathiazine 2,2-dioxide (5j)


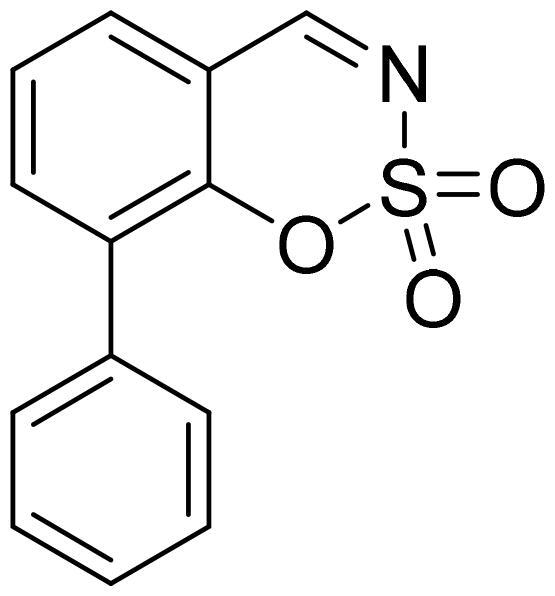


Compound **5j** was obtained from 2-hydroxy-1,1′-biphenyl-3-carbaldehyde (**4j**) (0.20 g, 1.01 mmol) and sulfamoyl chloride (0.29 g, 2.52 mmol). The product was purified by column chromatography with PE/EtOAc (1:1). Obtained as white solid (0.12 g, 44%). Mp 164–165 °C.

^1^H NMR (400 MHz, CDCl_3_): δ 8.71 (1H, s), 7.80 (1H, dd, *J*= 1.6 Hz, *J*= 7.7 Hz), 7.66 (1H, dd, *J*= 1.6 Hz, *J*= 7.7 Hz), 7.42–7.56 (6H, m) ppm.

^13^C NMR (100 MHz, CDCl_3_): δ 168.1, 151.1, 138.8, 133.4, 132.8, 130.0, 129.5, 129.0, 128.9, 126.3, 116.1 ppm.

HRMS (ESI, m/z): calcd for C_13_H_10_NO_3_S [M+ H]^+^ 260.0381, found 260.0379.

GC-MS (m/z, %): 139 (64), 140 (21), 168 (42), 194 (74), 195 (53), 259 (100).

IR (KBr, cm^−1^), ν_max_ = 1387 (S= O), 1174 (S= O).

#### 8-(4-Fluorophenyl)-1,2,3-benzoxathiazine 2,2-dioxide (5k)


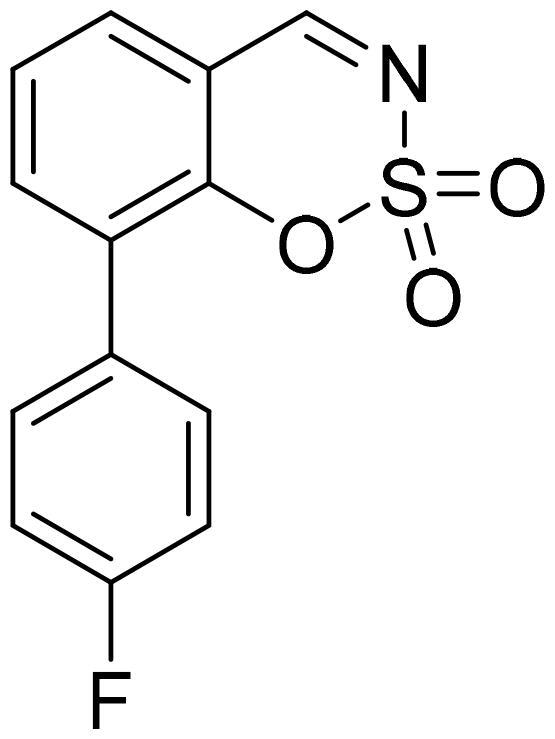


Compound **5k** was obtained from 4′-fluoro-2-hydroxy-1,1′-biphenyl-3-carbaldehyde (**4k**) (0.20 g, 0.93 mmol) and sulfamoyl chloride (0.27 g, 2.31 mmol). The product was purified by column chromatography with PE/EtOAc (1:1). Obtained as white solid (0.14 g, 54%). Mp 123–124 °C.

^1^H NMR (400 MHz, DMSO-d_6_): δ 9.29 (1H, s), 8.04 (1H, dd, *J*= 1.6 Hz, *J*= 7.7 Hz), 7.98 (1H, dd, *J*= 1.6 Hz, *J*= 7.7 Hz), 7.65 (1H, t, *J*= 7.7 Hz), 7.57–7.63 (2H, m), 7.41 (2H, t, *J*= 8.9 Hz) ppm.

^13^C NMR (100 MHz, DMSO-d_6_): δ 171.3, 162.4 (d, *J*= 246.2 Hz), 149.7, 138.9, 131.8, 131.4 (d, *J*= 8.3 Hz), 129.8, 129.6 (d, *J*= 3.1 Hz), 126.7 ppm.

HRMS (ESI, m/z): calcd for C_13_H_9_NO_3_SF [M+ H]^+^ 278.0287, found 278.0294.

GC-MS (m/z, %): 157 (66), 158 (21), 184 (20), 186 (31), 213 (64), 277 (100).

IR (KBr, cm^−1^), ν_max_ = 1383 (S= O), 1171 (S= O).

#### 8-(4-Methoxyphenyl)-1,2,3-benzoxathiazine 2,2-dioxide (5l)


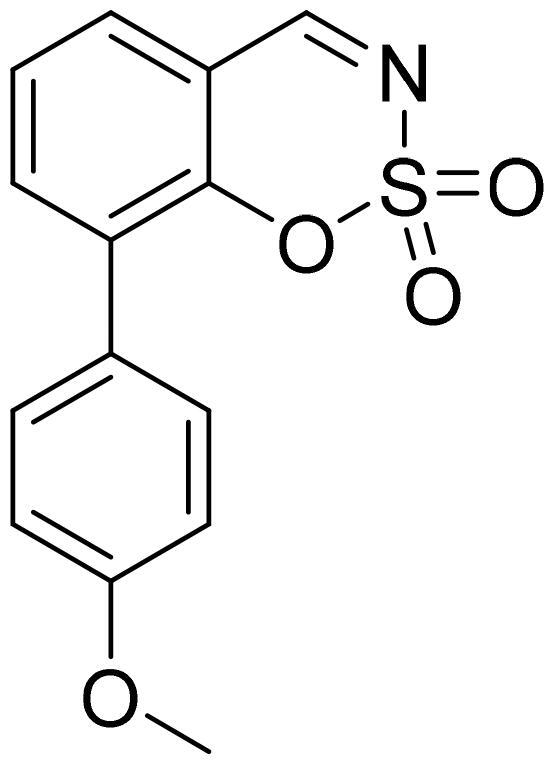


Compound **5l** was obtained from 2-hydroxy-4′-methoxy-1,1′-biphenyl-3-carbaldehyde (**4l**) (0.20 g, 0.88 mmol) and sulfamoyl chloride (0.25 g, 2.19 mmol). The product was purified by column chromatography with PE/EtOAc (1:1). Obtained as yellow solid (0.11 g, 44%). Mp 133–134 °C.

^1^H NMR (400 MHz, CDCl_3_): δ 8.70 (1H, s), 7.77 (1H, dd, *J*= 1.7 Hz, *J*= 7.7 Hz), 7.61 (1H, dd, *J*= 1.7 Hz, *J*= 7.7 Hz), 7.44–7.51 (3H, m), 7.02 (2H, d, *J*= 9.0 Hz), 3.87 (3H, s) ppm.

^13^C NMR (100 MHz, CDCl_3_): δ 168.2, 160.3, 151.0, 138.5, 132.4, 130.7, 129.4, 126.2, 125.6, 116.2, 114.5, 55.5 ppm.

HRMS (ESI, m/z): calcd for C_14_H_12_NO_4_S [M+ H]^+^ 290.0487, found 290.0488.

GC-MS (m/z, %): 177 (49), 155 (21), 183 (23), 198 (23), 210 (39), 225 (31), 289 (100).

IR (KBr, cm^−1^), ν_max_ = 1373 (S = O), 1370 (S = O), 1180 (S = O).

#### 8-(3,4-Dichlorophenyl)-1,2,3-benzoxathiazine 2,2-dioxide (5m)


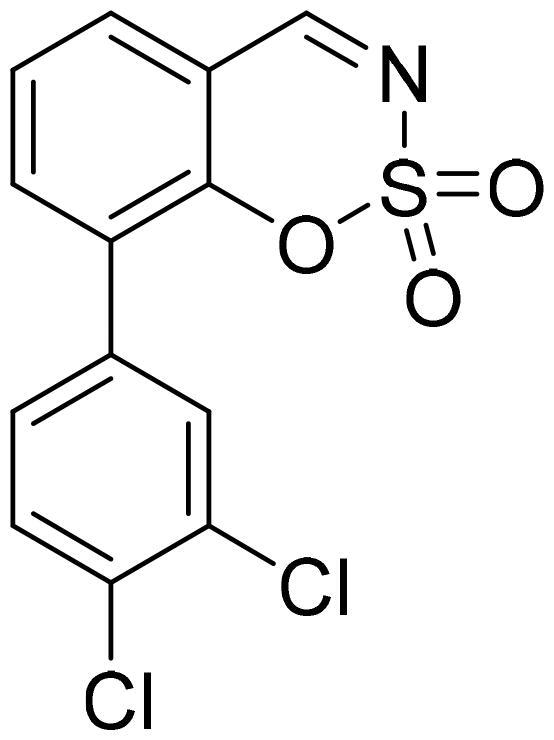


Compound **5m** was obtained from 3′,4′-dichloro-2-hydroxy-1,1′-biphenyl-3-carbaldehyde (**4m**) (0.20 g, 0.75 mmol) and sulfamoyl chloride (0.22 g, 1.88 mmol). The product was purified by column chromatography with PE/EtOAc (1:2). Obtained as white solid (0.16 g, 65%). Mp 165–166 °C.

^1^H NMR (400 MHz, DMSO-d_6_): δ 9.31 (1H, s), 8.08 (1H, d, *J*= 1.6 Hz, *J*= 7.7 Hz), 8.04 (1H, d, *J*= 1.6 Hz, *J*= 7.7 Hz), 7.82–7.87 (2H, m), 7.67 (1H, t, *J*= 7.8 Hz), 7.55 (1H, dd, *J*= 2.1 Hz, *J*= 8.3 Hz) ppm.

^13^C NMR (100 MHz, DMSO-d_6_): δ 171.3, 149.7, 138.9, 133.7, 132.5, 131.8, 131.6, 131.0, 129.5, 128.2, 126.8, 115.8 ppm.

HRMS (ESI, m/z): calcd for C_13_H_8_NO_3_SCl_2_ [M+ H]^+^ 327.9602, found 327.9609.

IR (KBr, cm^−1^), ν_max_ = 1393 (S = O), 1180 (S = O).

### Ca inhibitory assay

An applied photophysics stopped-flow instrument has been used for assaying the CA catalysed CO_2_ hydration activity.^32^

Phenol red (at a concentration of 0.2 mM) was used as indicator, working at the absorbance maximum of 557 nm, with 20 mM Hepes (pH 7.5), and 20 mM Na_2_SO_4_ (for maintaining constant the ionic strength), following the initial rates of the CA-catalysed CO_2_ hydration reaction for a period of 10 − 100 s. The CO_2_ concentrations ranged from 1.7 to 17 mM for the determination of the kinetic parameters and inhibition constants. For each inhibitor, at least six traces of the initial 5 − 10% of the reaction have been used for determining the initial rate. The uncatalysed rates were determined in the same manner and subtracted from the total observed rates. Stock solutions of inhibitor (0.1 mM) were prepared in distilled – deionised water, and dilutions up to 0.01 nM were done thereafter with the assay buffer. Inhibitor and enzyme solutions were preincubated together for 15 min at room temperature prior to assay in order to allow for the formation of the E – I complex. Data from Table 1 were obtained after 6 h incubation of enzyme and inhibitor. The inhibition constants were obtained by nonlinear least-squares methods using PRISM 3 and the Cheng – Prusoff equation, as reported earlier^15^^,^^33–37^^,^ and represent the mean from at least three different determinations. All CA isoforms were recombinant ones obtained in-house as reported earlier^12^^,^^18^^,^^38–45^.

**Table 1.** Inhibition data of human CA isoforms hCA I, II, IX, XII with compounds **JI** reported here and the standard inhibitor acetazolamide (AAZ) by a stopped flow CO_2_ hydrase assay.

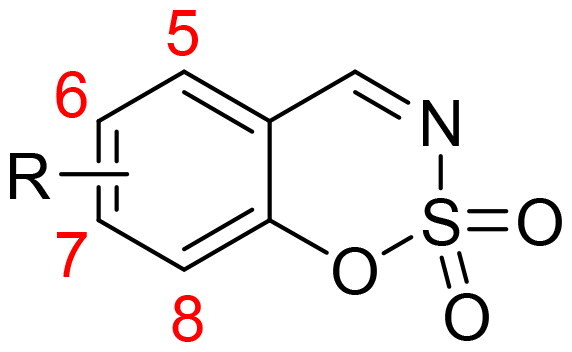					
cmp	R	K_I_ (nM)^a,b^			
		hCA I	hCA II	hCA IX	hCA XII
**2a**	6-Me	25690	115.6	72.0	98.2
**2b**	6-MeO	>100,000	168.5	62.8	75.1
**2c**	6-F	3324	71.8	58.3	120.8
**2d**	6-Br	>100,000	8315	27.4	186.4
**2e**	7-Me	657.1	78.9	53.1	58.0
**2f**	7-MeO	986.5	55.2	39.7	83.1
**2g**	7-F	447.6	86.7	55.4	111.7
**2h**	7-Br	46520	45.1	22.3	70.1
**2i**	8-MeO	1523	39.0	73.6	63.4
**2j**	8-F	465.8	89.2	65.9	34.7
**2k**	8-Br	788.1	112.8	41.8	86.3
**5a**	5-Ph	>100,000	9865	5247	12630
**5b**	5-(4-F-C_6_H_4_)	>100,000	14,830	4139	7465
**5c**	5-(4-OMe-C_6_H_4_)	>100,000	36,870	10,870	17,960
**5d**	5-(3,4-Cl_2_-C_6_H_3_)	>100,000	>100,000	>100,000	45,320
**5e**	5-(4-CO_2_Et-C_6_H_4_)	>100,000	>100,000	>100,000	>100,000
**5f**	7-Ph	5682	7.7	28.6	78.3
**5g**	7-(4-F-C_6_H_4_)	15,230	2.1	69.8	8.2
**5h**	7-(4-OMe-C_6_H_4_)	30,310	14.3	45.2	36.4
**5i**	7-(4-CO_2_Et-C_6_H_4_)	356.3	0.8	38.0	90.5
**5j**	8-Ph	668.4	0.5	49.1	61.8
**5k**	8-(4-F-C_6_H_4_)	1852	5.6	32.3	12.4
**5l**	8-(4-OMe-C_6_H_4_)	4259	12.3	62.7	7.3
**5m**	8-(3,4-Cl_2_-C_6_H_3_)	6708	9.5	40.8	4.8
**AAZ**	–	250.0	12.5	25.0	5.7

^a^Mean from three different assays, by a stopped flow technique (errors were in the range of ± 5–10% of the reported values); ^b^15 min incubation.

## Results and discussion

### Chemistry

**Scheme 1.**
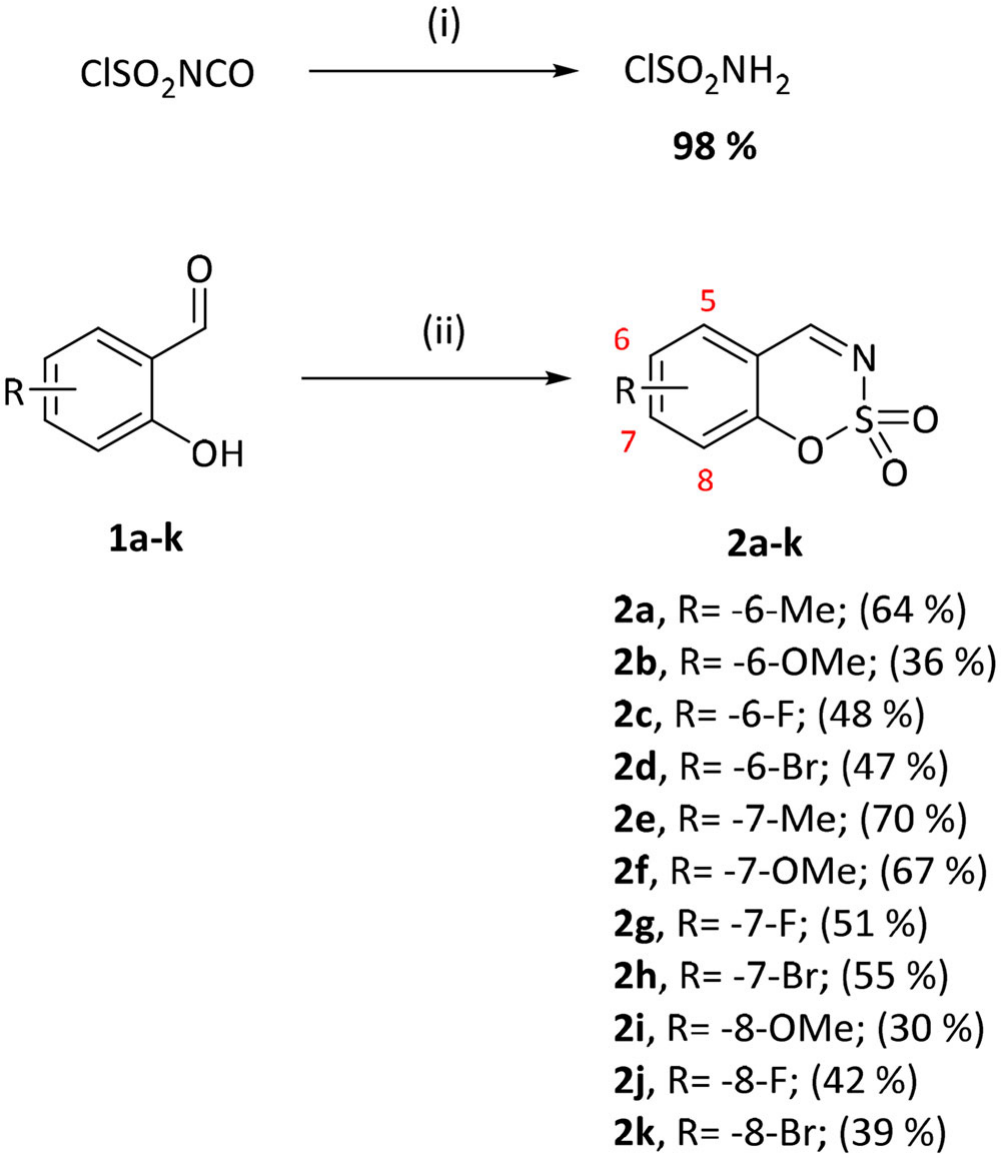
Reagents and conditions: (i) HCOOH, 0 °C, 15 min, then RT, 45 min; (ii) ClSO_2_NH_2_, DMA, RT, 24–72 h.

Benzo[1,2,3]oxathiazine-2,2-dioxides **2a–-k** were obtained from corresponding 2-hydroxybenzaldehydes **1a–k** in their reaction with sulfamoyl chloride that was prepared from chlorosulfonyl isocyanate (Scheme 1).^46^

Eleven target compounds were obtained with moderate yields (Table 1).

**Scheme 2.**
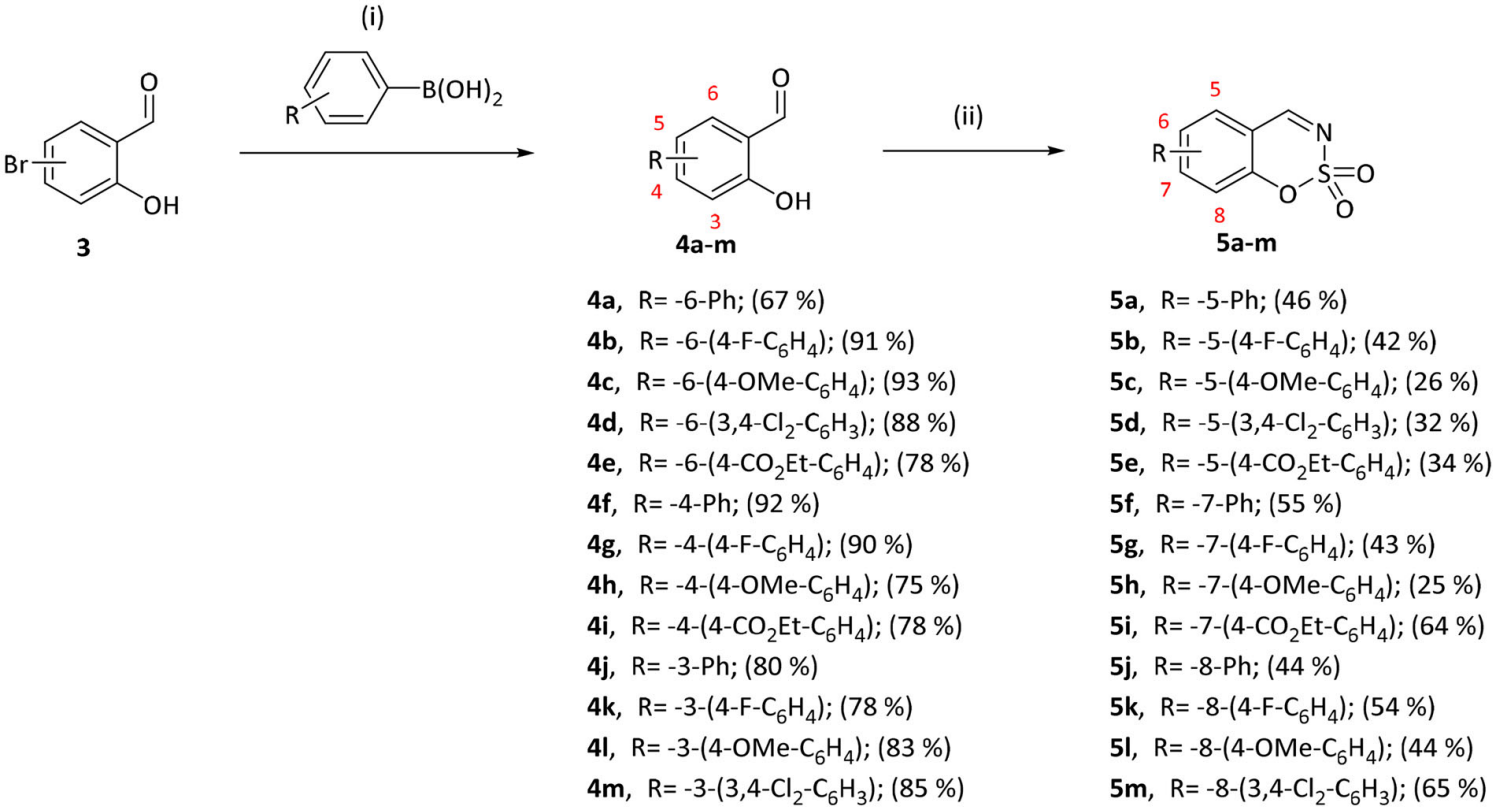
Reagents and conditions: (i) K_2_CO_3_, Pd(PPh_3_)_4_, toluene/H_2_O (5:1), 90 °C, 24–48 h; (ii) ClSO_2_NH_2_, DMA, RT, 24–48 h.

5-, 7-, and 8-aryl substituted benzo[1,2,3]oxathiazine-2,2-dioxides **5a–m** were obtained from aryl substituted 2-hydroxybenzaldehydes **4a–m** (Scheme 2) prepared from 3-, 4-, or 6-bromo-2-hydroxybenzaldehydes **3**. The first step was Suzuki-Miyaura coupling of aldehydes **3** with various boronic acids followed by cyclisation using sulfamoyl chloride. The yields of intermediates **4a–m** were from moderate to high, but the yields of the products **5a–m** were lower due to the loss during purification process. All inhibitors obtained exceeded 95% purity according HPLC analysis.

### Carbonic anhydrase inhibition

Twenty four derivatives of 1,2,3-benzoxathiazine 2,2-dioxide were screened for the inhibition of four human CA isoforms – the cytosolic off-targets hCA I and II as well as transmembrane, tumour-associated hCA IX and XII that are anticancer drug targets^18^^,^^47–52^.

Inhibition data of 1,2,3-benzoxathiazine 2,2-dioxides **2a–k** and **5a–m** (as well as acetazolamide AAZ as standard) against hCA I, II, IX, and XII after 15 min of incubation period of the enzyme and inhibitor solutions are presented in Table 1 ^32^.

Data obtained show that some derivatives of 1,2,3-benzoxathiazine 2,2-dioxide (**2** **b**, **2d**, **5a–e**) did not inhibit cytosolic isoform hCA I at all while other ones showed micromolar inhibitory activity in a quite wide range (0.36 − 46 µM).

hCA II, which is off-target in this case, was not inhibited only by two compounds, **5d** and **5e**. In case of 1,2,3-benzoxathiazine 2,2-dioxide derivatives **2a–k** with small substituents like Me, MeO, Br, F, inhibition constants in nanomolar range (39 − 169 nM) were obtained, except compound **2d** with Br in position 6 which has *K*_i_ = 8.3 µM. 5-Aryl substituted 1,2,3-benzoxathiazine 2,2-dioxide derivatives **5a–e** poorly inhibited hCA II or did not inhibit it at all, whereas 7- and 8-aryl derivatives showed nanomolar inhibitory activity towards hCA II.

The transmembrane isoform hCA IX was effectively inhibited by 1,2,3-benzoxathiazine 2,2-dioxides **2a–k** and **5f–m** with *K*_i_ in the range of 22 − 74 nM, although 5-aryl substituted derivatives showed micromolar inhibitory activity (**5a–c**, *K*_i_ = 4.1 − 10.9 µM), but **5d-e** did not show any inhibitory activity towards hCA IX.

Another transmembrane tumour-associated isoform hCA XII was also effectively inhibited by most of investigated compounds with *K*_i_ in the range of 5.7 − 186.4 nM. 5-Aryl derivatives **5a–d** showed lower inhibitory activity while compound **5e** did not exhibit any inhibitory properties towards hCA XII.

It may be seen that some of the new derivatives exhibit promising selectivity towards hCA IX/XII over hCA I, although none of the compounds are selective towards hCA IX/XII over both hCA I and II. The most promising tendence to selectivity towards hCA IX/XII over both hCA I and II is observed in case of compound **2d** which has Br in position 6.

## Conclusions

We report here a series of novel 1,2,3-benzoxathiazine-2,2-dioxide derivatives **2a–k** with small substituents (Me, MeO, F, Br) in 6, 7, or 8 position prepared by straightforward synthesis from corresponding 2-hydroxybenzaldehydes in their reaction with sulfamoyl chloride. A series of 5-, 7-, or 8-aryl substituted 1,2,3-benzoxathiazine-2,2-dioxides **5a–m** were obtained by two-step protocol from aryl substituted 2-hydroxybenzaldehydes in Suzuki-Miyaura reaction with selected boronic acids followed by cyclisation using sulfamoyl chloride.

The new derivatives were assayed as inhibitors of four hCA isoforms, the cytosolic hCA I and II, and the transmembrane, tumour-associated hCA IX and XII.

1,2,3-Benzoxathiazine-2,2-dioxides generally do not inhibit or show low inhibitory activity towards cytosolic widespread hCA I.

Ubiquitous hCA II was inhibited by the new derivatives in nanomolar and micromolar range whereas two 5-aryl derivatives did not show any hCA II inhibitory activity.

Transmembrane isoforms hCA IX and XII were inhibited in nanomolar range by most of 1,2,3-benzoxathiazine-2,2-dioxides although 5-aryl derivatives showed lower inhibitory activity or did not inhibit these two isoenzymes.

Some 1,2,3-benzoxathiazine-2,2-dioxides exhibit promising selectivity towards hCA IX/XII over hCA I, although none of the compounds are selective towards hCA IX/XII over both hCA I and II.

## Supplementary Material

Supplemental Material
