# Supplementary material for: 1,2,3-Benzoxathiazine-2,2-dioxides – effective inhibitors of human carbonic anhydrases
Source: J Enzyme Inhib Med Chem. 2022 Nov 13;38(1):225–38. doi: 10.1080/14756366.2022.2142787 (PMC9673787; doi:10.1080/14756366.2022.2142787)
Supplement: Supplemental Material [file IENZ_A_2142787_SM7046.pdf]

Supporting Information

1,2,3-Benzoxathiazine-2,2-dioxides – effective inhibitors of human carbonic anhydrases

Jekaterīna Ivanova,[1] Morteza Abdoli,[2] Alessio Nocentini,[3] Raivis Žalubovskis, *,[1,2] Claudiu T. Supuran[3]

[1] Latvian Institute of Organic Synthesis, Riga, Latvia;

[2] Institute of Technology of Organic Chemistry, Faculty of Materials Science and Applied Chemistry, Riga Technical University, Riga, Latvia;

[3] Neurofarba Department, Università degli Studi di Firenze, Florence, Italy;

* CONTACT Raivis Žalubovskis raivis@osi.lv Latvian Institute of Organic Synthesis, 21 Aizkraukles Str, Riga, LV-1006, Latvia; Claudiu T. Supuran claudiu.supuran@unifi.it Dipartimento Neurofarba, Sezione di Scienze Farmaceutiche e Nutraceutiche, Università degli Studi di Firenze, Sesto Fiorentino, Florence, Italy

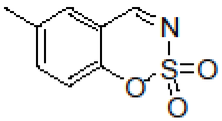

2a

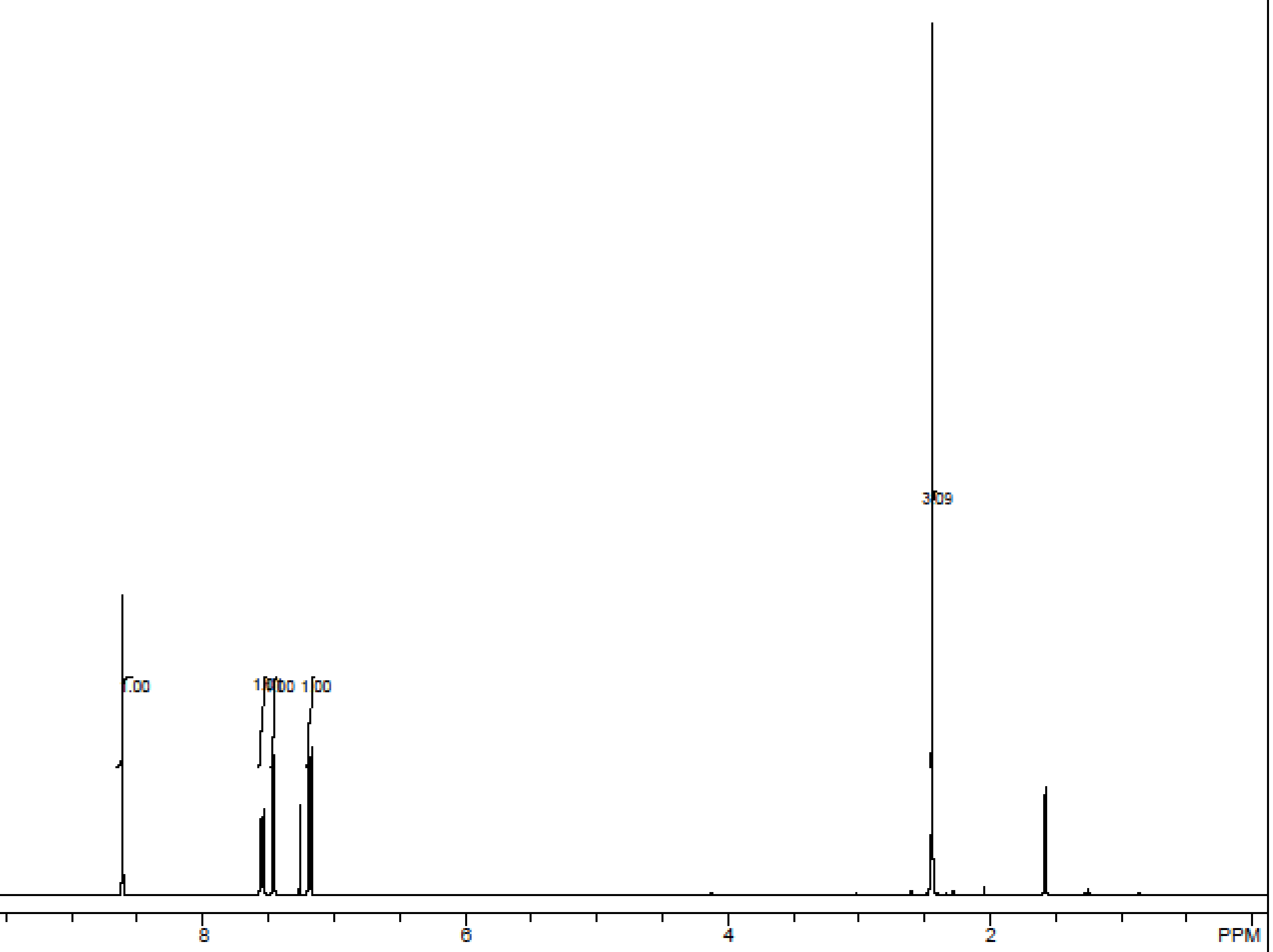

Avance, CDCl3 USER: nmrsu – DATE: Thu Jul 30 08:58:23 2020

F1: 399.956	F2: 1.000	SW1: 7813		OF1: 2460.1		PTS1d: 65536	
EX: zg30		PW: 12.0 us	PD: 1.0 sec	NA: 16	LB: 0.0	Nuts - $pdata	

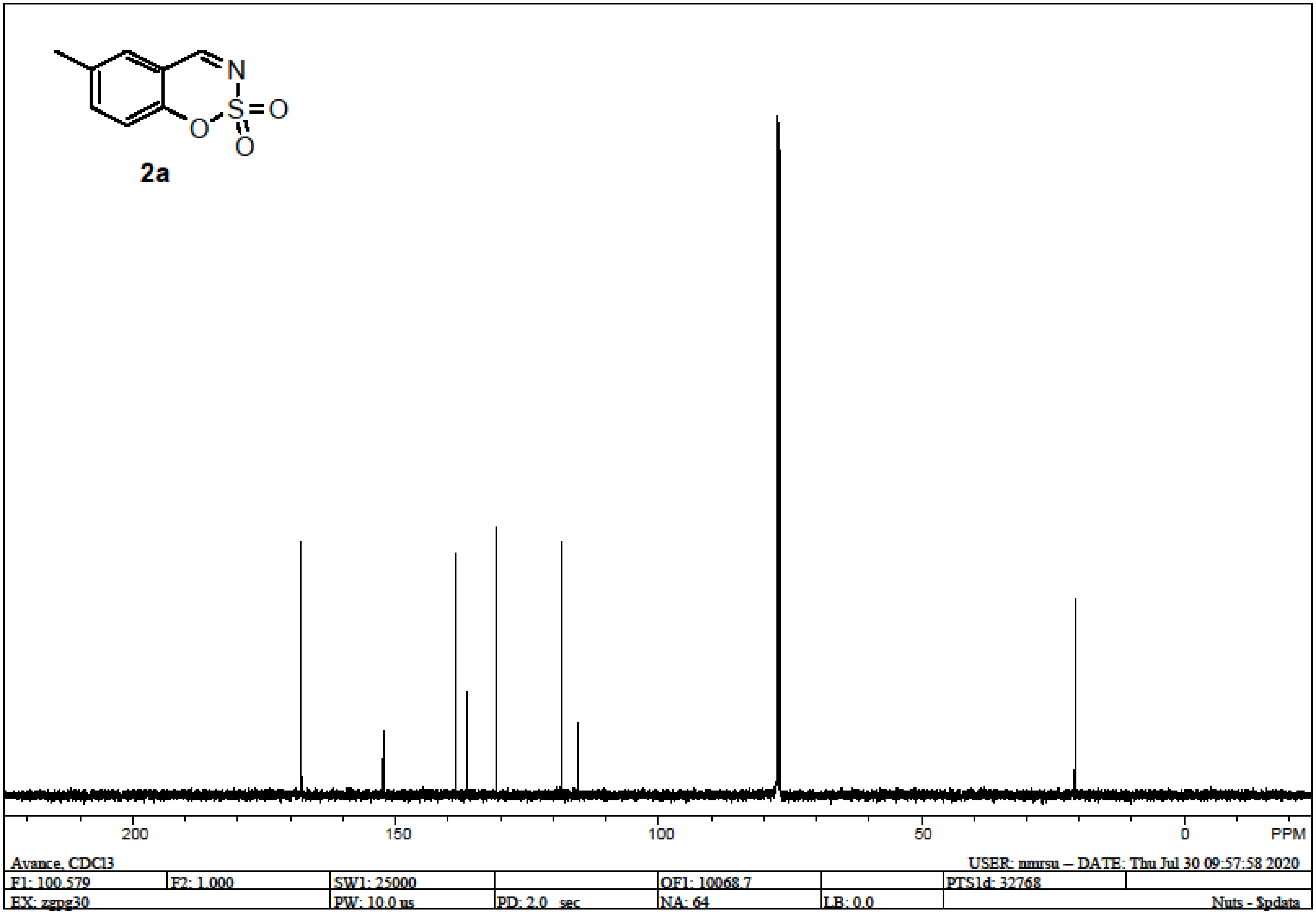
N
S
O
O
O
2a
200
150
100
50
0
PPM
Avance, CDCl3
USER: nmrsu -- DATE: Thu Jul 30 09:57:58 2020
F1: 100.579
F2: 1.000
SW1: 25000
OF1: 10068.7
PTS1d: 32768
EX: zgpg30
PW: 10.0 us
PD: 2.0 sec
NA: 64
LB: 0.0
Nuts - $pdata

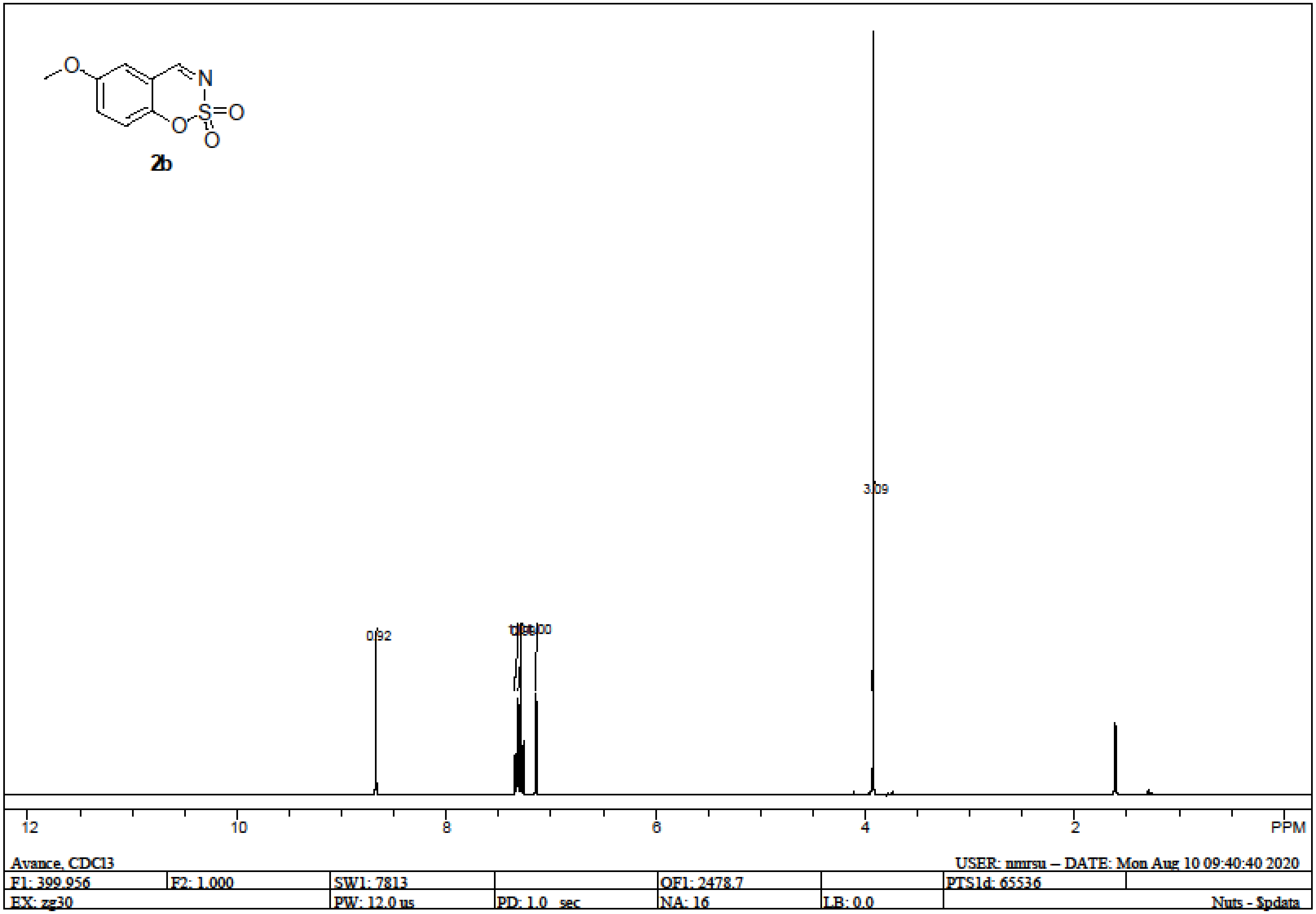

2b
0.92
3.09
12
10
8
6
4
2
PPM
Avance, CDCl3
USER: nmrsu -- DATE: Mon Aug 10 09:40:40 2020
F1: 399.956
F2: 1.000
SW1: 7813
OF1: 2478.7
PTS1d: 65536
EX: zg30
PW: 12.0 us
PD: 1.0 sec
NA: 16
LB: 0.0
Nuts - $pdata

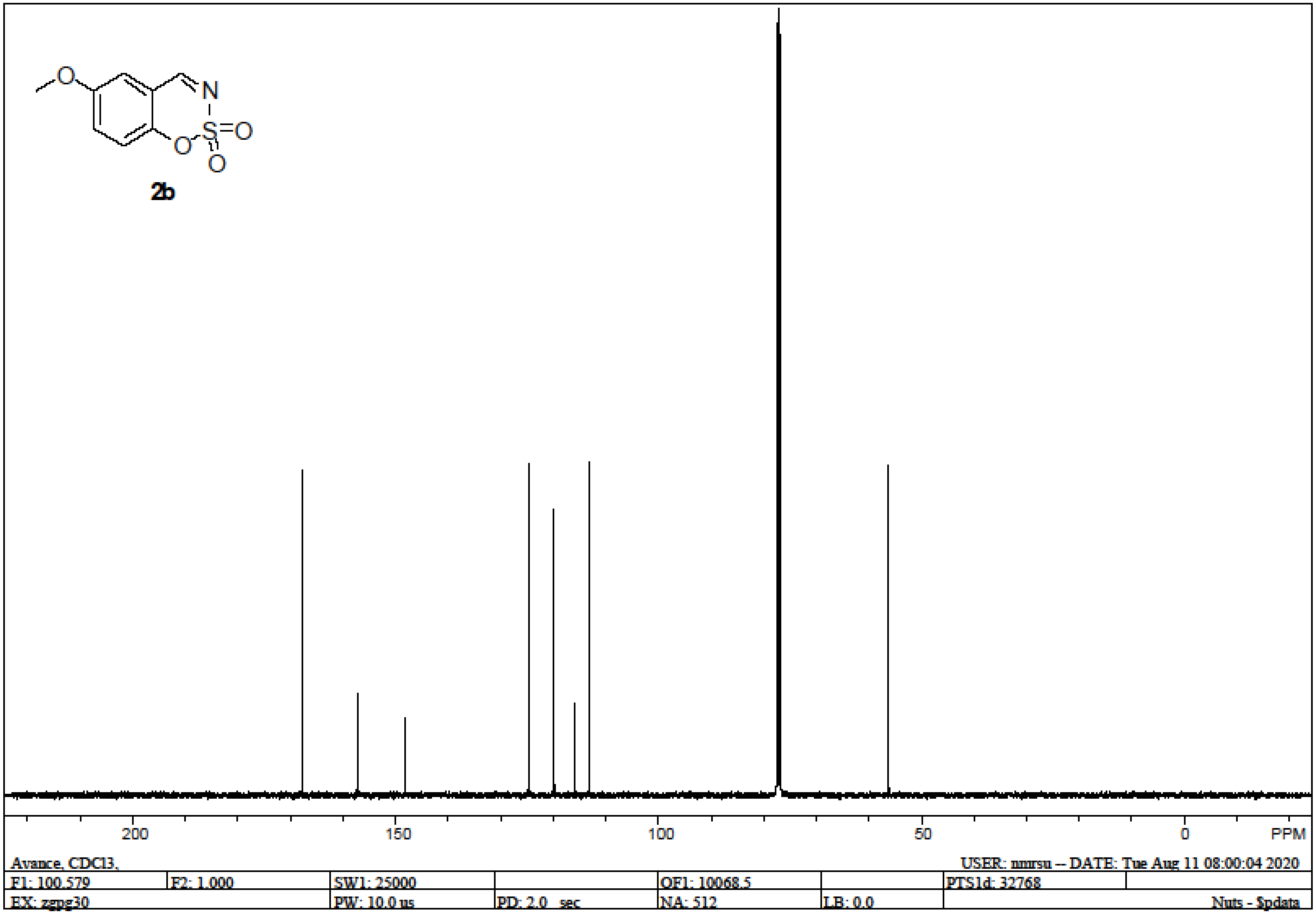

2b
200
150
100
50
0
PPM
Avance, CDCl3,
USER: nmrsu -- DATE: Tue Aug 11 08:00:04 2020
F1: 100.579
F2: 1.000
SW1: 25000
OF1: 10068.5
PTS1d: 32768
EX: zgpg30
PW: 10.0 us
PD: 2.0 sec
NA: 512
LB: 0.0
Nuts - $pdata

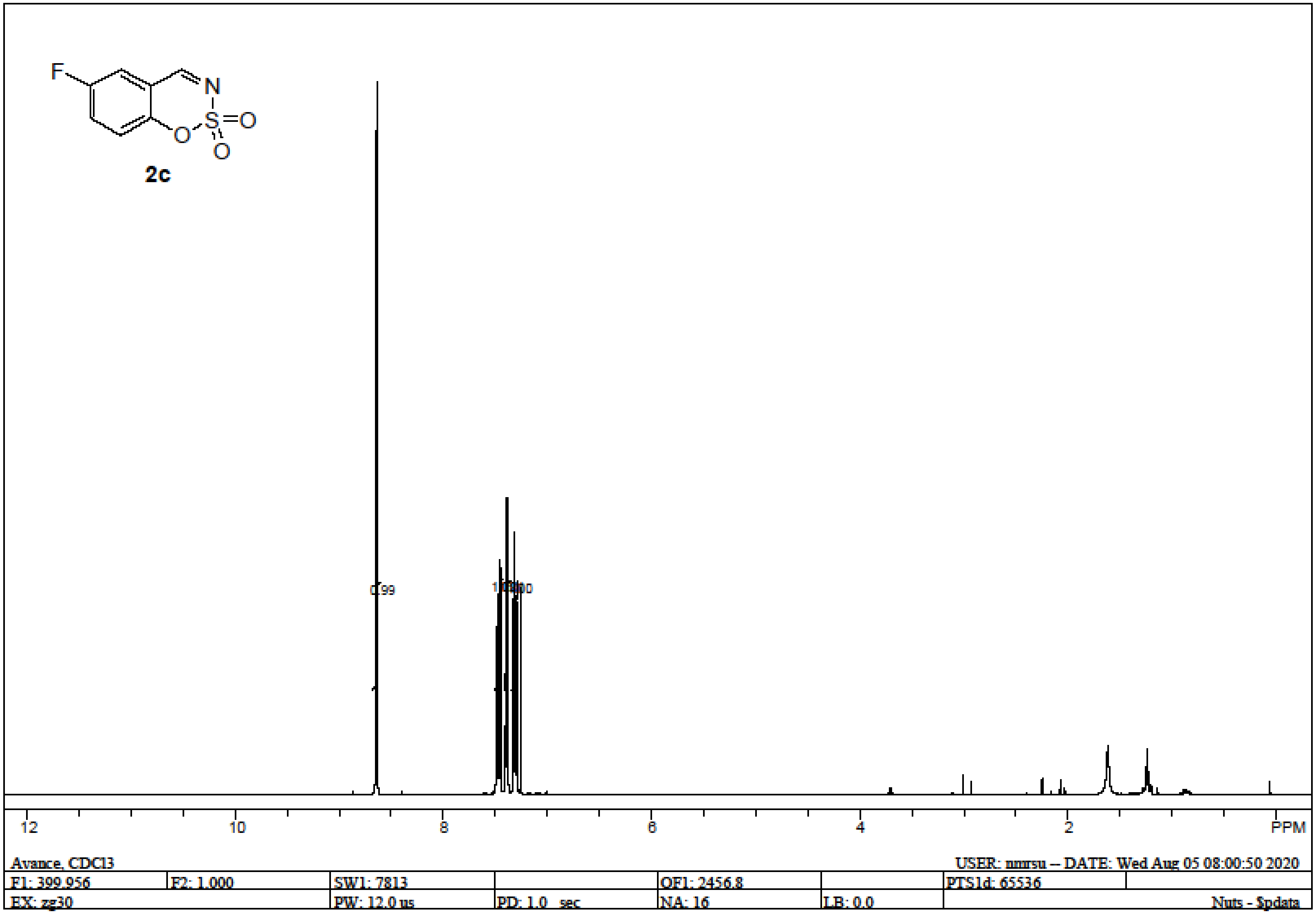
F
N
S
O
O
O
2c
0.99
1.00
12
10
8
6
4
2
PPM
Avance, CDCl3
USER: nmrsu -- DATE: Wed Aug 05 08:00:50 2020
F1: 399.956
F2: 1.000
SW1: 7813
OF1: 2456.8
PTS1d: 65536
EX: zg30
PW: 12.0 us
PD: 1.0 sec
NA: 16
LB: 0.0
Nuts - $pdata

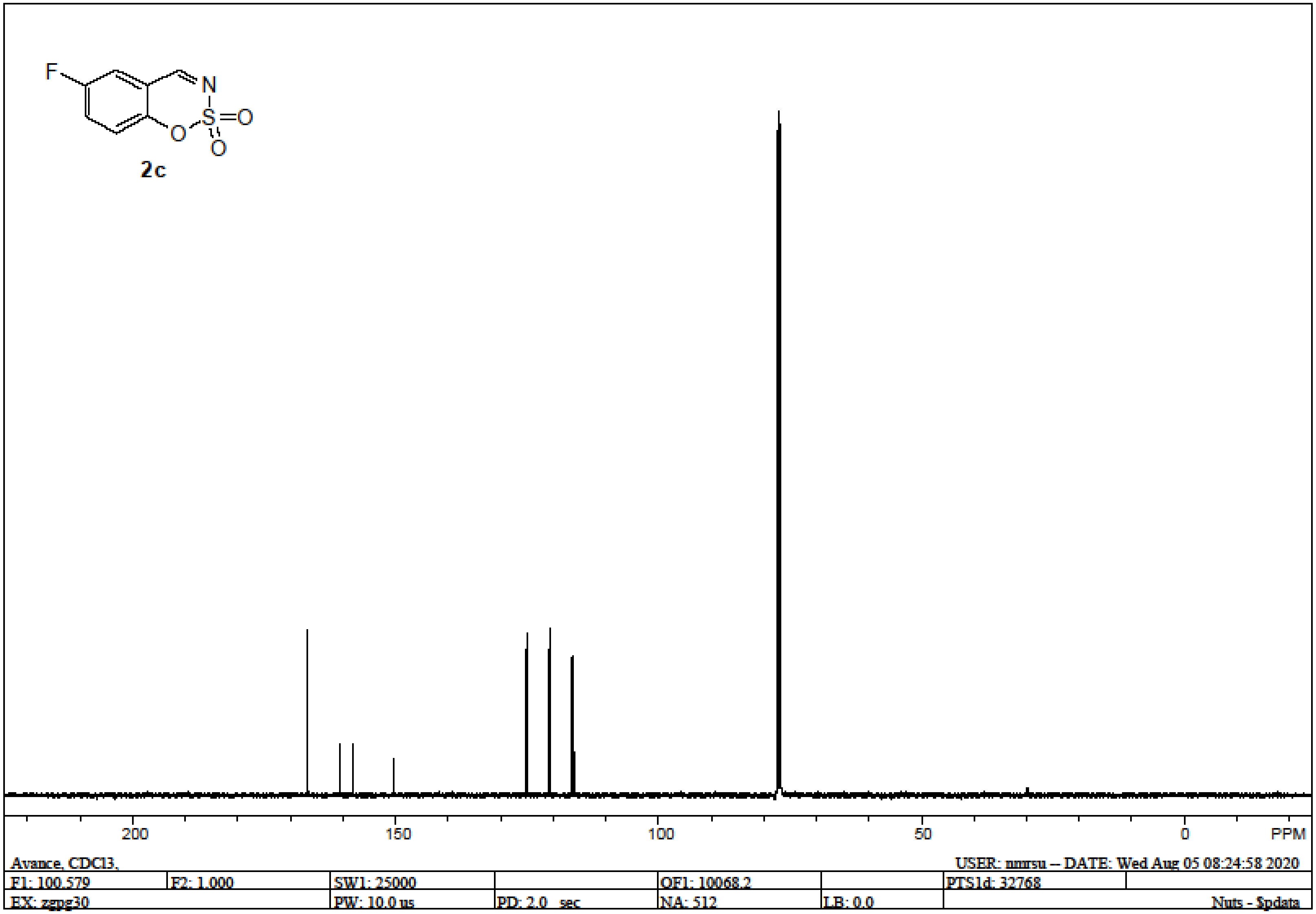

F
N
S
O
O
O
2c
200
150
100
50
0
PPM
Avance, CDCl3,
USER: nmrsu -- DATE: Wed Aug 05 08:24:58 2020
F1: 100.579
F2: 1.000
SW1: 25000
OF1: 10068.2
PTS1d: 32768
EX: zgpg30
PW: 10.0 us
PD: 2.0 sec
NA: 512
LB: 0.0
Nuts - $pdata

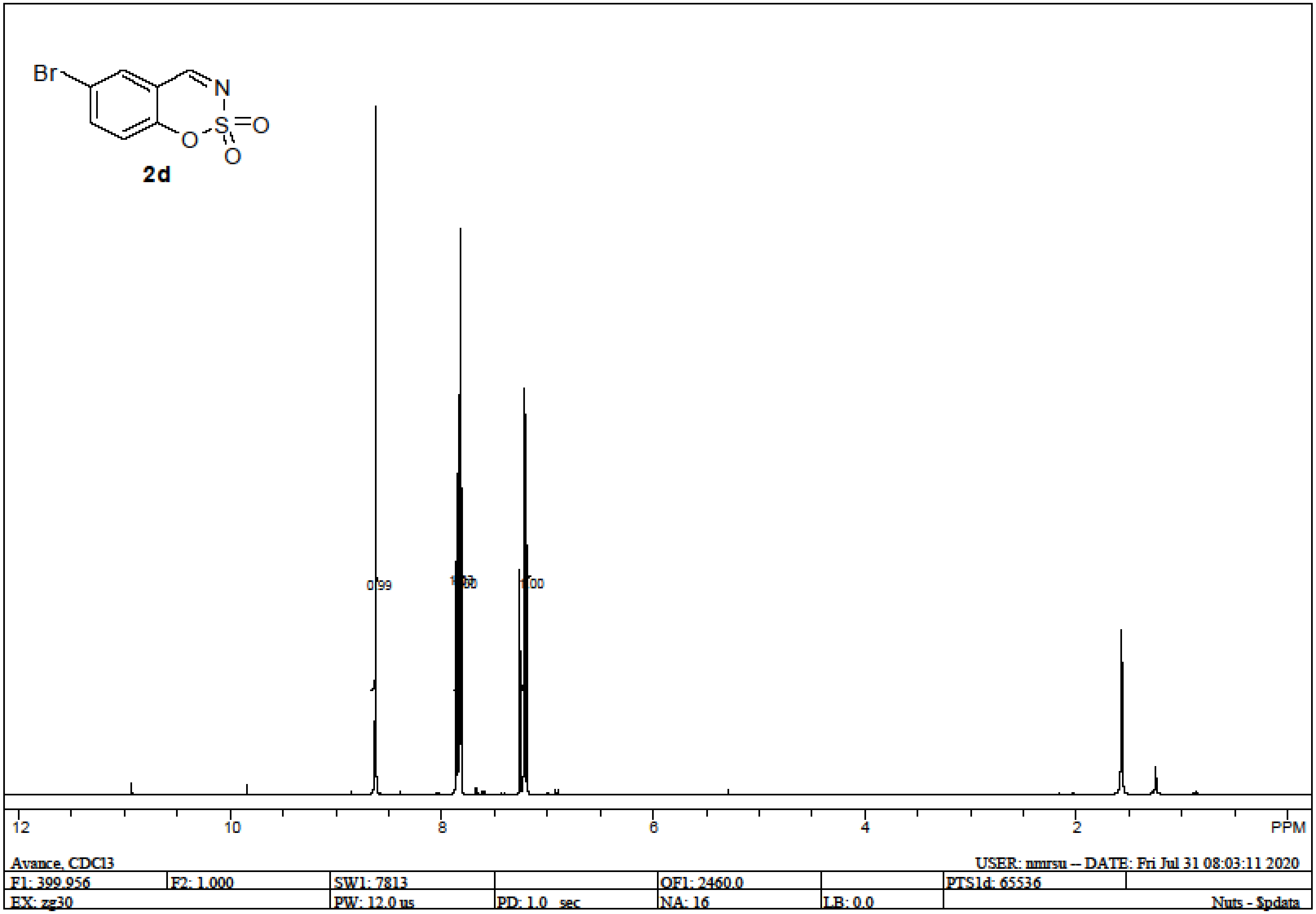
Br
N
S
O
O
O
2d
0.99
1.00
12
10
8
6
4
2
PPM
Avance, CDCl3
USER: nmrsu -- DATE: Fri Jul 31 08:03:11 2020
F1: 399.956
F2: 1.000
SW1: 7813
OF1: 2460.0
PTS1d: 65536
EX: zg30
PW: 12.0 us
PD: 1.0 sec
NA: 16
LB: 0.0
Nuts - $pdata

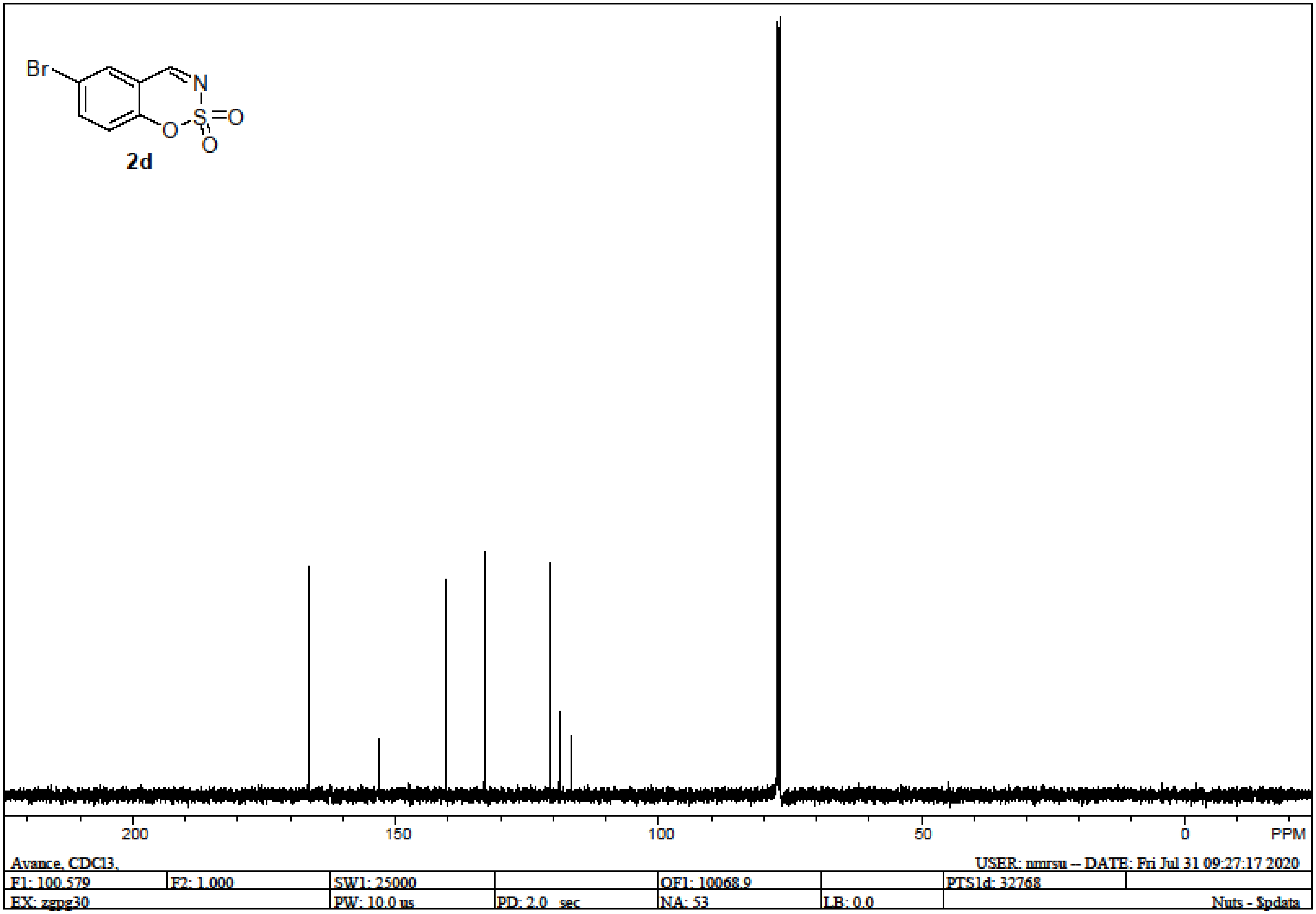
Br
N
S
O
O
O
2d
200
150
100
50
0
PPM
Avance, CDCl3,
USER: nmrsu -- DATE: Fri Jul 31 09:27:17 2020
F1: 100.579
F2: 1.000
SW1: 25000
OF1: 10068.9
PTS1d: 32768
EX: zgpg30
PW: 10.0 us
PD: 2.0 sec
NA: 53
LB: 0.0
Nuts - $pdata

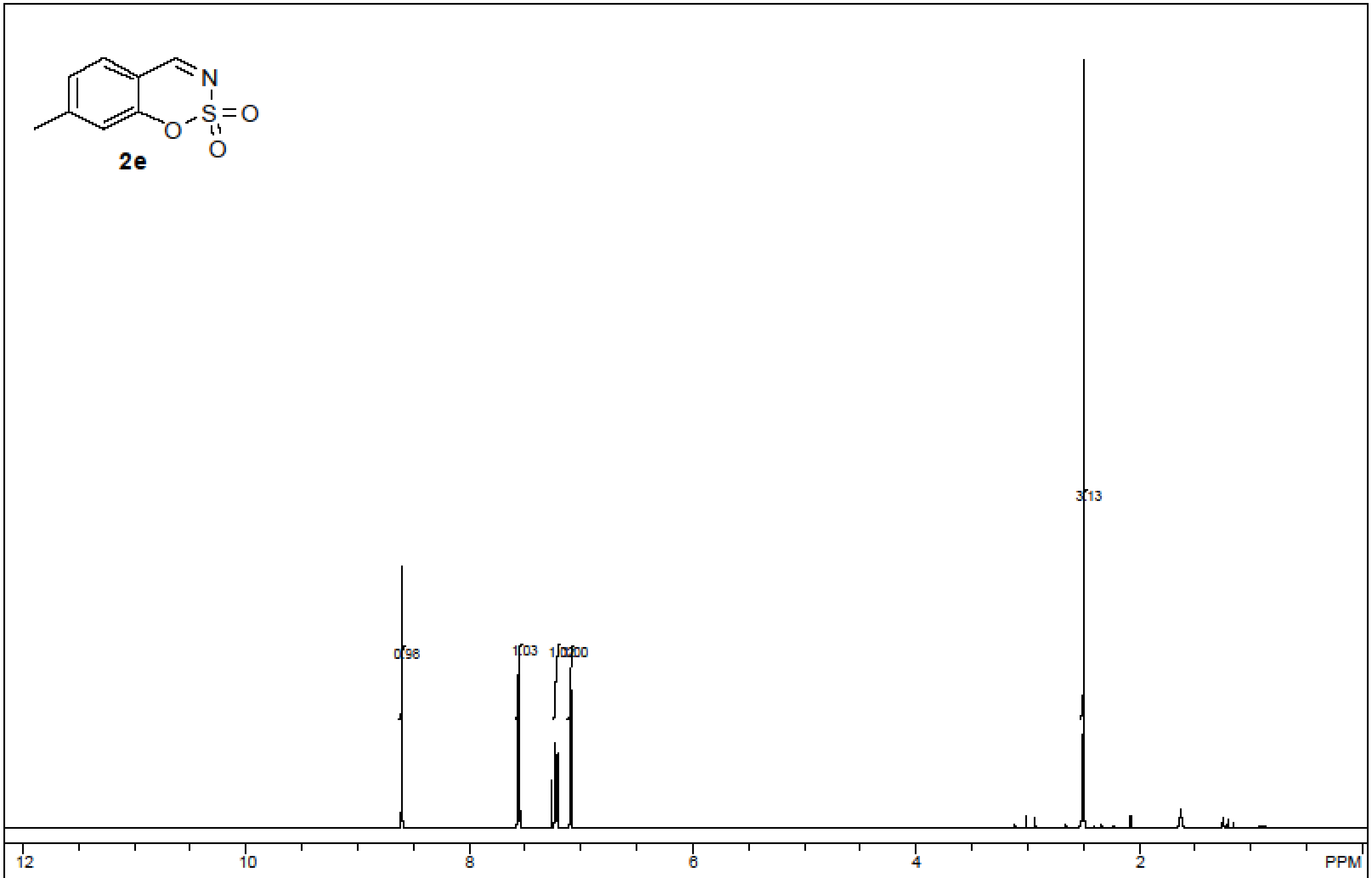

Avance, CDCl3 USER: nmrsu -- DATE: Tue Aug 04 10:38:29 2020

F1: 399.956	F2: 1.000	SW1: 7813		OF1: 2460.1		PTS1d: 65536	
EX: zg30		PW: 12.0 us	PD: 1.0 sec	NA: 16	LB: 0.0	Nuts - $pdata	

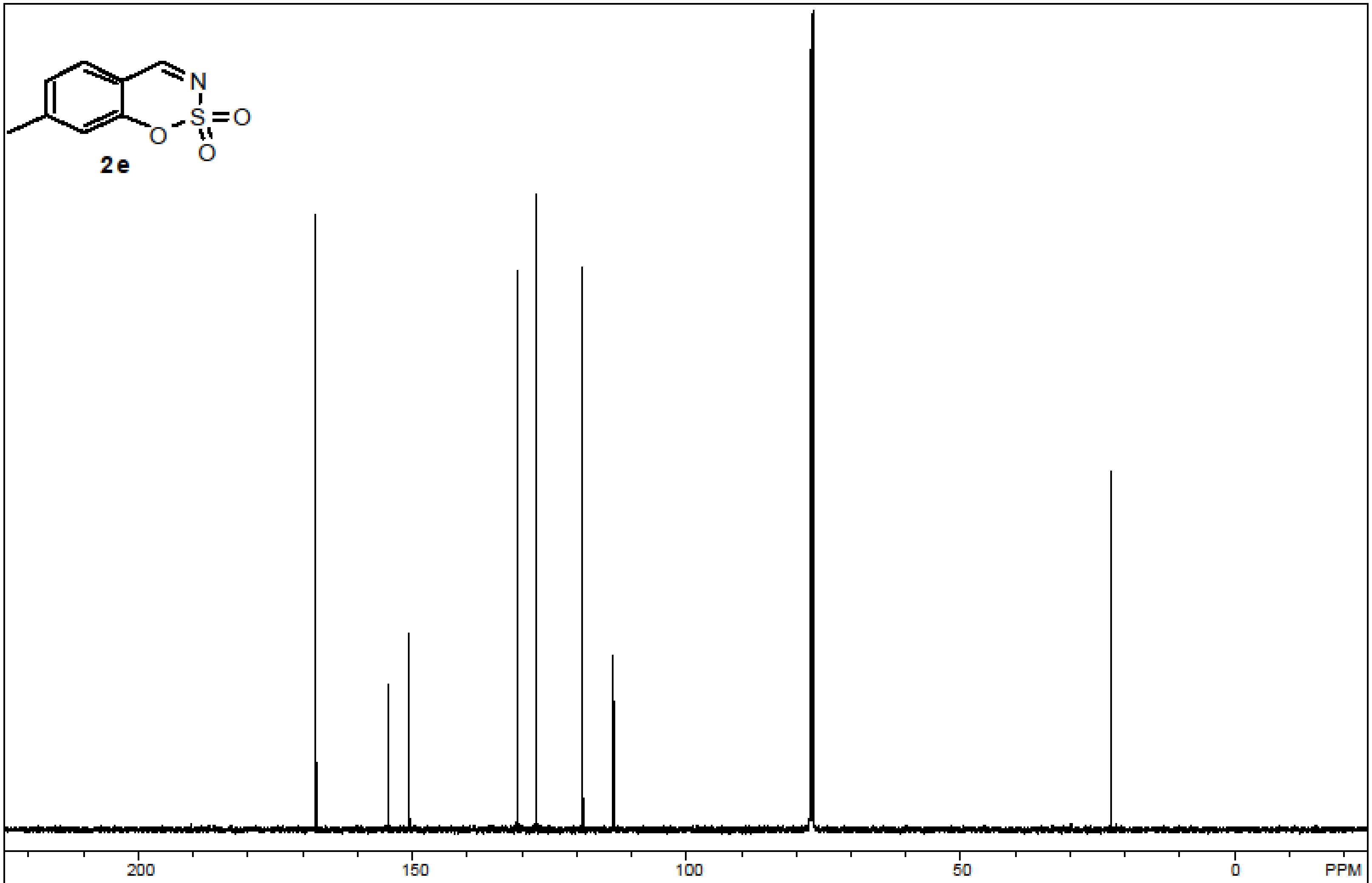

Avance, CDCl3 USER: nmrsu -- DATE: Tue Aug 04 11:02:37 2020

F1: 100.579	F2: 1.000	SW1: 25000		OF1: 10066.6		PTS1d: 32768	
EX: zgpg30		PW: 10.0 us	PD: 2.0 sec	NA: 512	LB: 0.0		Nuts - $pdata

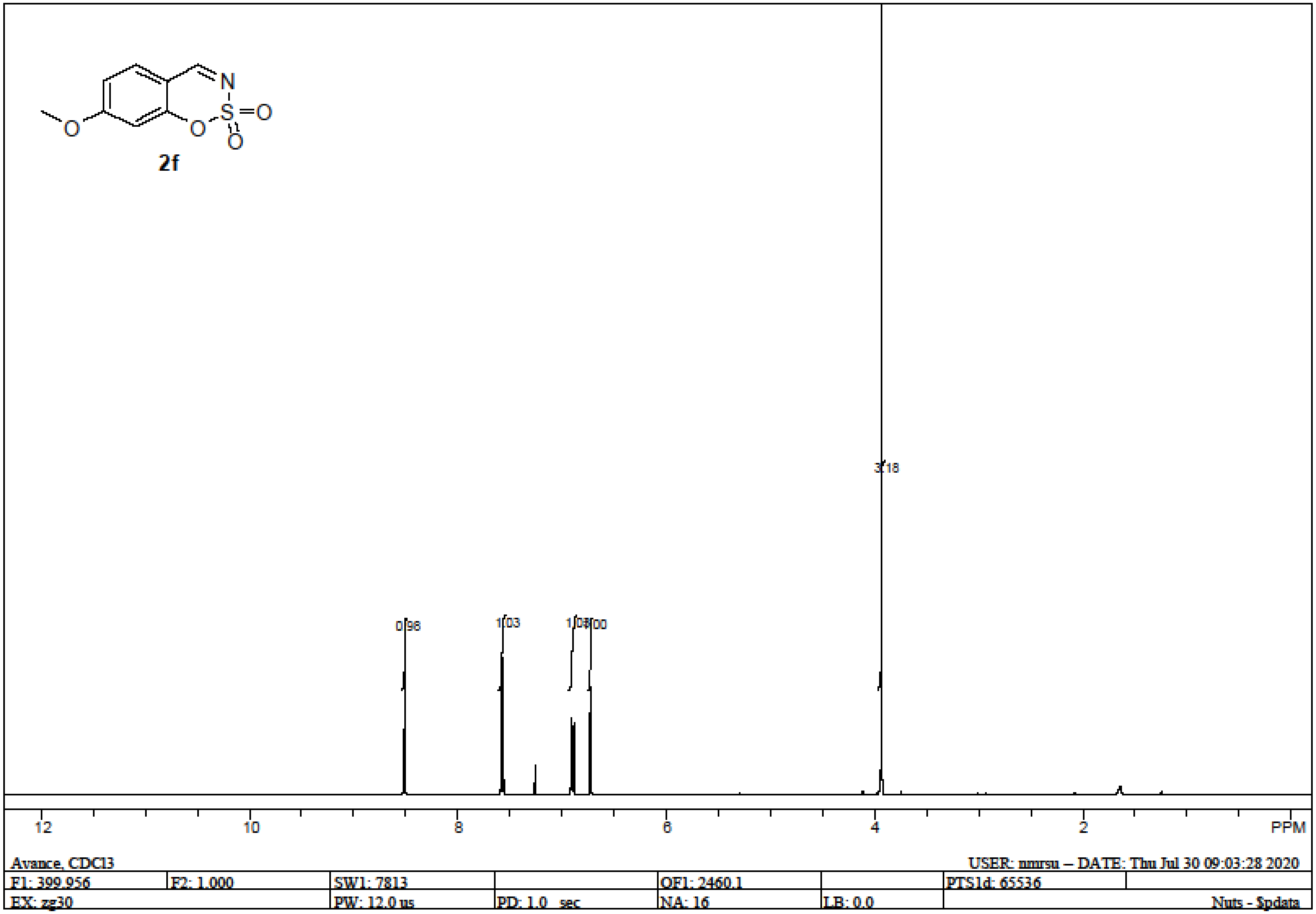

2f
0.98
1.03
1.08
1.00
3.18
12
10
8
6
4
2
PPM
Avance, CDCl3
USER: nmrsu -- DATE: Thu Jul 30 09:03:28 2020
F1: 399.956
F2: 1.000
SW1: 7813
OF1: 2460.1
PTS1d: 65536
EX: zg30
PW: 12.0 us
PD: 1.0 sec
NA: 16
LB: 0.0
Nuts - $pdata

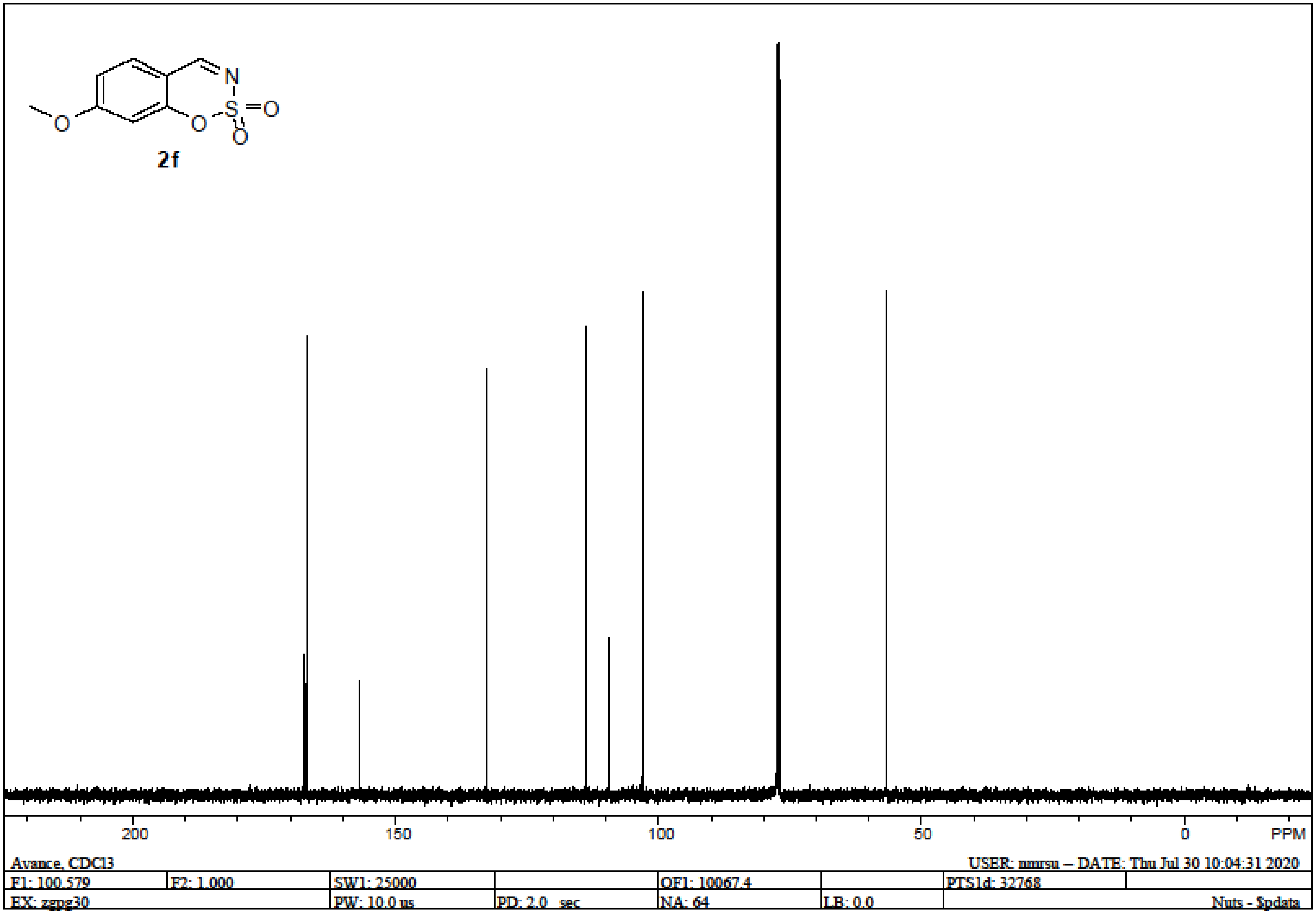
2f
200
150
100
50
0
PPM
Avance, CDCl3
USER: nmrsu – DATE: Thu Jul 30 10:04:31 2020
F1: 100.579
F2: 1.000
SW1: 25000
OF1: 10067.4
PTS1d: 32768
EX: zgpg30
PW: 10.0 us
PD: 2.0 sec
NA: 64
LB: 0.0
Nuts - $pdata

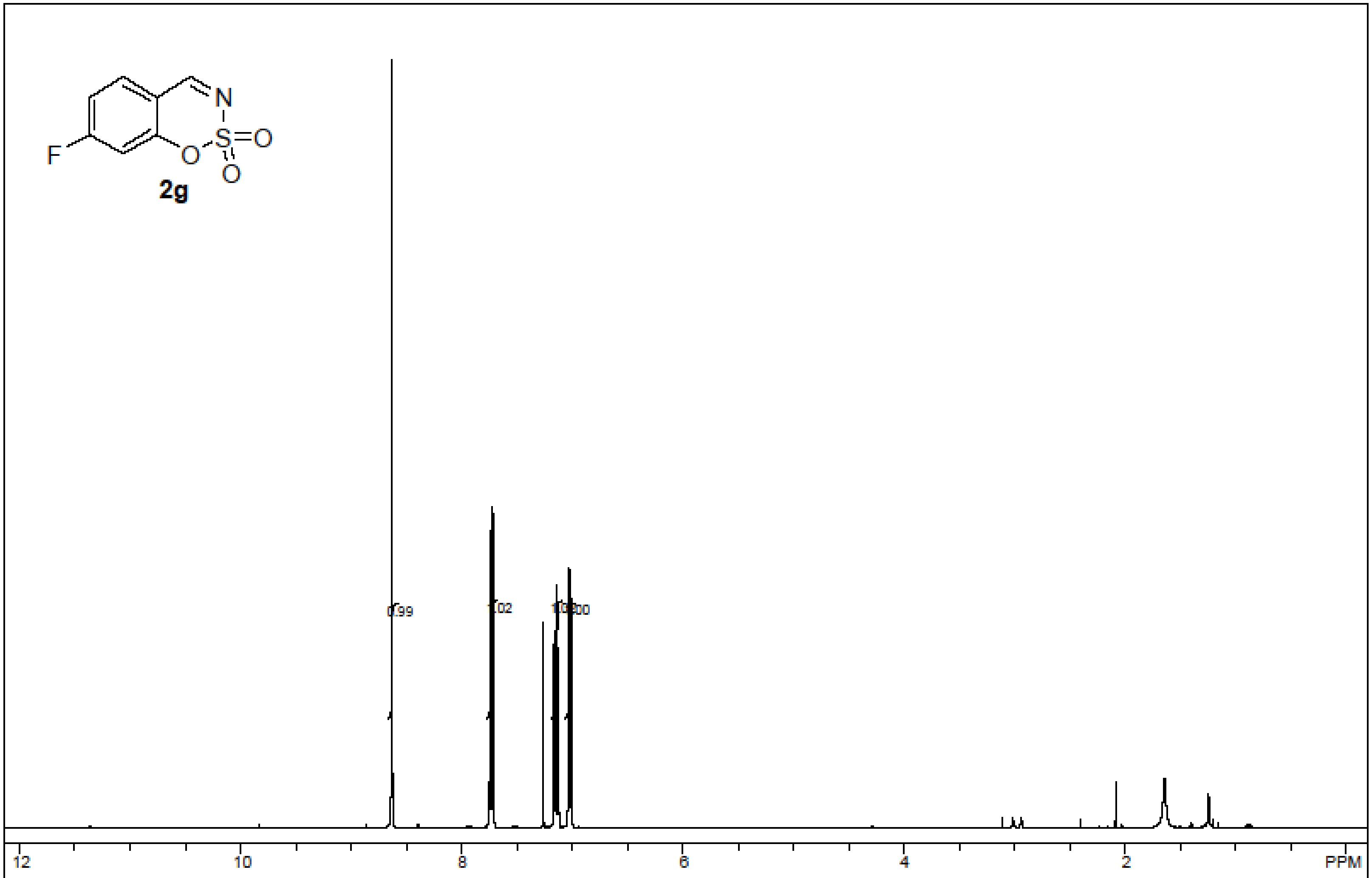

Avance, CDCl3, USER: nmrsu -- DATE: Fri Jul 31 08:07:28 2020

F1: 399.956	F2: 1.000	SW1: 7813		OF1: 2460.0		PTS1d: 65536	
EX: zg30		PW: 12.0 us	PD: 1.0 sec	NA: 16	LB: 0.0	Nuts - $pdata	

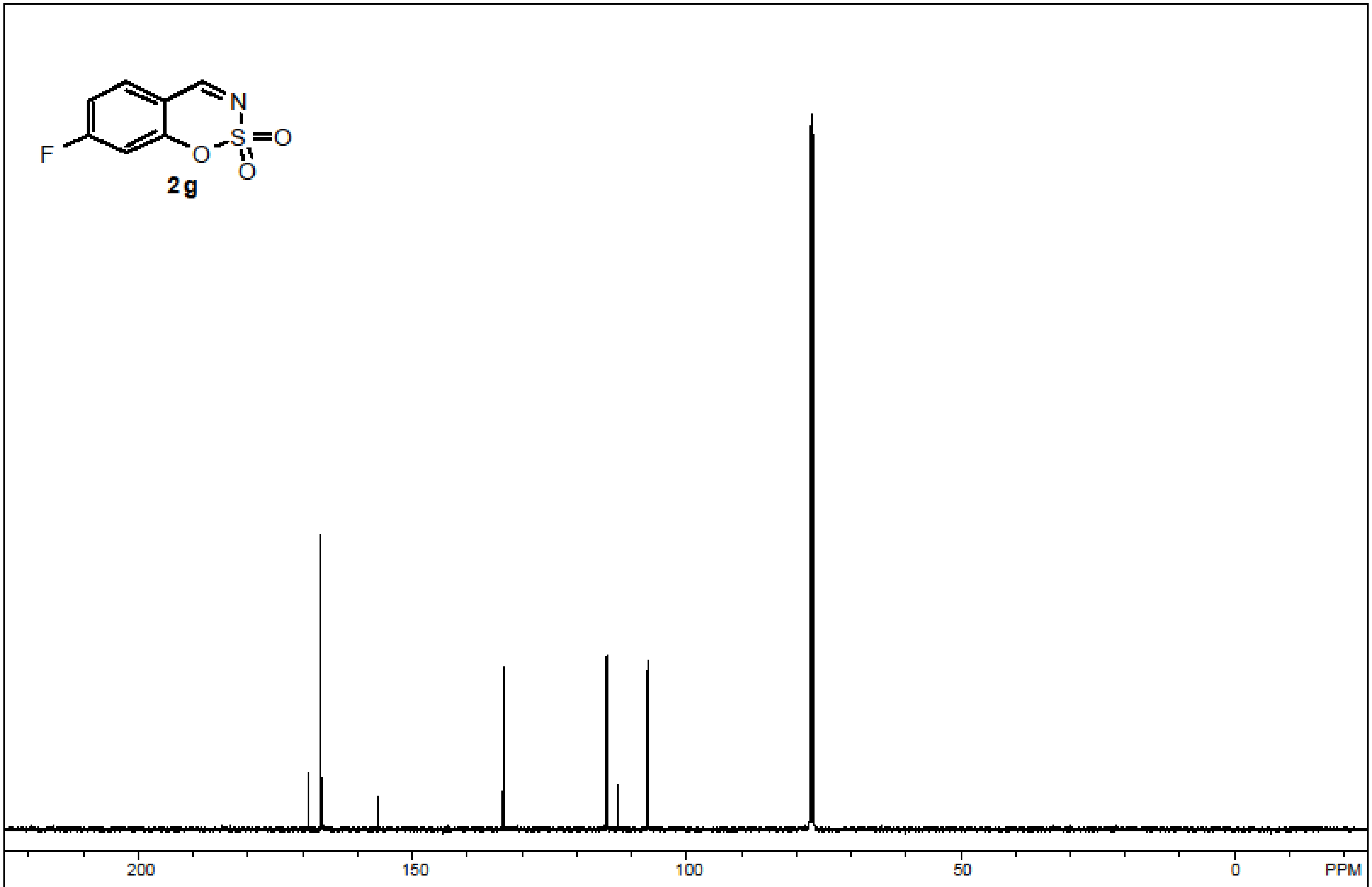

Avance, CDCl3,

USER: nmrsu -- DATE: Fri Jul 31 09:21:28 2020

F1: 100.579	F2: 1.000	SW1: 25000		OF1: 10068.4		PTS1d: 32768	
EX: zgpg30		PW: 10.0 us	PD: 2.0 sec	NA: 512	LB: 0.0	Nuts - $pdata	

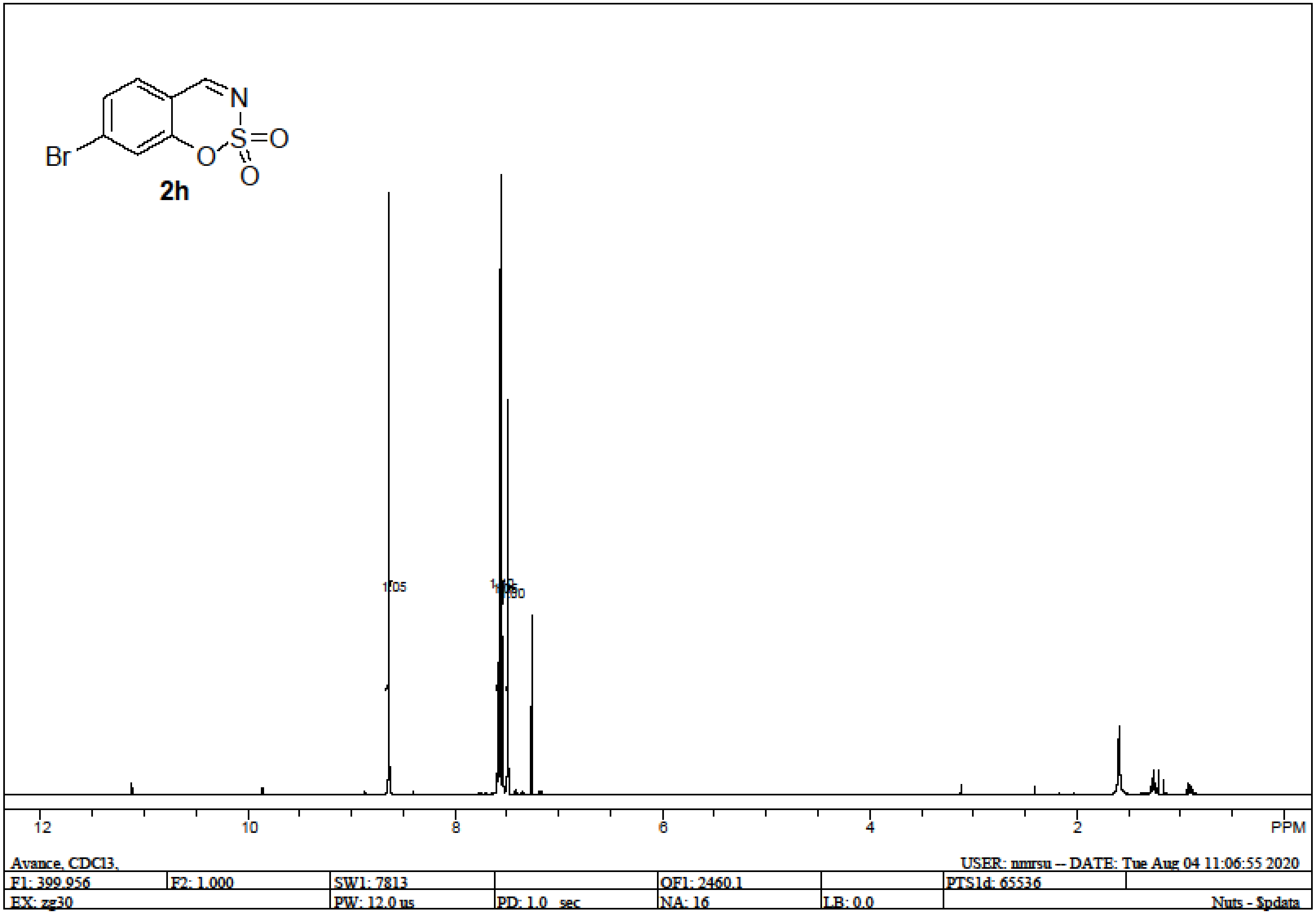
Br
N
S
O
O
O
2h
1.05
1.00
12
10
8
6
4
2
PPM
Avance, CDCl3,
USER: nmrsu -- DATE: Tue Aug 04 11:06:55 2020
F1: 399.956
F2: 1.000
SW1: 7813
OF1: 2460.1
PTS1d: 65536
EX: zg30
PW: 12.0 us
PD: 1.0 sec
NA: 16
LB: 0.0
Nuts - $pdata

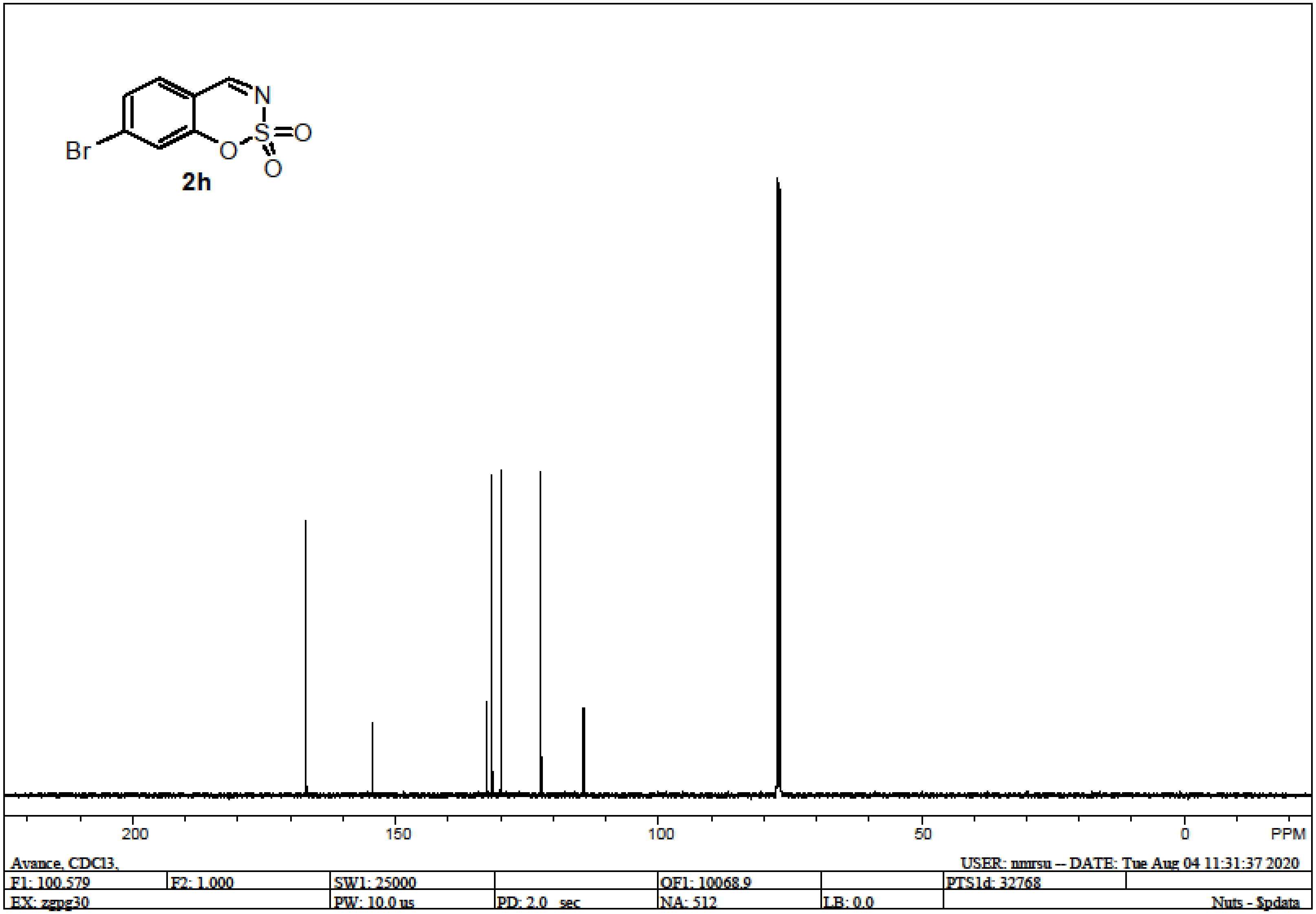

Br
N
S
O
O
O
2h
200
150
100
50
0
PPM
Avance, CDCl3,
USER: nmrsu -- DATE: Tue Aug 04 11:31:37 2020
F1: 100.579
F2: 1.000
SW1: 25000
OF1: 10068.9
PTS1d: 32768
EX: zgpg30
PW: 10.0 us
PD: 2.0 sec
NA: 512
LB: 0.0
Nuts - $pdata

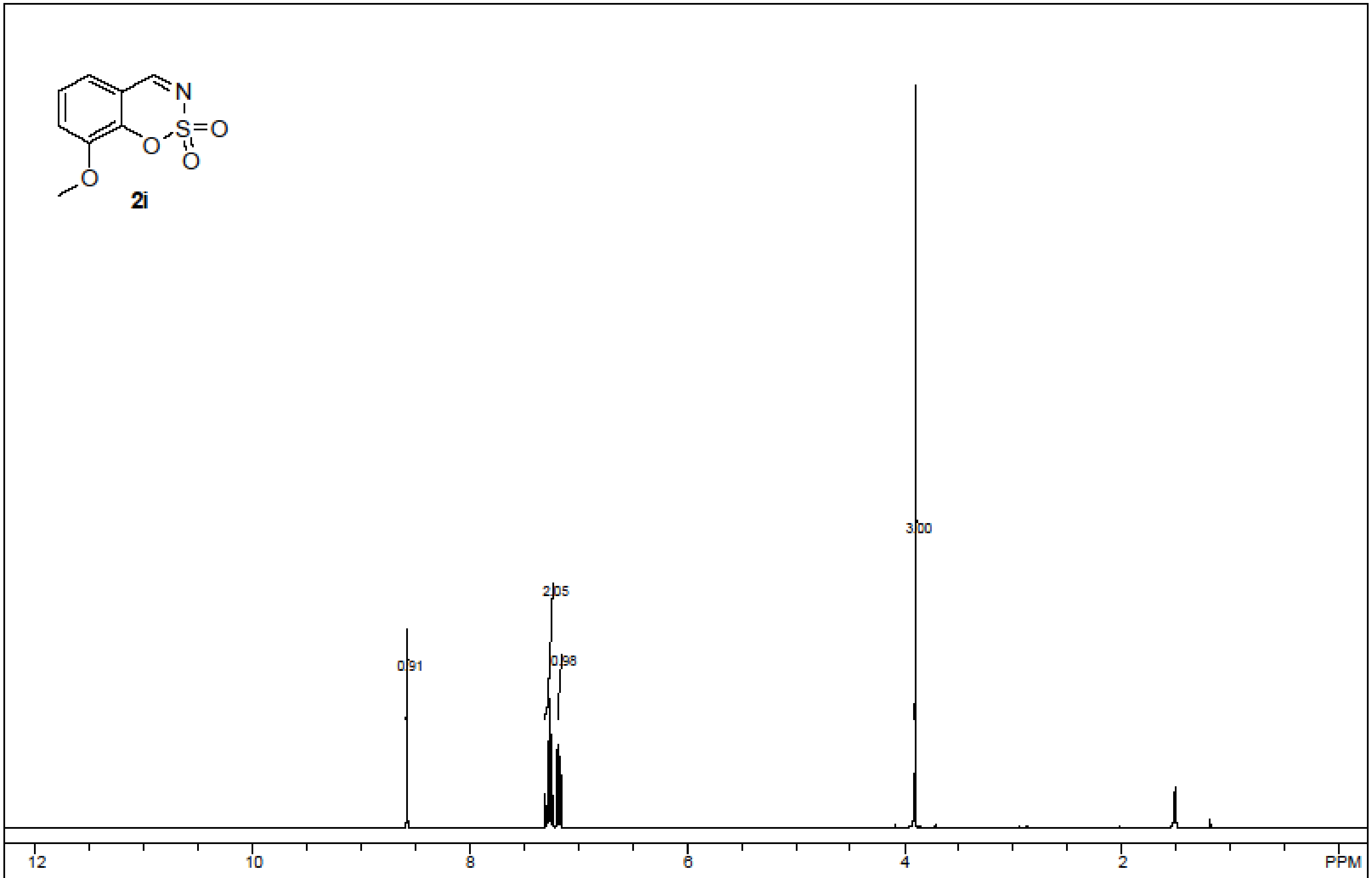

Avance, CDCl3, USER: nmrsu -- DATE: Mon Jun 29 09:07:43 2020

F1: 399.956	F2: 1.000	SW1: 7813		OF1: 2433.0		PTS1d: 65536	
EX: zg30		PW: 12.0 us	PD: 1.0 sec	NA: 16	LB: 0.0		Nuts - $pdata

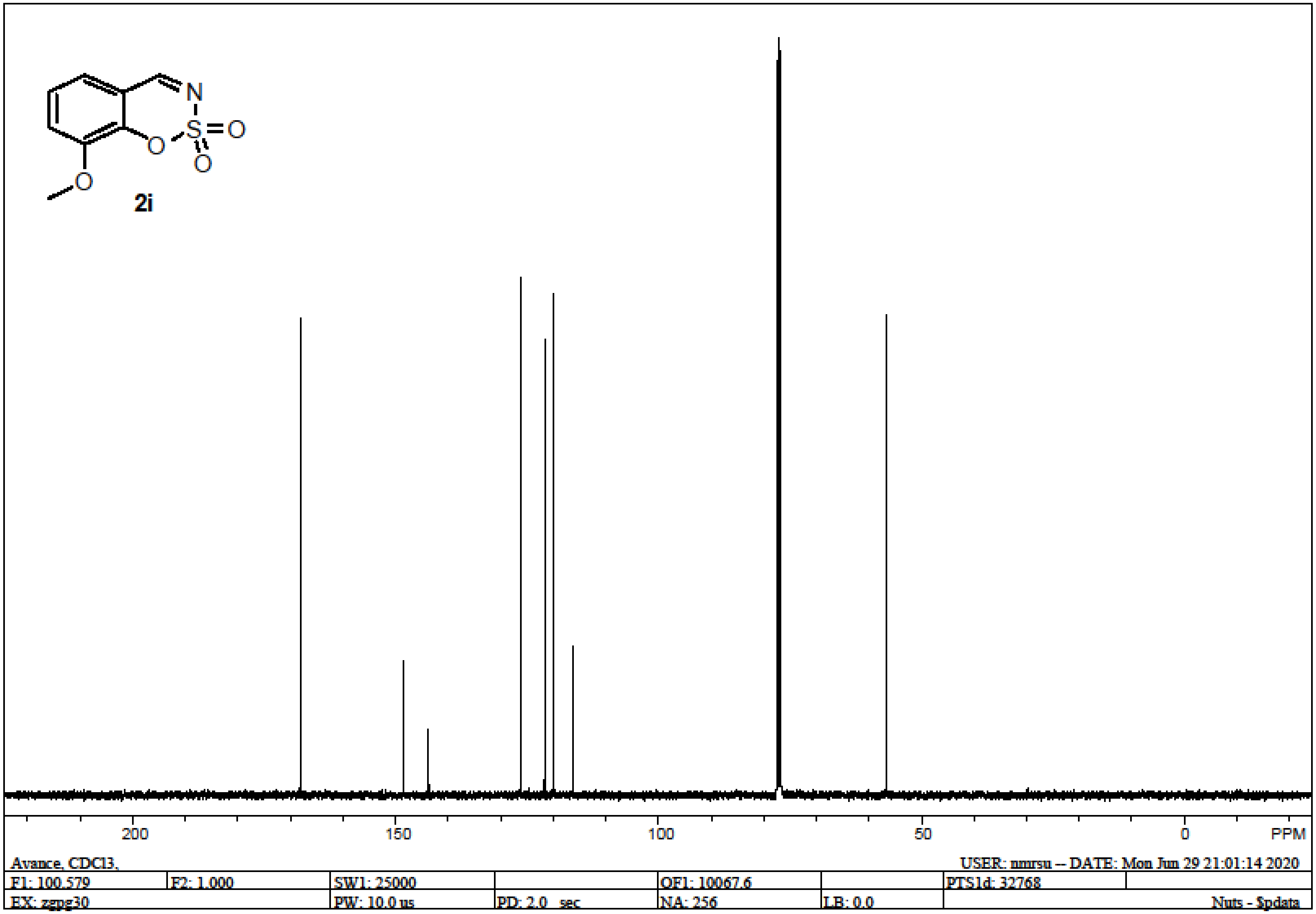
2i
200
150
100
50
0
PPM
Avance, CDCl3,
USER: nmrsu -- DATE: Mon Jun 29 21:01:14 2020
F1: 100.579
F2: 1.000
SW1: 25000
OF1: 10067.6
PTS1d: 32768
EX: zgpg30
PW: 10.0 us
PD: 2.0 sec
NA: 256
LB: 0.0
Nuts - $pdata

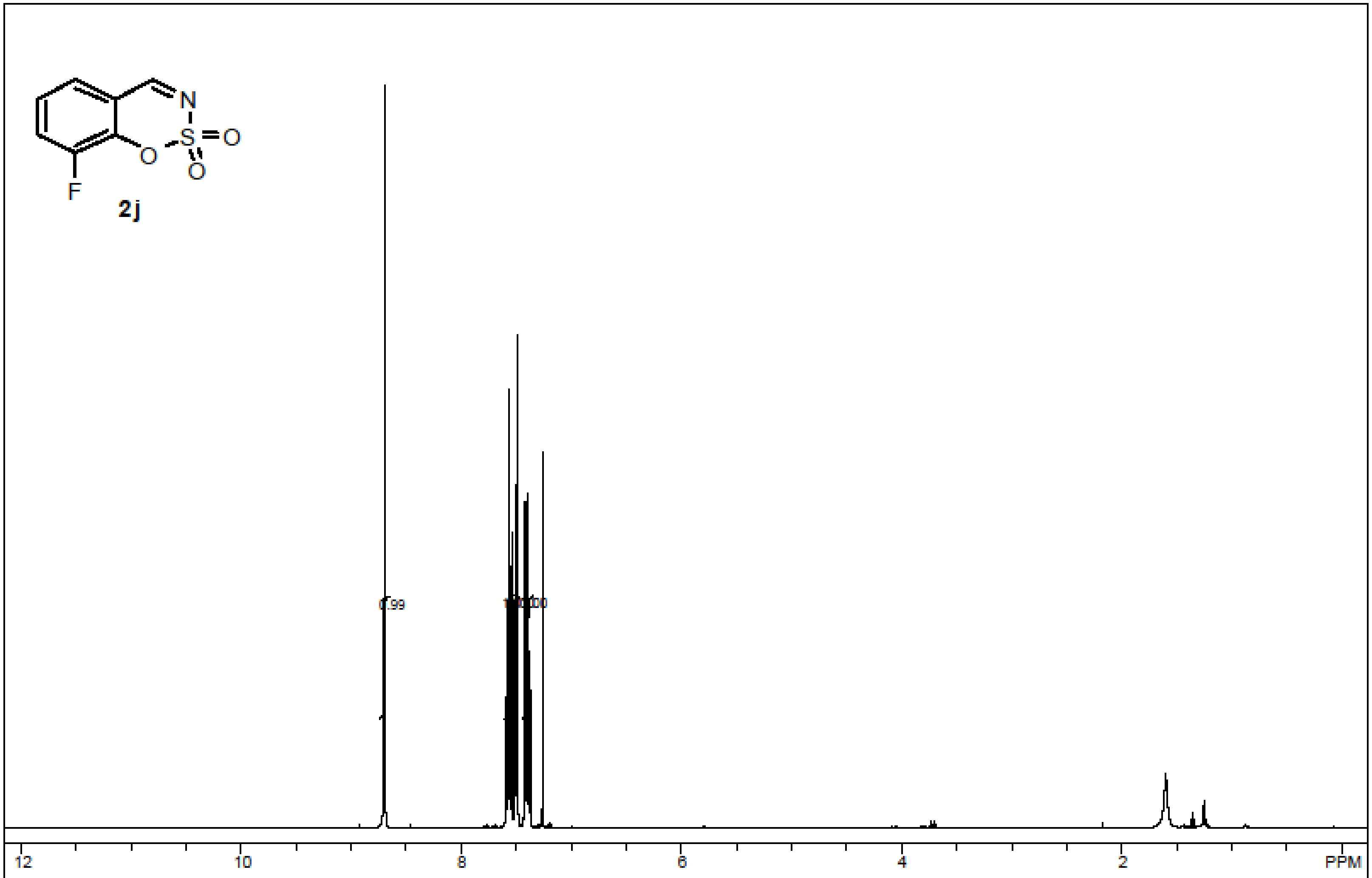

Avance, CDCl3, USER: nmrsu -- DATE: Fri Jul 31 08:11:44 2020

F1: 399.956	F2: 1.000	SW1: 7813		OF1: 2460.0		PTS1d: 65536	
EX: zg30		PW: 12.0 us	PD: 1.0 sec	NA: 16	LB: 0.0	Nuts - $pdata	

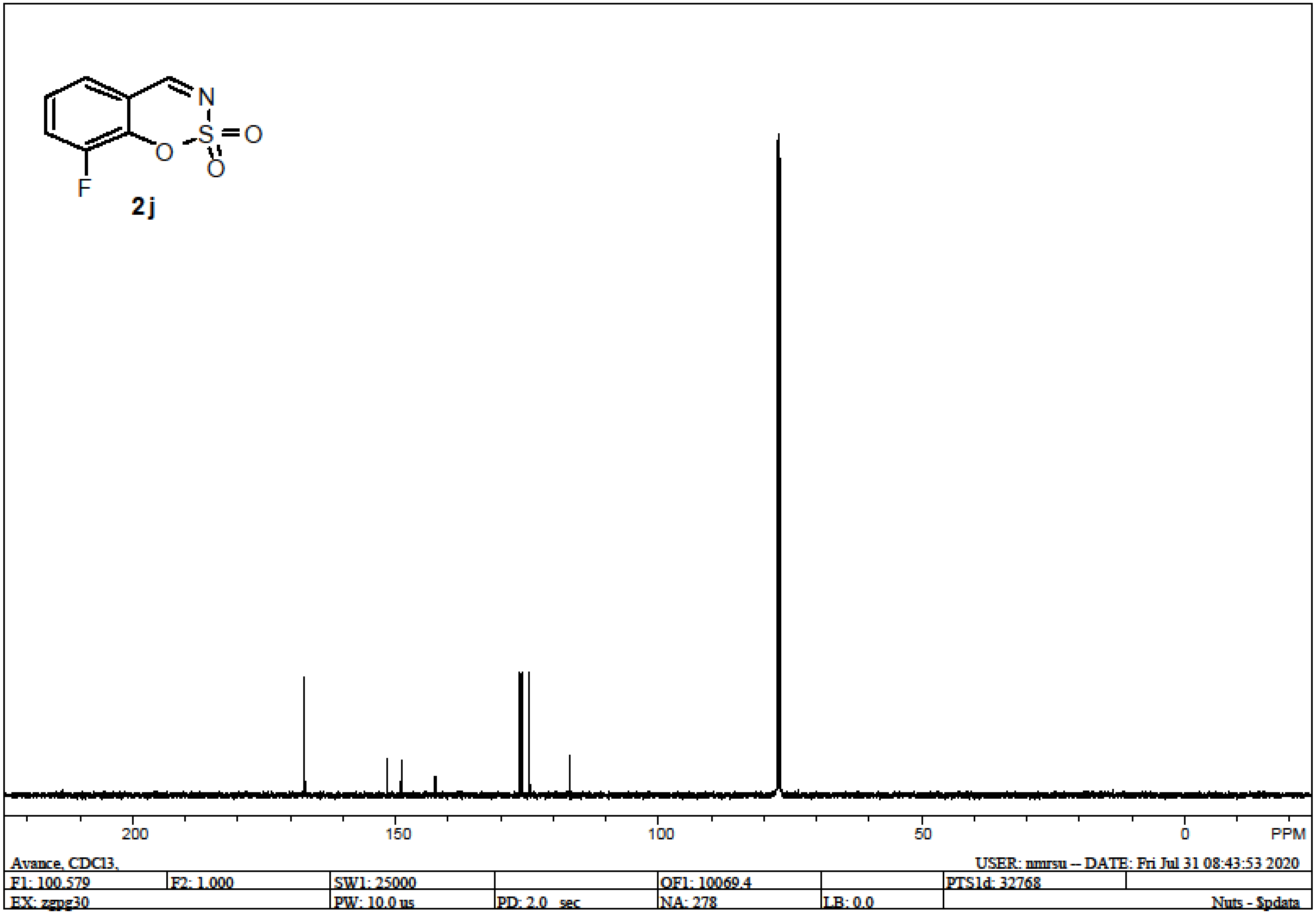

N
S
O
O
O
F
2j
200
150
100
50
0
PPM
Avance, CDCl3,
USER: nmrsu -- DATE: Fri Jul 31 08:43:53 2020
F1: 100.579
F2: 1.000
SW1: 25000
OF1: 10069.4
PTS1d: 32768
EX: zgpg30
PW: 10.0 us
PD: 2.0 sec
NA: 278
LB: 0.0
Nuts - $pdata

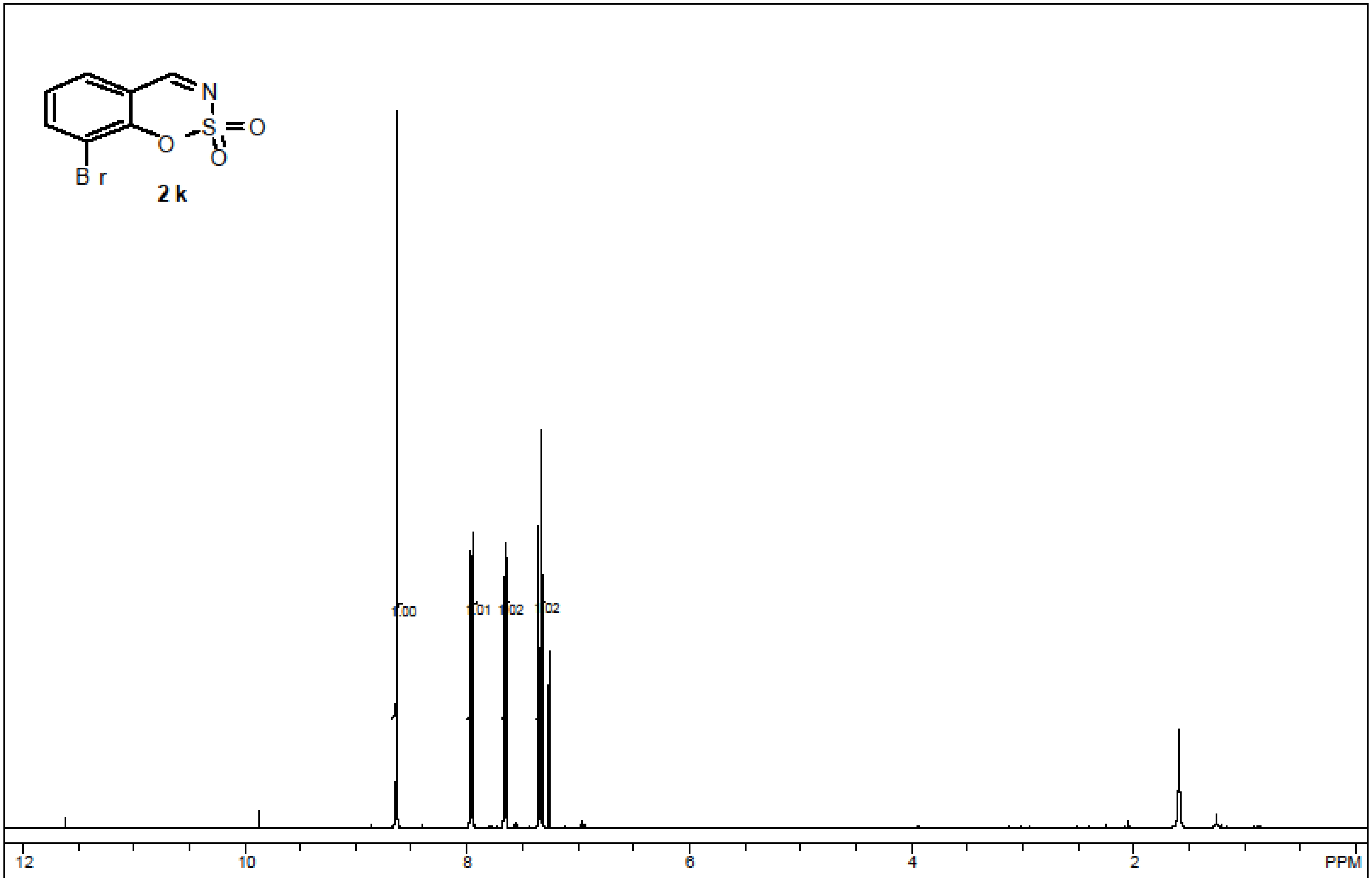

Avance, CDCl3, USER: nmrsu -- DATE: Wed Aug 05 07:40:27 2020

F1: 399.956	F2: 1.000	SW1: 7813		OF1: 2460.1		PTS1d: 65536	
EX: zg30		PW: 12.0 us	PD: 1.0 sec	NA: 16	LB: 0.0	Nuts - $pdata	

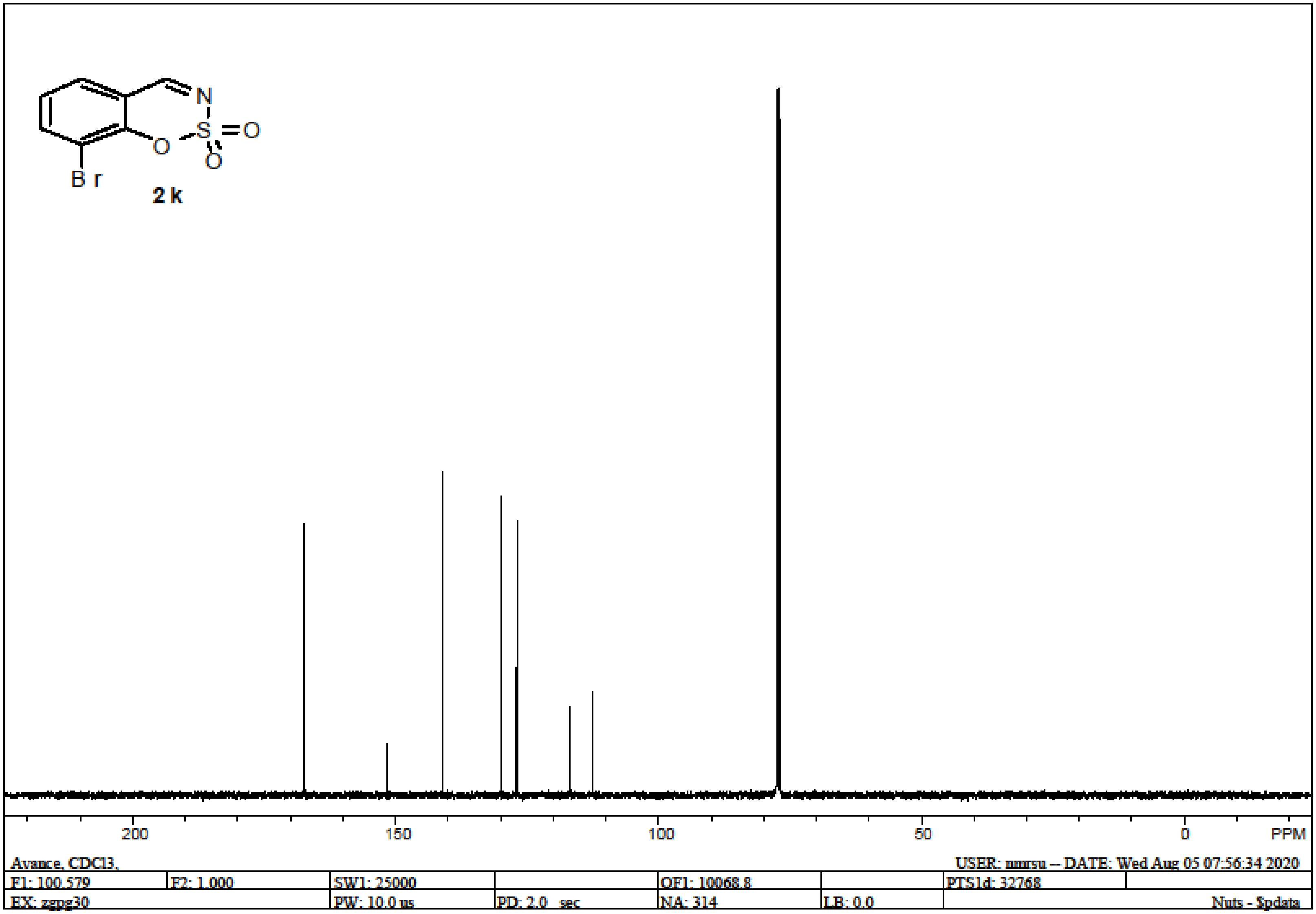

N
S
O
O
O
Br
2 k
200
150
100
50
0
PPM
Avance, CDCl3,
USER: nmrsu -- DATE: Wed Aug 05 07:56:34 2020
F1: 100.579
F2: 1.000
SW1: 25000
OF1: 10068.8
PTS1d: 32768
EX: zgpg30
PW: 10.0 us
PD: 2.0 sec
NA: 314
LB: 0.0
Nuts - $pdata

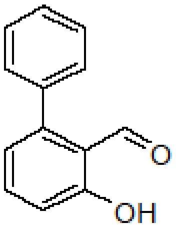
O
OH

4a

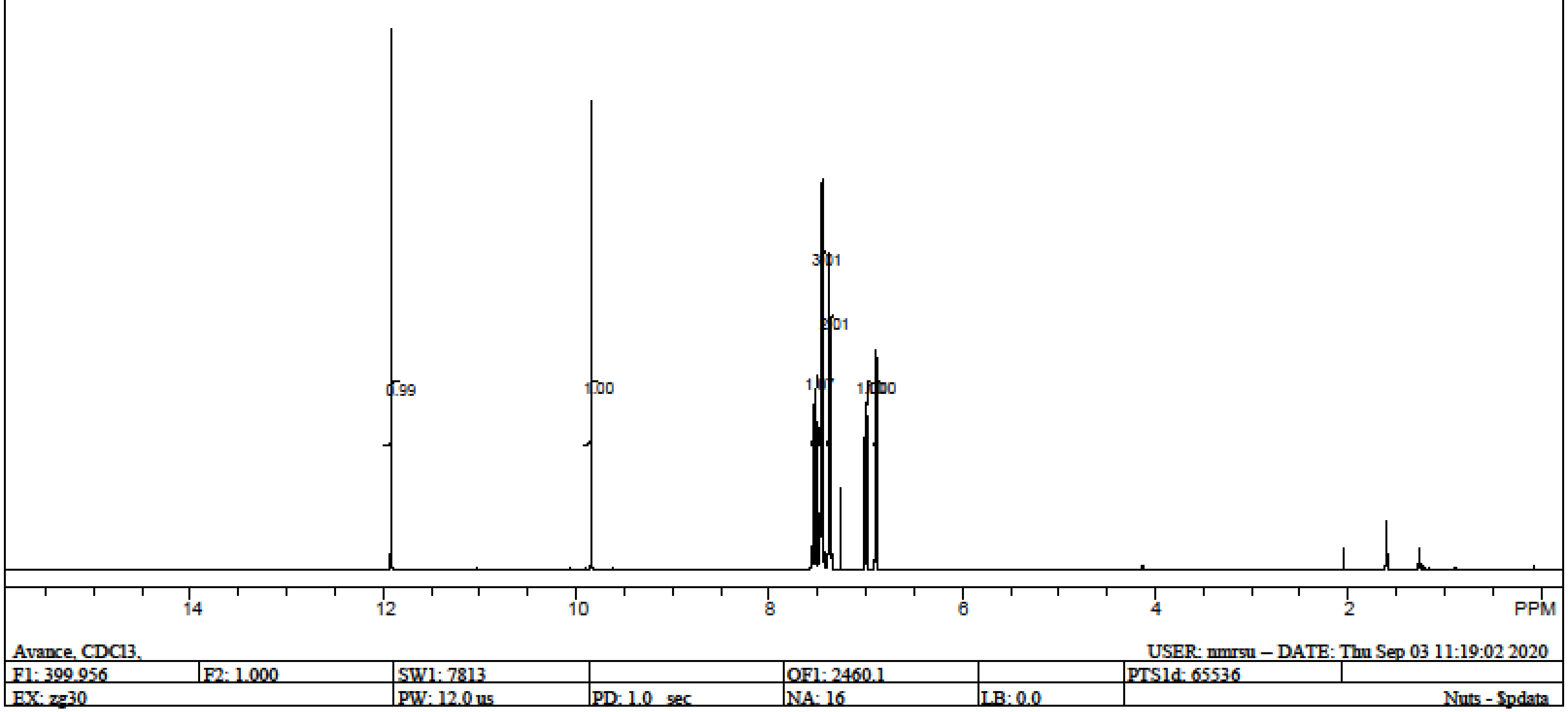
0.99
1.00
3.01
2.01
1.07
1.000
14
12
10
8
6
4
2
PPM
Avance, CDCl3,
USER: nmrsu -- DATE: Thu Sep 03 11:19:02 2020
F1: 399.956
F2: 1.000
SW1: 7813
OF1: 2460.1
PTS1d: 65536
EX: zg30
PW: 12.0 us
PD: 1.0 sec
NA: 16
LB: 0.0
Nuts - $pdata

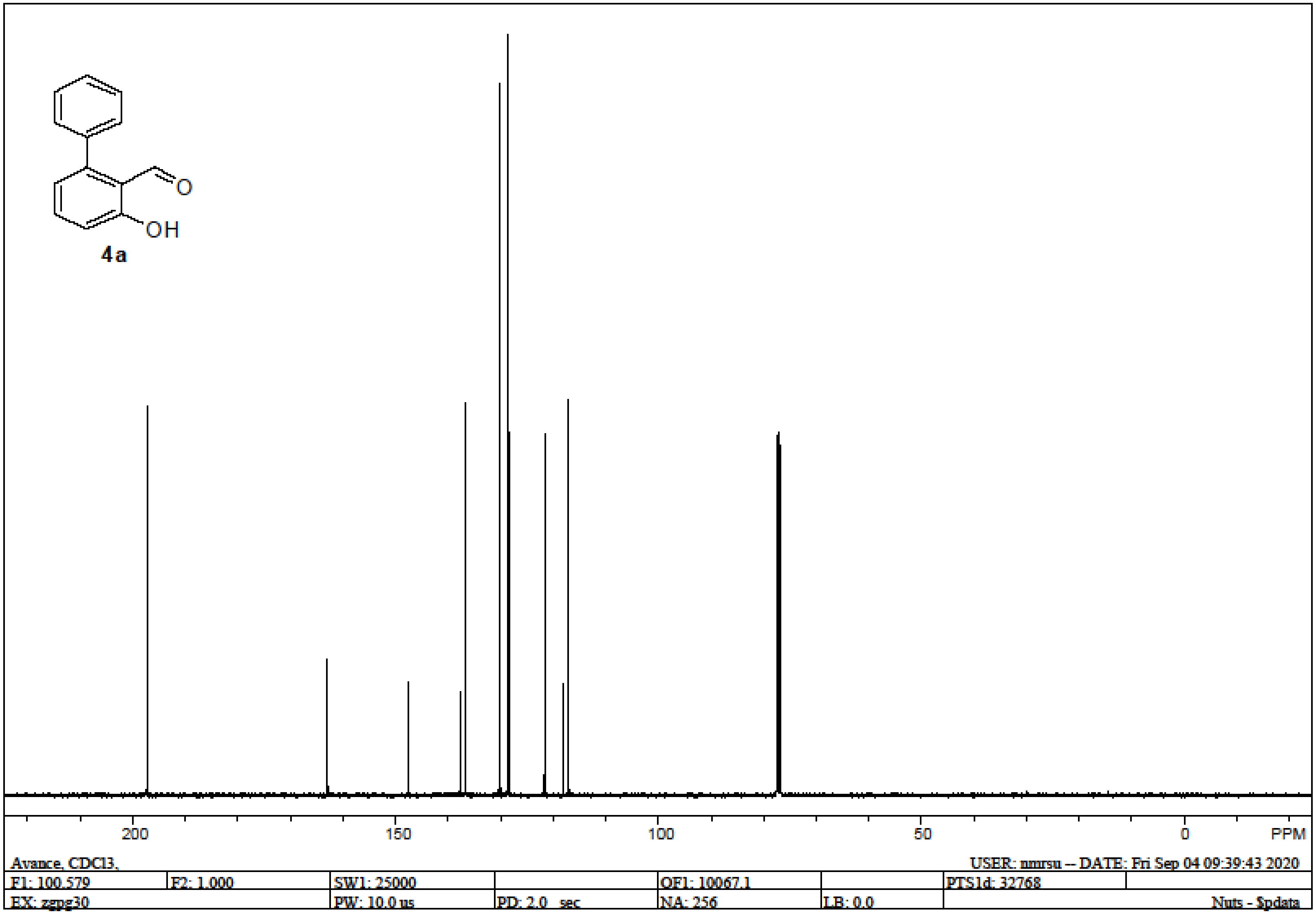
OH
O
4a
200
150
100
50
0
PPM
Avance, CDCl3,
USER: nmrsu -- DATE: Fri Sep 04 09:39:43 2020
F1: 100.579
F2: 1.000
SW1: 25000
OF1: 10067.1
PTS1d: 32768
EX: zgpg30
PW: 10.0 us
PD: 2.0 sec
NA: 256
LB: 0.0
Nuts - $pdata

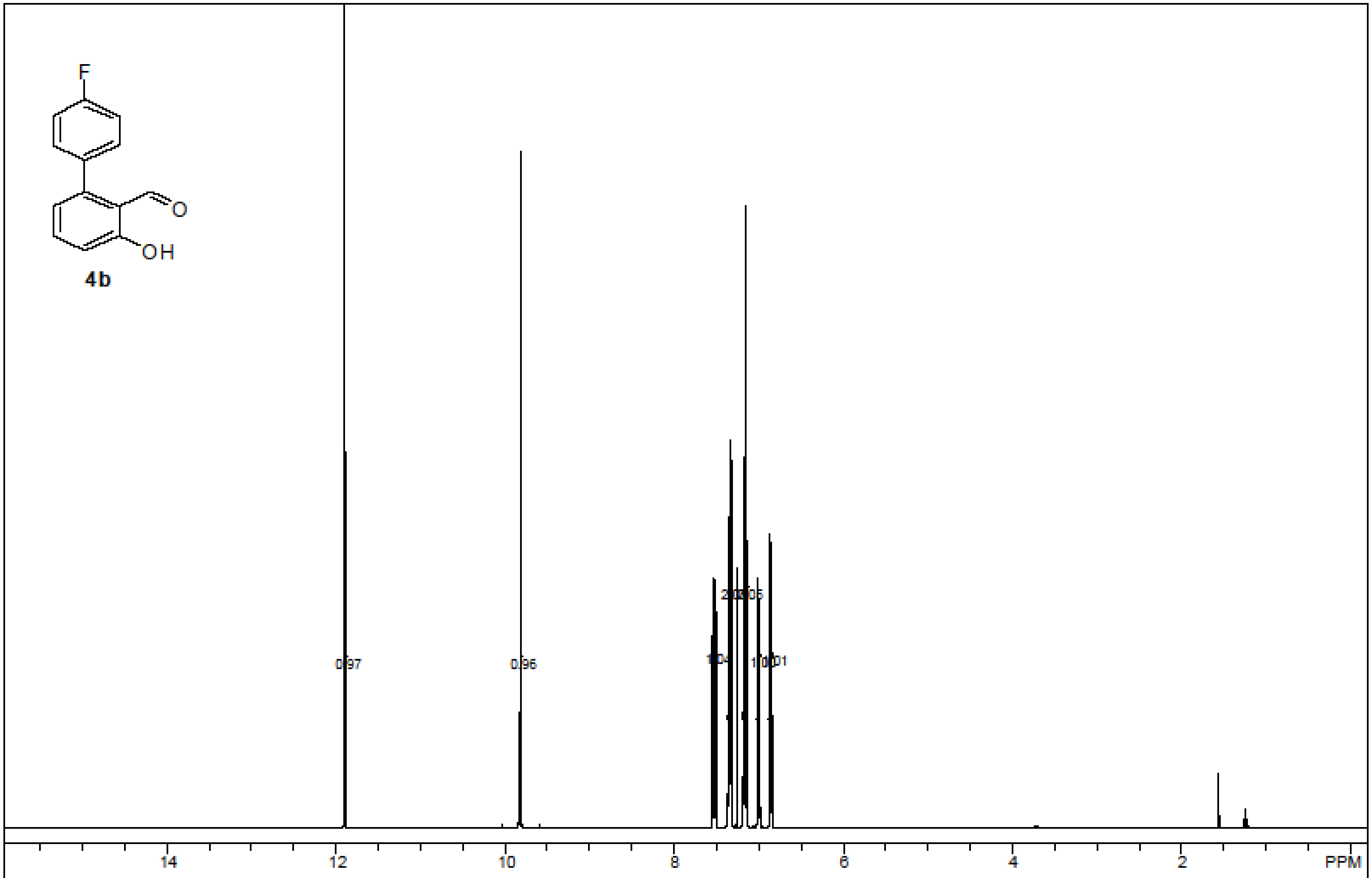

Avance, CDCl3, USER: nmrsu -- DATE: Mon Feb 22 11:19:27 2021

F1: 399.956	F2: 1.000	SW1: 7813		OF1: 2460.1		PTS1d: 65536	
EX: zg30		PW: 12.0 us	PD: 1.0 sec	NA: 16	LB: 0.0		Nuts - $pdata

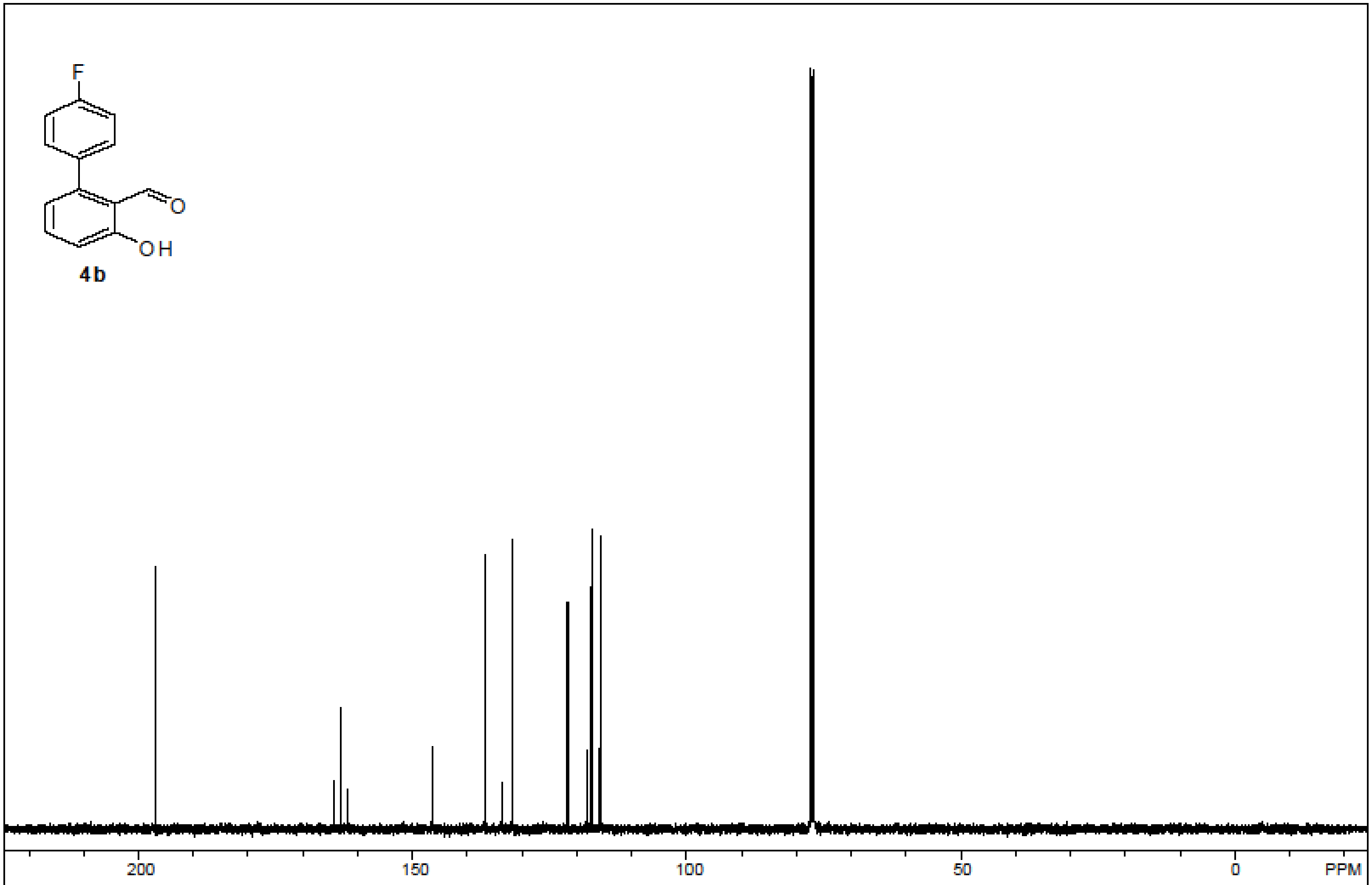

Avance, CDCl3,

USER: nmrsu -- DATE: Mon Feb 22 11:27:10 2021

F1: 100.579	F2: 1.000	SW1: 25000		OF1: 10071.2		PTS1d: 32768	
EX: zgpg30		PW: 10.0 us	PD: 2.0 sec	NA: 150	LB: 0.0	Nuts - $pdata	

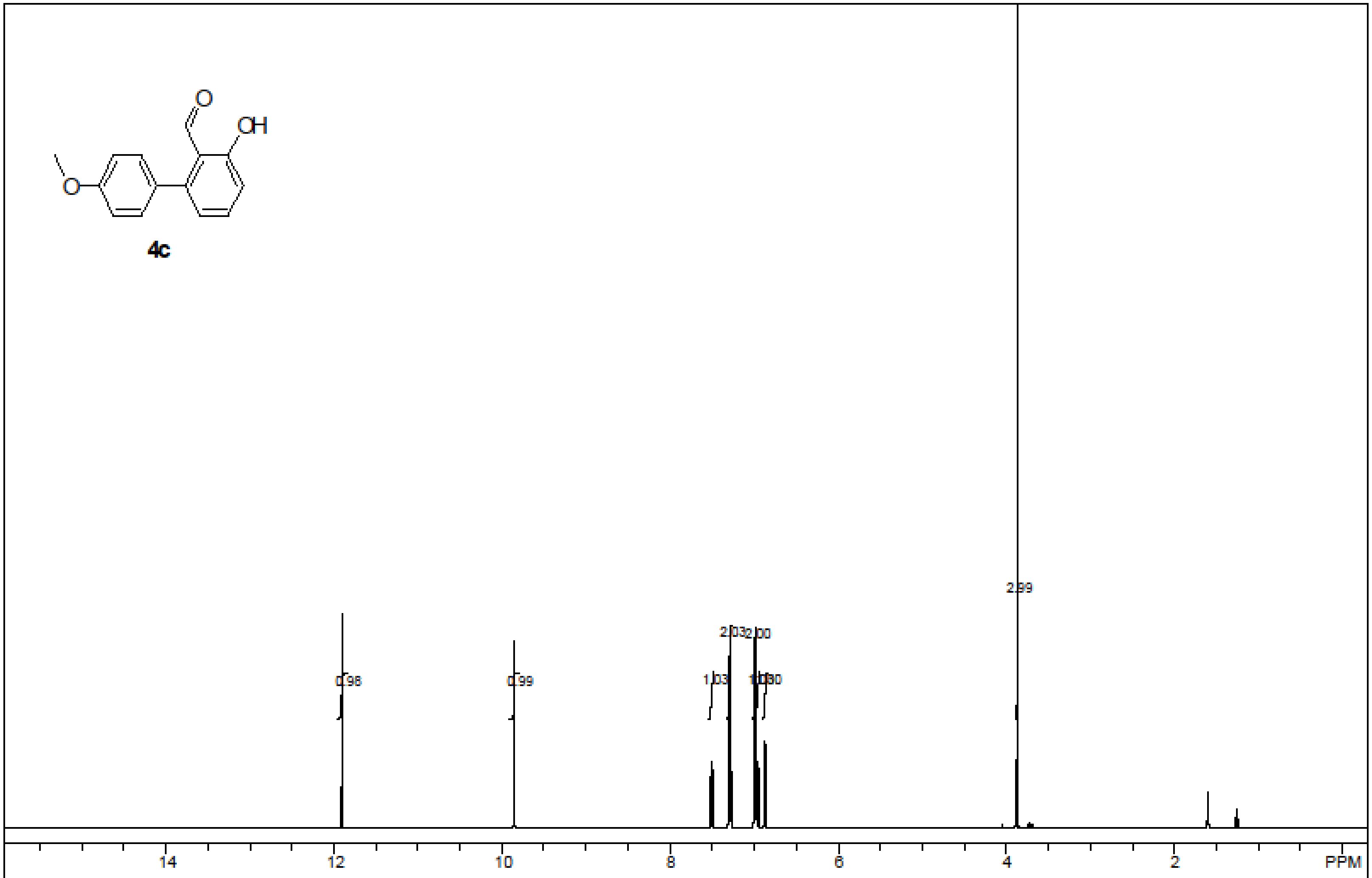

Avance, CDCl3, USER: nmrsu -- DATE: Mon Sep 28 12:20:03 2020

F1: 399.956	F2: 1.000	SW1: 7813		OF1: 2460.3		PTS1d: 65536	
EX: zg30		PW: 12.0 us	PD: 1.0 sec	NA: 16	LB: 0.0		Nuts - $pdata

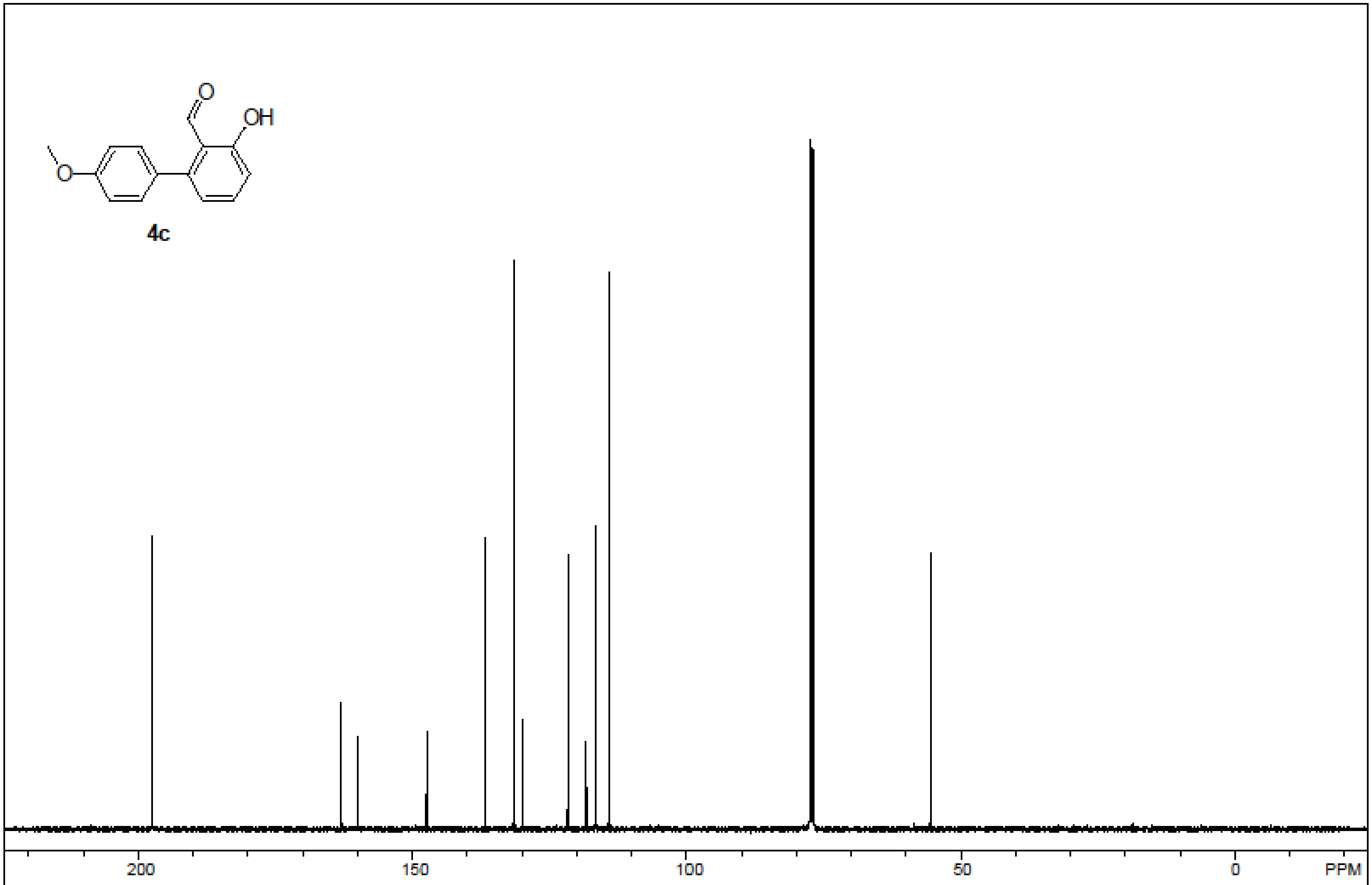

Avance, CDCl3, USER: nmrsu -- DATE: Mon Sep 28 12:44:11 2020

F1: 100.579	F2: 1.000	SW1: 25000		OF1: 10070.5		PTS1d: 32768	
EX: zgpg30		PW: 10.0 us	PD: 2.0 sec	NA: 512	LB: 0.0	Nuts - $pdata	

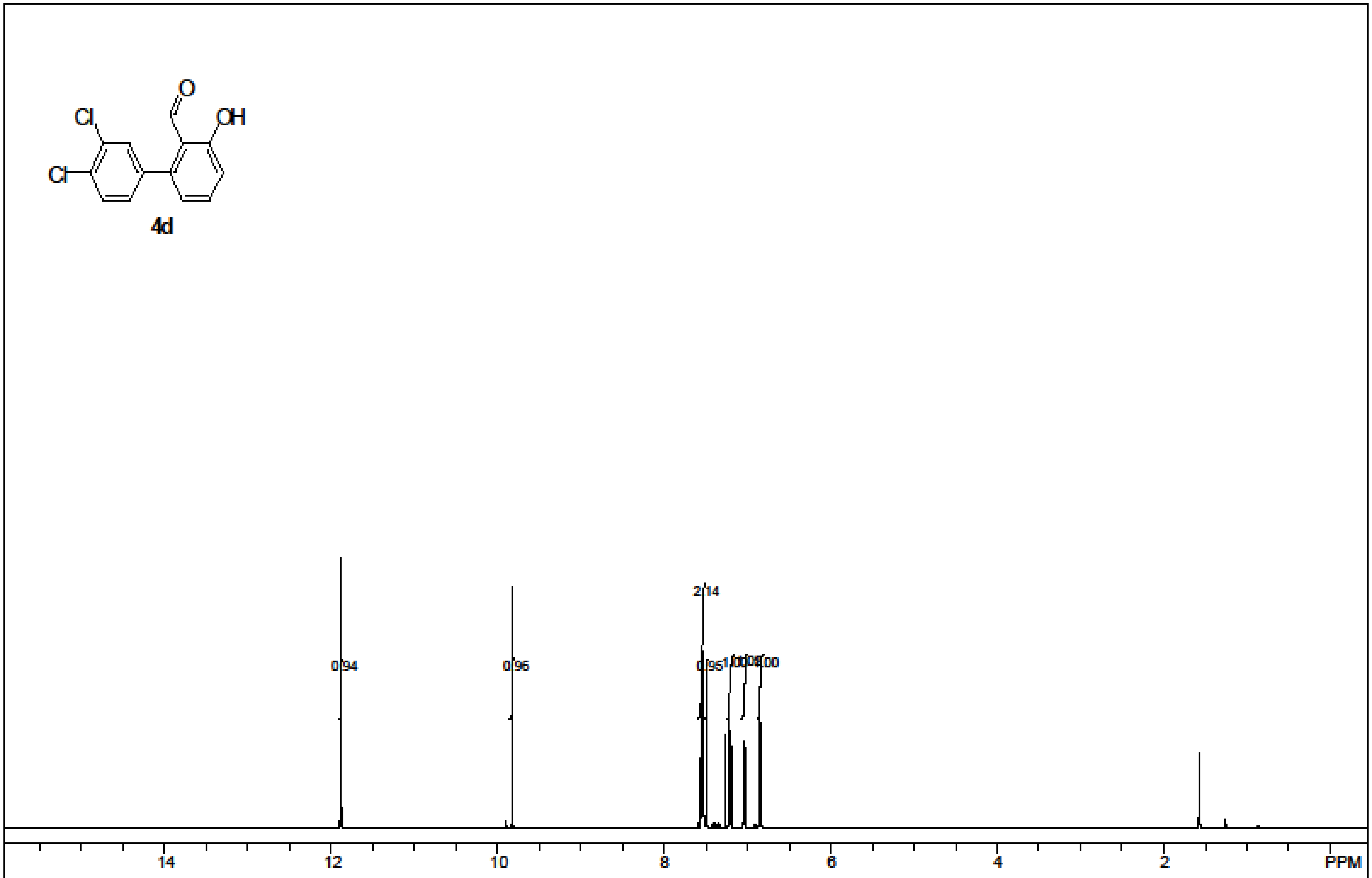

Avance, CDCl3, USER: nmrsu -- DATE: Thu Oct 22 14:37:31 2020

F1: 399.956	F2: 1.000	SW1: 7813		OF1: 2460.1		PTS1d: 65536	
EX: zg30		PW: 12.0 us	PD: 1.0 sec	NA: 16	LB: 0.0		Nuts - $pdata

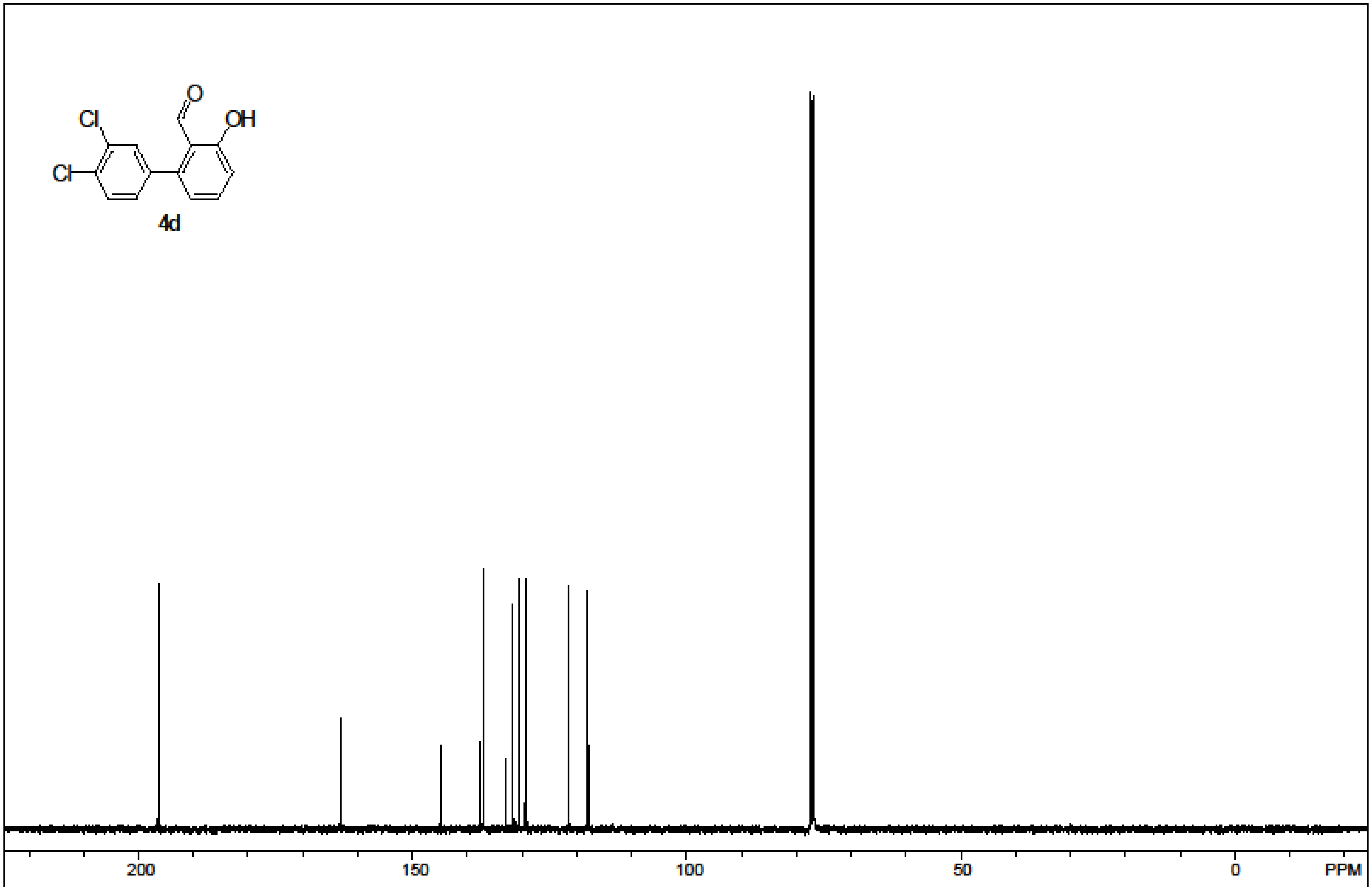

Avance, CDCl3, USER: nmrsu -- DATE: Thu Oct 22 14:52:56 2020

F1: 100.579	F2: 1.000	SW1: 25000		OF1: 10071.2		PTS1d: 32768	
EX: zgpg30		PW: 10.0 us	PD: 2.0 sec	NA: 320	LB: 0.0		Nuts - $pdata

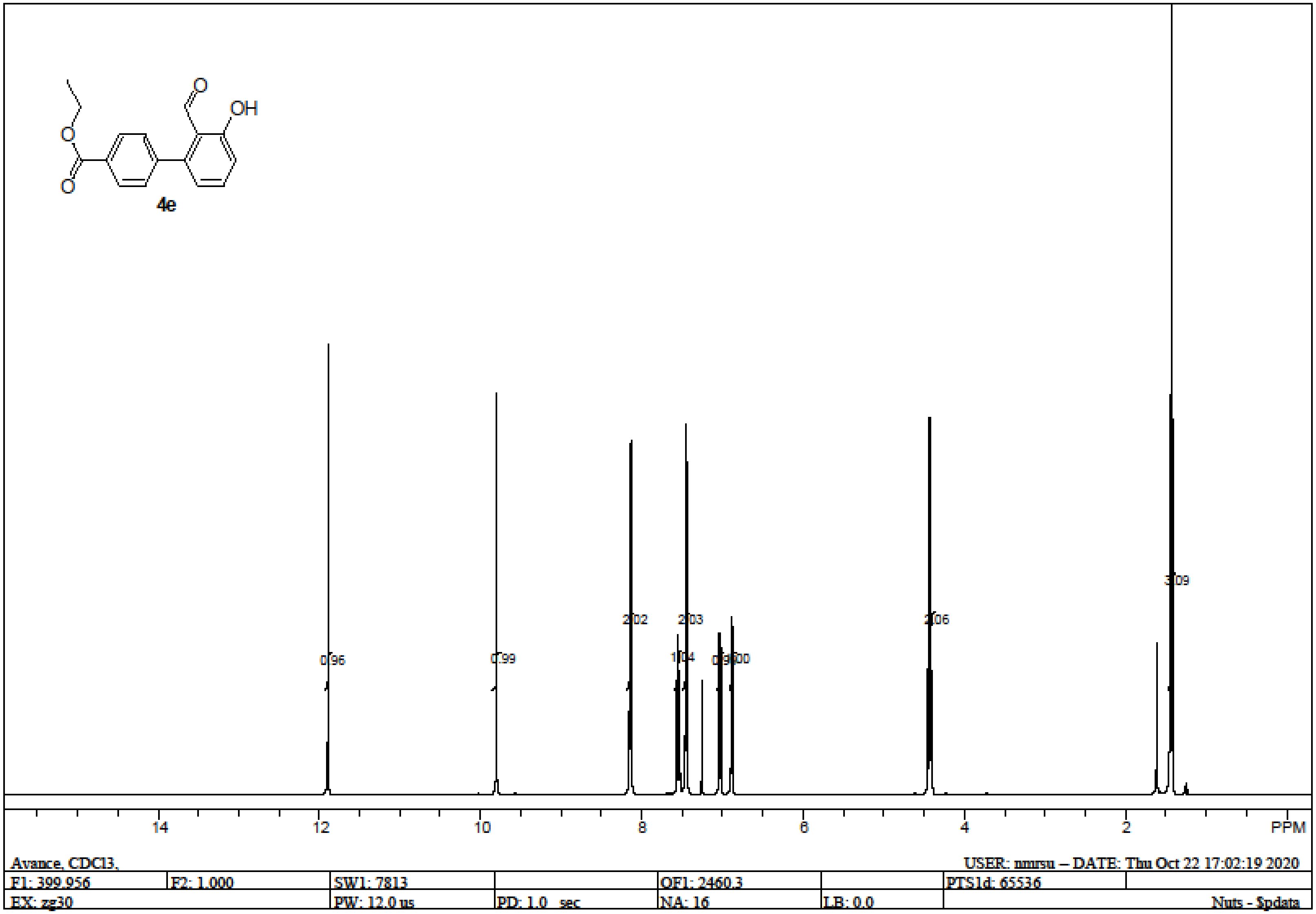
OH
O
O
O
4e
0.96
0.99
2.02
2.03
1.04
0.96
1.00
2.06
3.09
14
12
10
8
6
4
2
PPM
Avance, CDCl3,
USER: nmrsu -- DATE: Thu Oct 22 17:02:19 2020
F1: 399.956
F2: 1.000
SW1: 7813
OF1: 2460.3
PTS1d: 65536
EX: zg30
PW: 12.0 us
PD: 1.0 sec
NA: 16
LB: 0.0
Nuts - $pdata

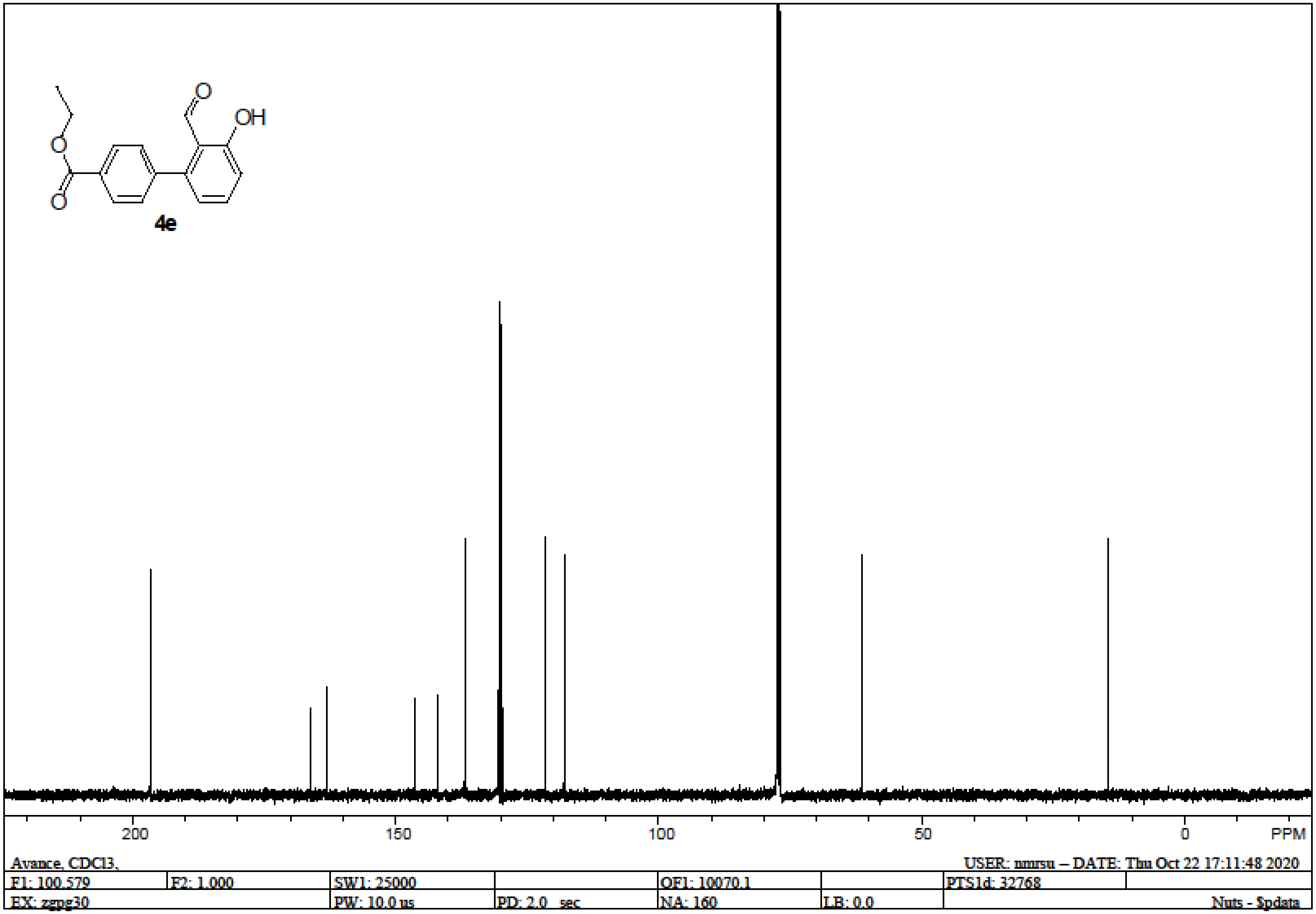

OH
O
O
O
4e
200
150
100
50
0
PPM
Avance, CDCl3,
USER: nmrsu -- DATE: Thu Oct 22 17:11:48 2020
F1: 100.579
F2: 1.000
SW1: 25000
OF1: 10070.1
PTS1d: 32768
EX: zgpg30
PW: 10.0 us
PD: 2.0 sec
NA: 160
LB: 0.0
Nuts - $pdata

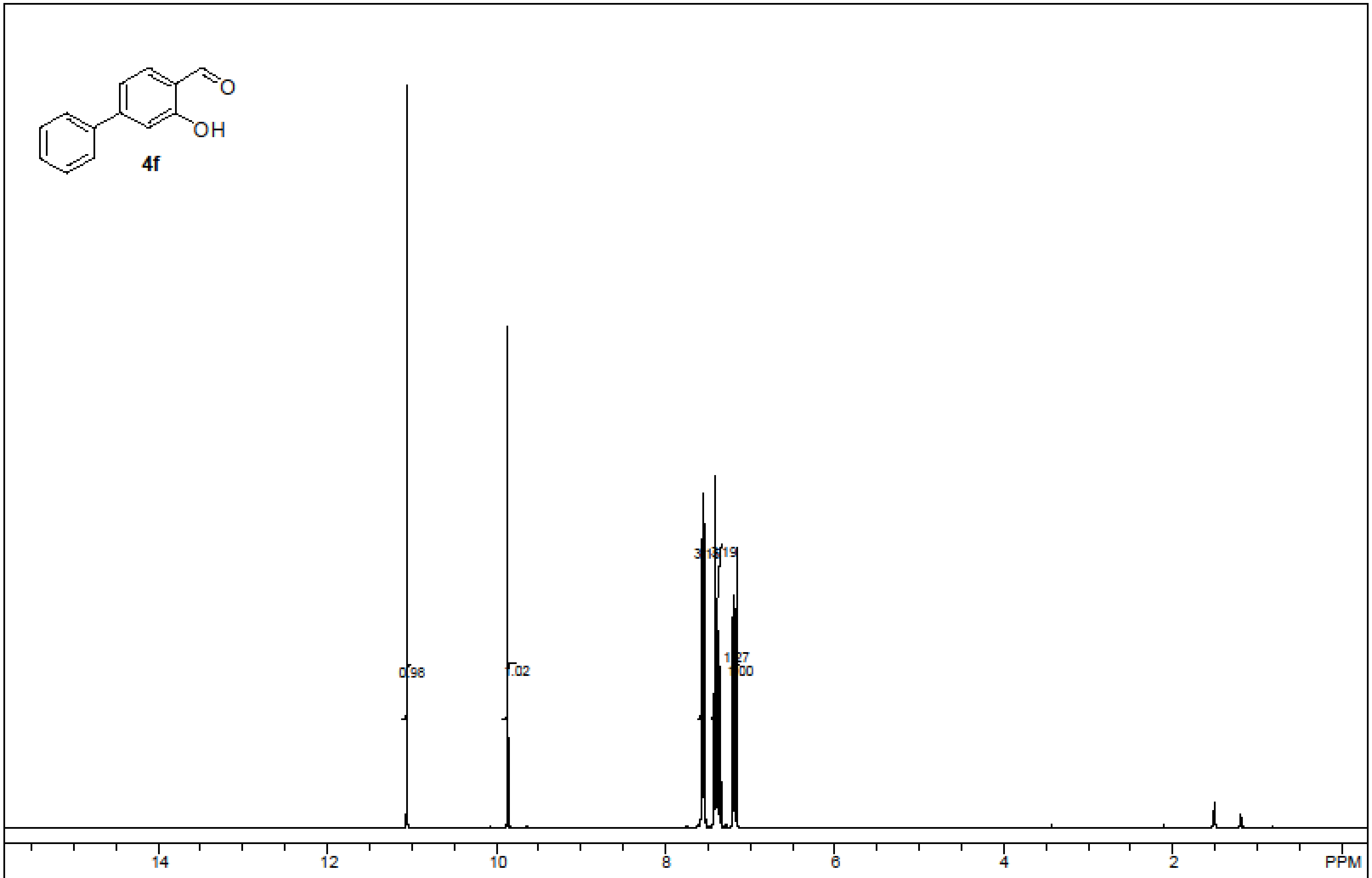

Avance, CDCl3, USER: nmrsu -- DATE: Fri Dec 11 12:08:06 2020

F1: 399.956	F2: 1.000	SW1: 7813		OF1: 2434.4		PTS1d: 65536	
EX: zg30		PW: 12.0 us	PD: 1.0 sec	NA: 16	LB: 0.0	Nuts - $pdata	

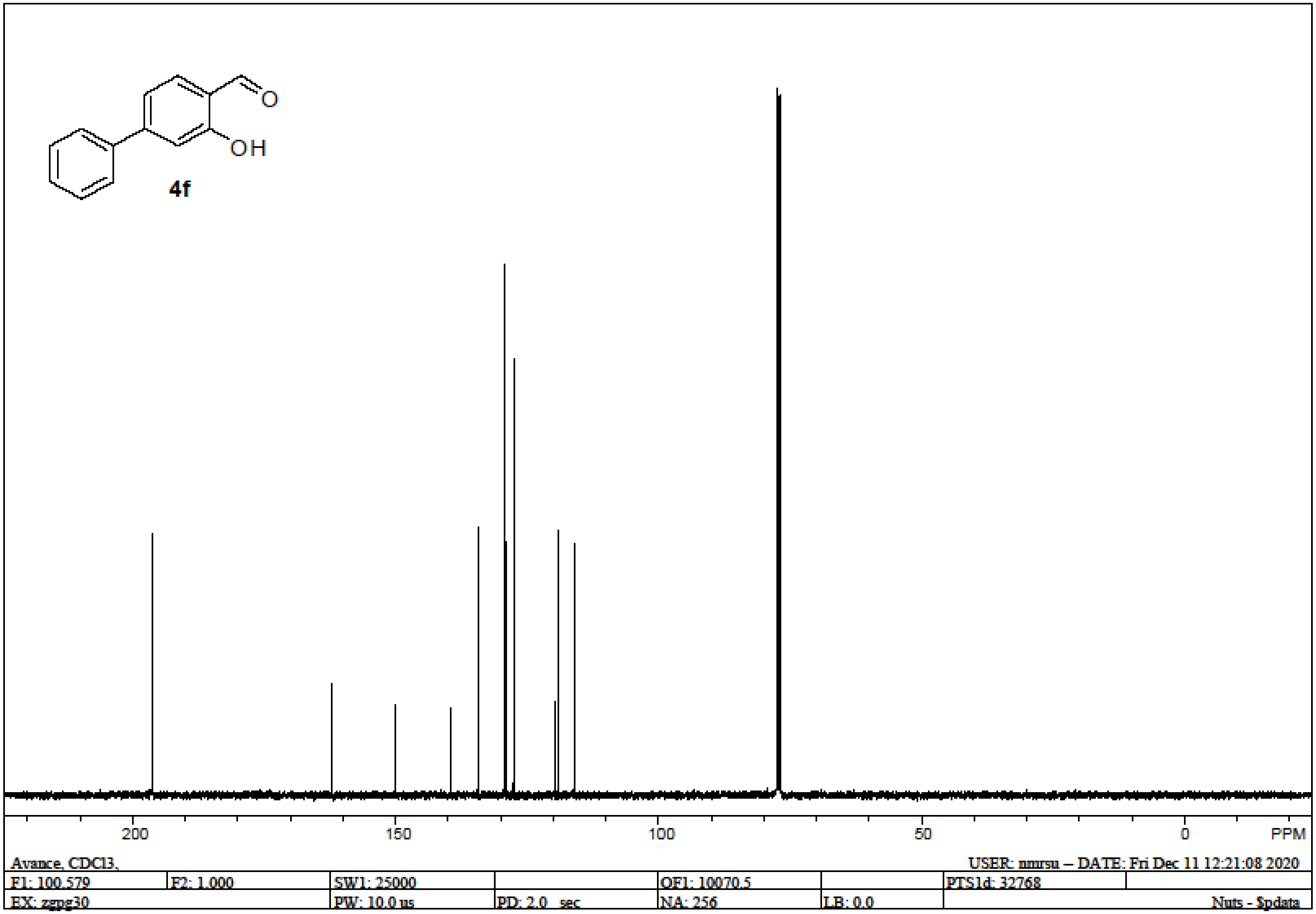
OH
4f
200
150
100
50
0
PPM
Avance, CDCl3,
USER: nmrsu -- DATE: Fri Dec 11 12:21:08 2020
F1: 100.579
F2: 1.000
SW1: 25000
OF1: 10070.5
PTS1d: 32768
EX: zgpg30
PW: 10.0 us
PD: 2.0 sec
NA: 256
LB: 0.0
Nuts - $pdata

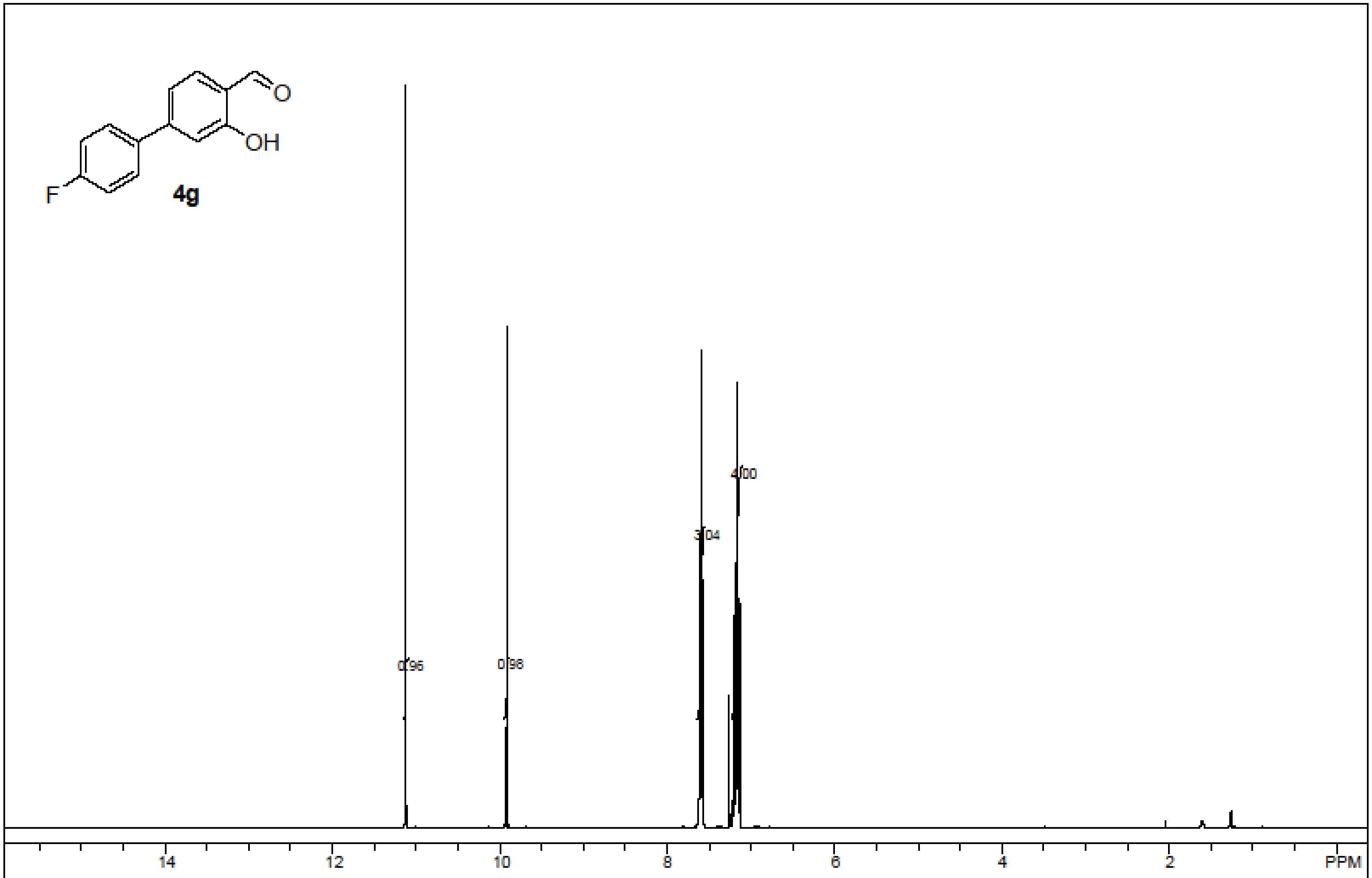

Avance, CDCl3, USER: nmrsu -- DATE: Fri Dec 11 11:50:19 2020

F1: 399.956	F2: 1.000	SW1: 7813		OF1: 2460.1		PTS1d: 65536	
EX: zg30		PW: 12.0 us	PD: 1.0 sec	NA: 16	LB: 0.0		Nuts - $pdata

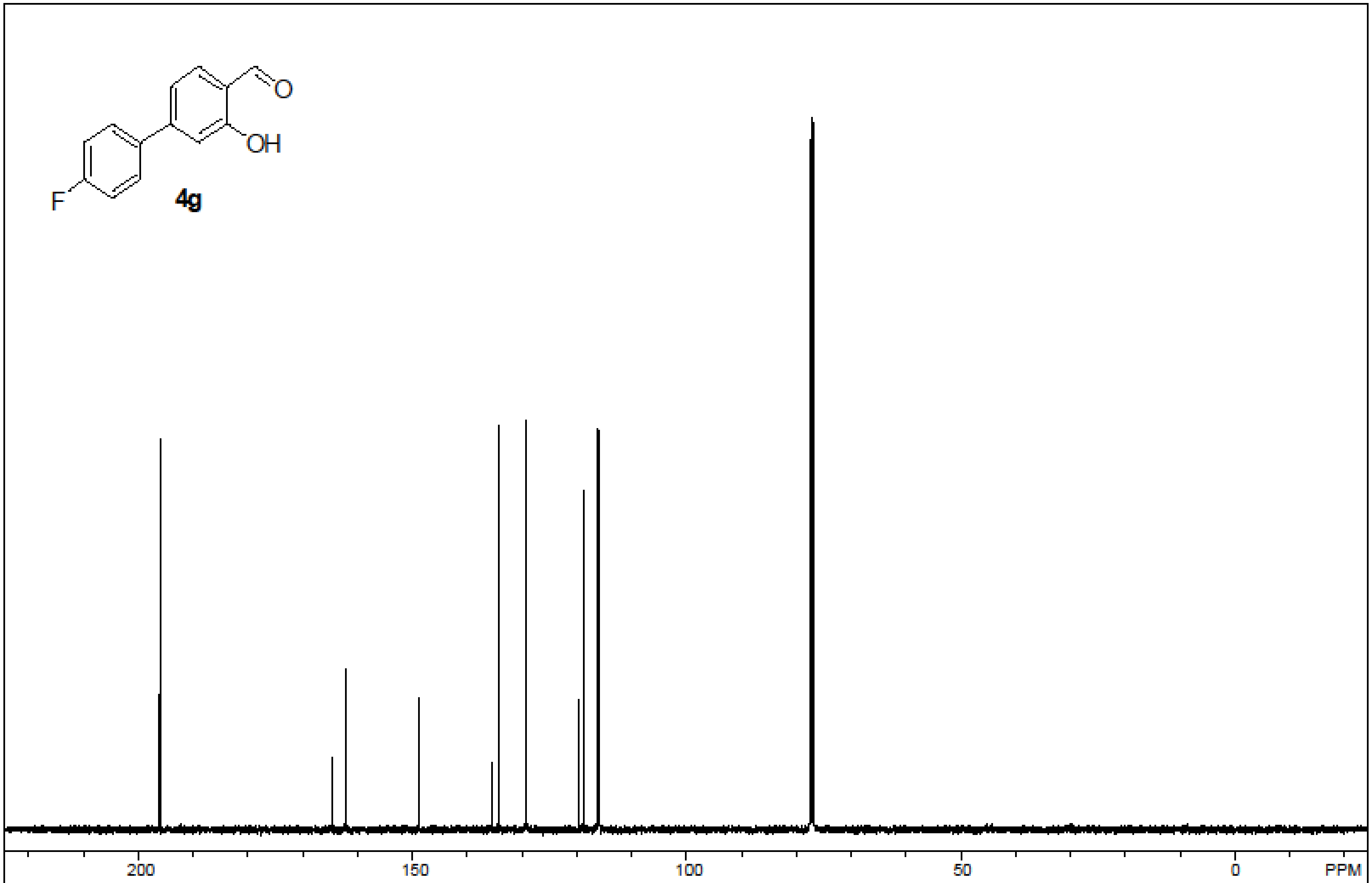

Avance, CDCl3, USER: nmrsu -- DATE: Fri Dec 11 12:03:38 2020

F1: 100.579	F2: 1.000	SW1: 25000		OF1: 10069.2		PTS1d: 32768	
EX: zgpg30		PW: 10.0 us	PD: 2.0 sec	NA: 256	LB: 0.0		Nuts - $pdata

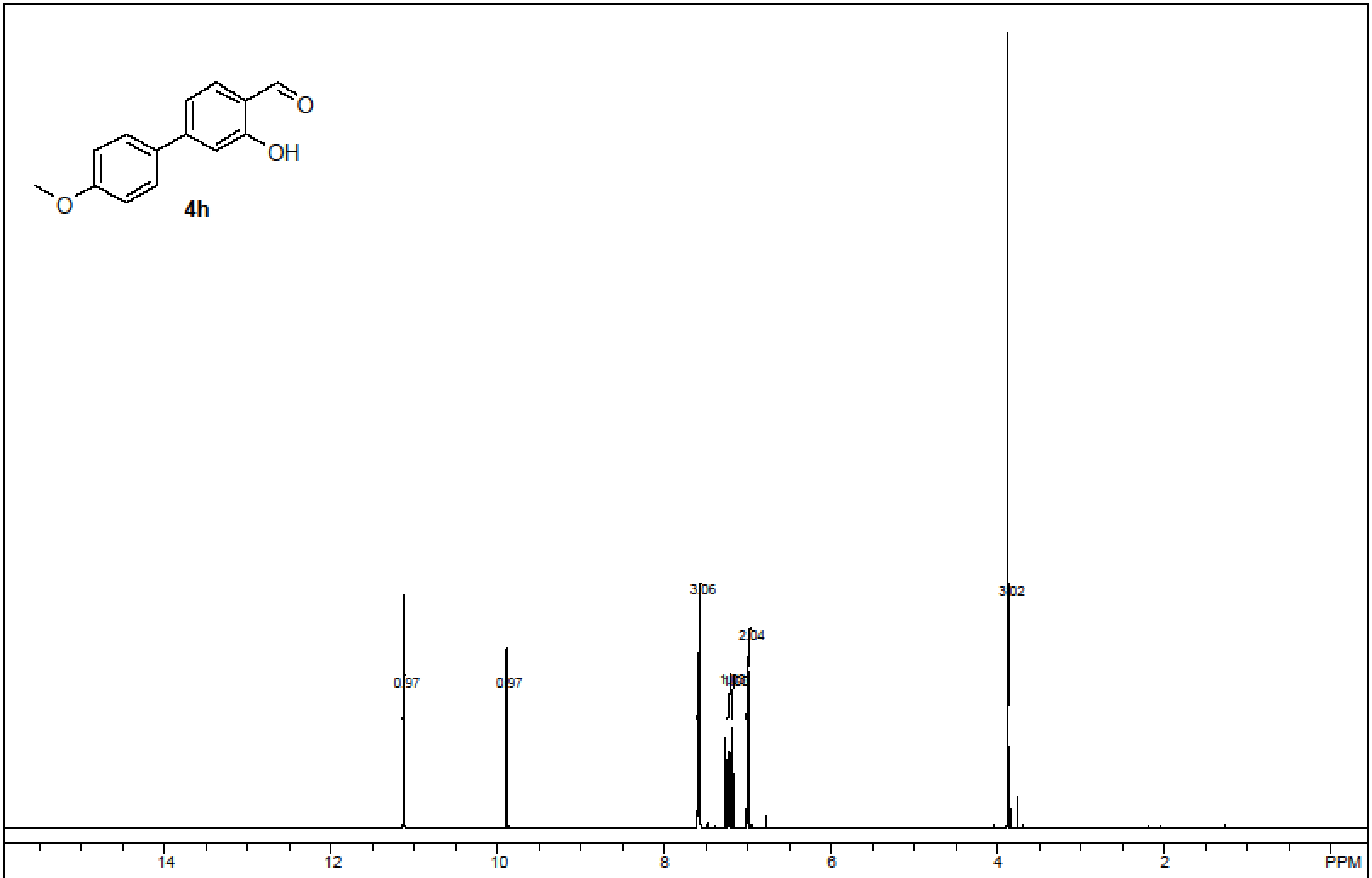

Avance, CDCl3,

USER: nmrsu -- DATE: Mon Dec 14 16:07:57 2020

F1: 399.956	F2: 1.000	SW1: 7813		OF1: 2460.1		PTS1d: 65536	
EX: zg30		PW: 12.0 us	PD: 1.0 sec	NA: 16	LB: 0.0	Nuts - $pdata	

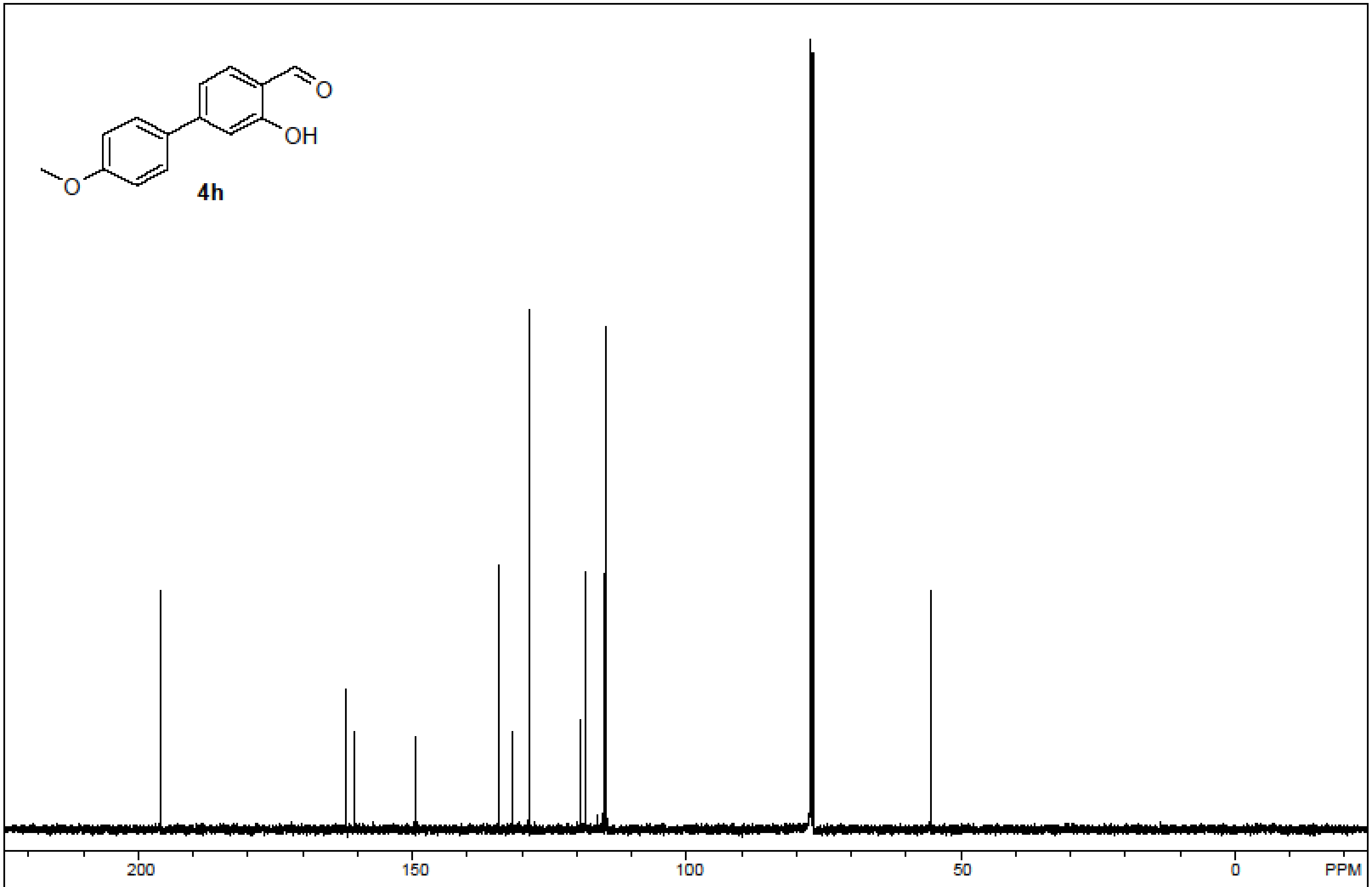

Avance, CDCl3, USER: nmrsu -- DATE: Mon Dec 14 16:17:47 2020

F1: 100.579	F2: 1.000	SW1: 25000		OF1: 10070.5		PTS1d: 32768	
EX: zgpg30		PW: 10.0 us	PD: 2.0 sec	NA: 180	LB: 0.0	Nuts - $pdata	

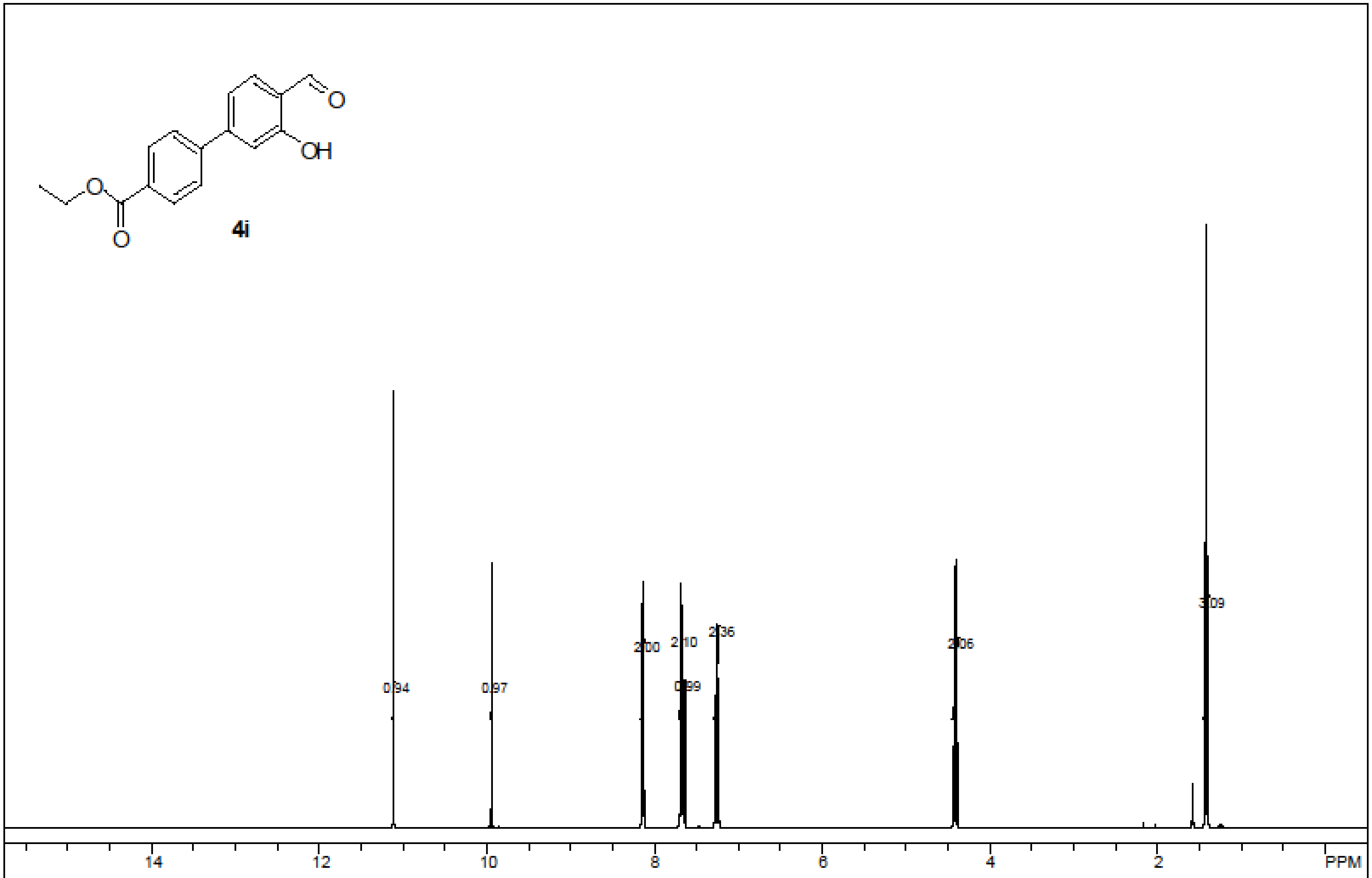

Avance, CDCl3,							USER: nmrsu -- DATE: Mon Dec 14 16:37:46 2020
F1: 399.956	F2: 1.000	SW1: 7813		OF1: 2460.1		PTS1d: 65536	
EX: zg30		PW: 12.0 us	PD: 1.0 sec	NA: 16	LB: 0.0		Nuts - $pdata

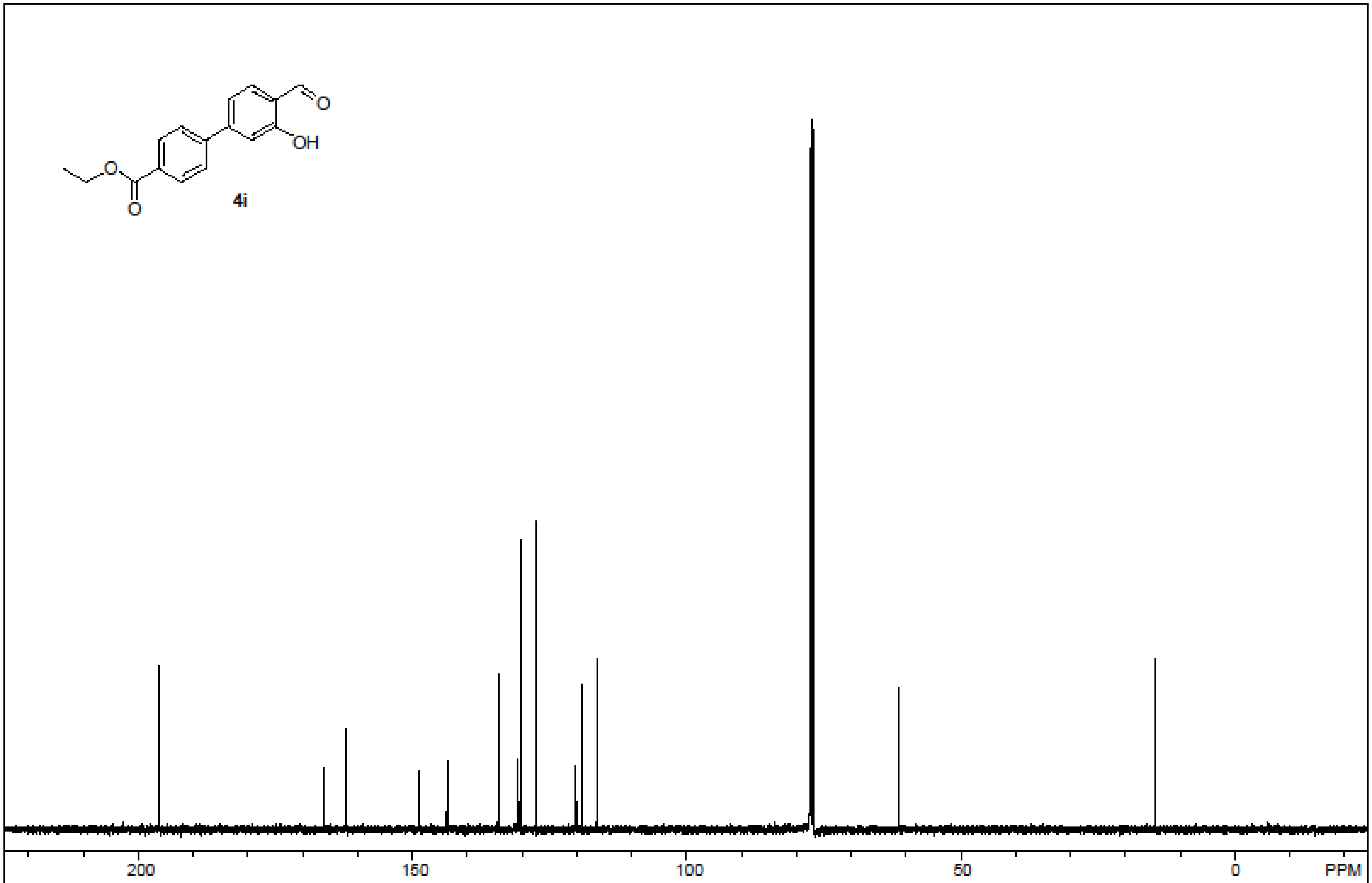

Avance, CDCl3,

USER: nmrsu -- DATE: Mon Dec 14 16:47:39 2020

F1: 100.579	F2: 1.000	SW1: 25000		OF1: 10070.2		PTS1d: 32768	
EX: zgpg30		PW: 10.0 us	PD: 2.0 sec	NA: 180	LB: 0.0	Nuts - $pdata	

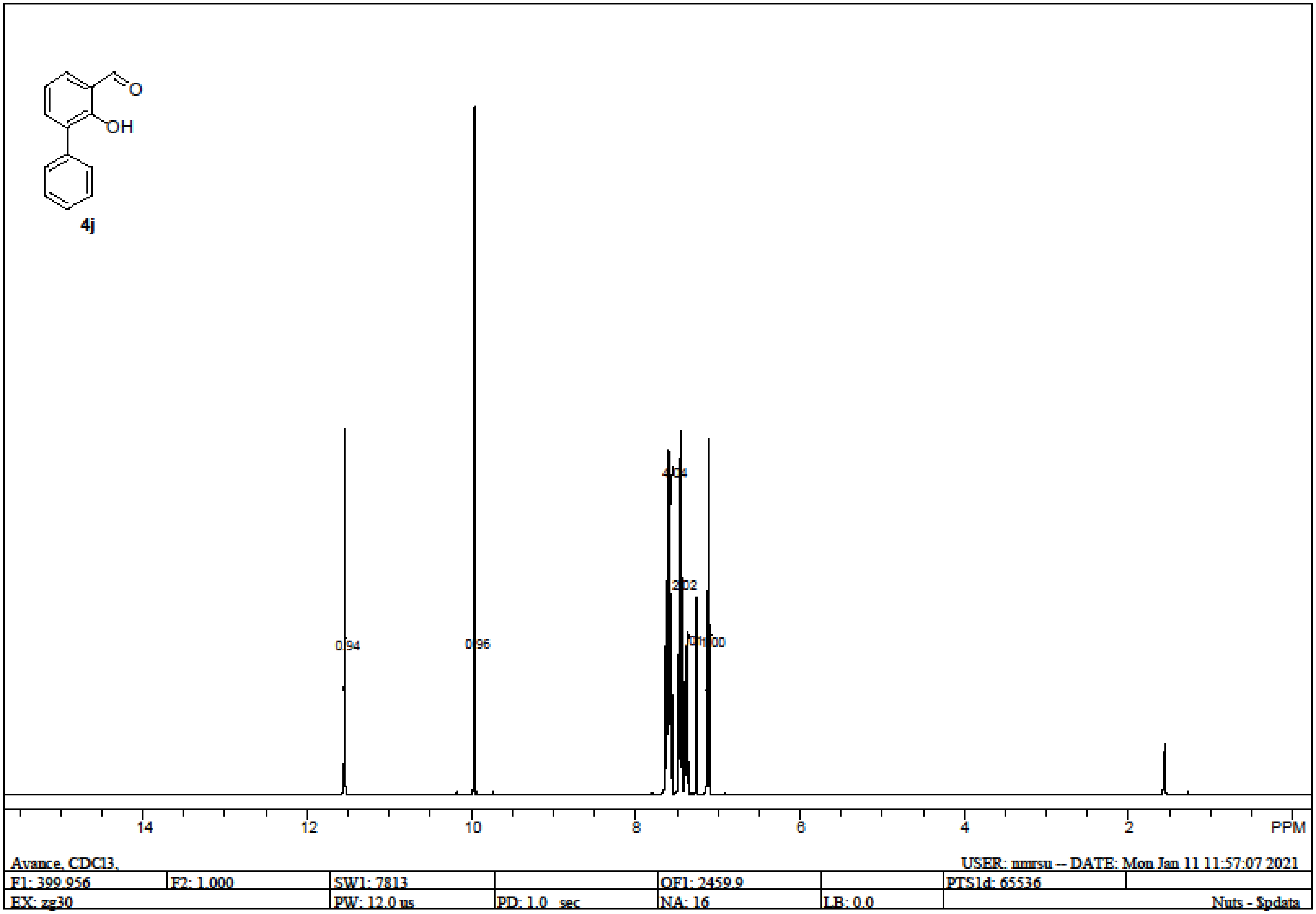

OH
O
4j
0.94
0.96
4.04
2.02
0.1
1.00
14
12
10
8
6
4
2
PPM
Avance, CDCl3,
USER: nmrsu -- DATE: Mon Jan 11 11:57:07 2021
F1: 399.956
F2: 1.000
SW1: 7813
OF1: 2459.9
PTS1d: 65536
EX: zg30
PW: 12.0 us
PD: 1.0 sec
NA: 16
LB: 0.0
Nuts - $pdata

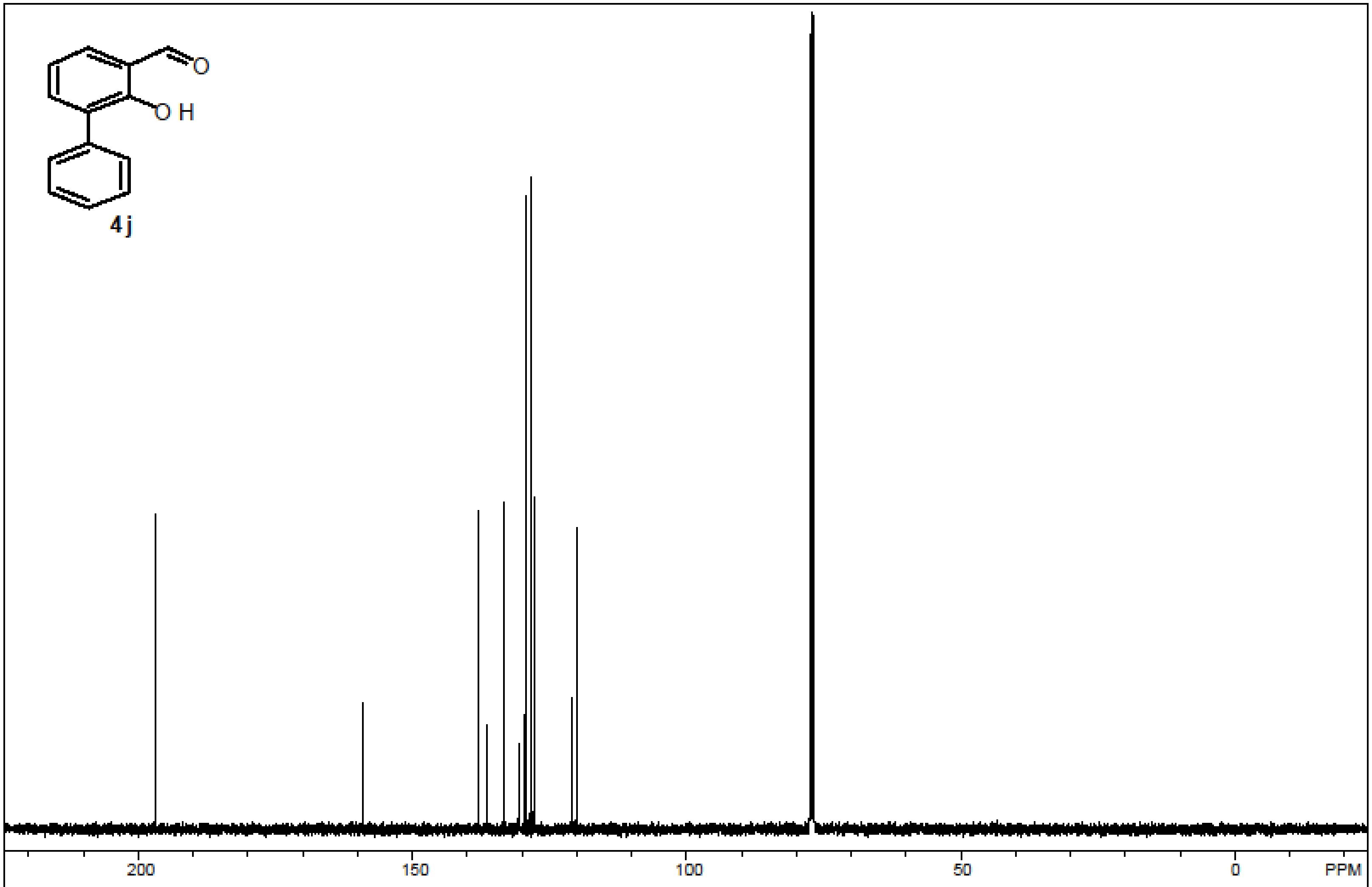

Avance, CDCl3, USER: nmrsu -- DATE: Mon Jan 11 12:06:45 2021

F1: 100.579	F2: 1.000	SW1: 25000		OF1: 10069.3		PTS1d: 32768	
EX: zgpg30		PW: 10.0 us	PD: 2.0 sec	NA: 183	LB: 0.0		Nuts - $pdata

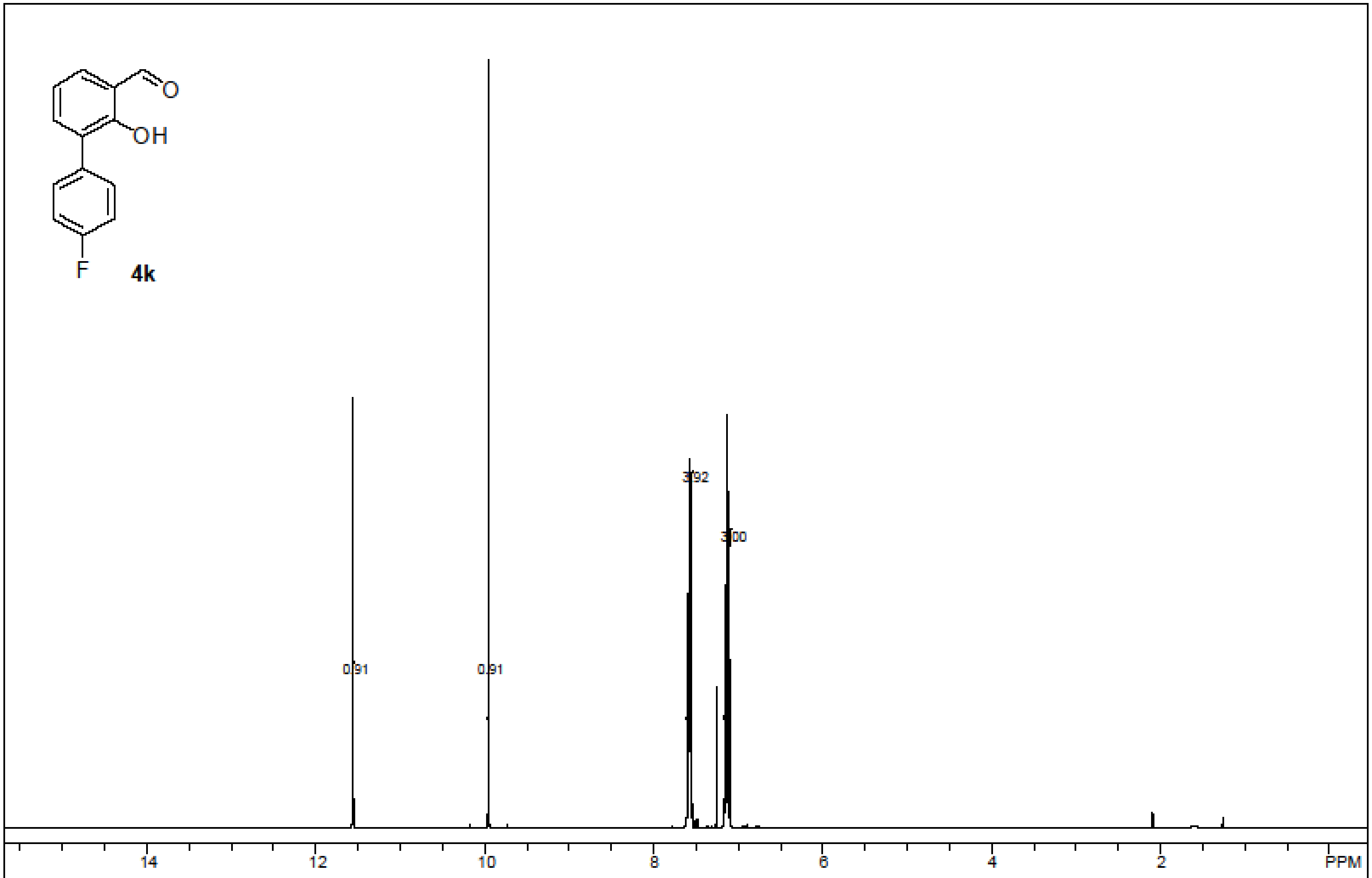

Avance, CDCl3,						USER: nmrsu -- DATE: Mon Jan 11 11:39:12 2021	
F1: 399.956	F2: 1.000	SW1: 7813		OF1: 2460.0		PTS1d: 65536	
EX: zg30		PW: 12.0 us	PD: 1.0 sec	NA: 16	LB: 0.0		Nuts - $pdata

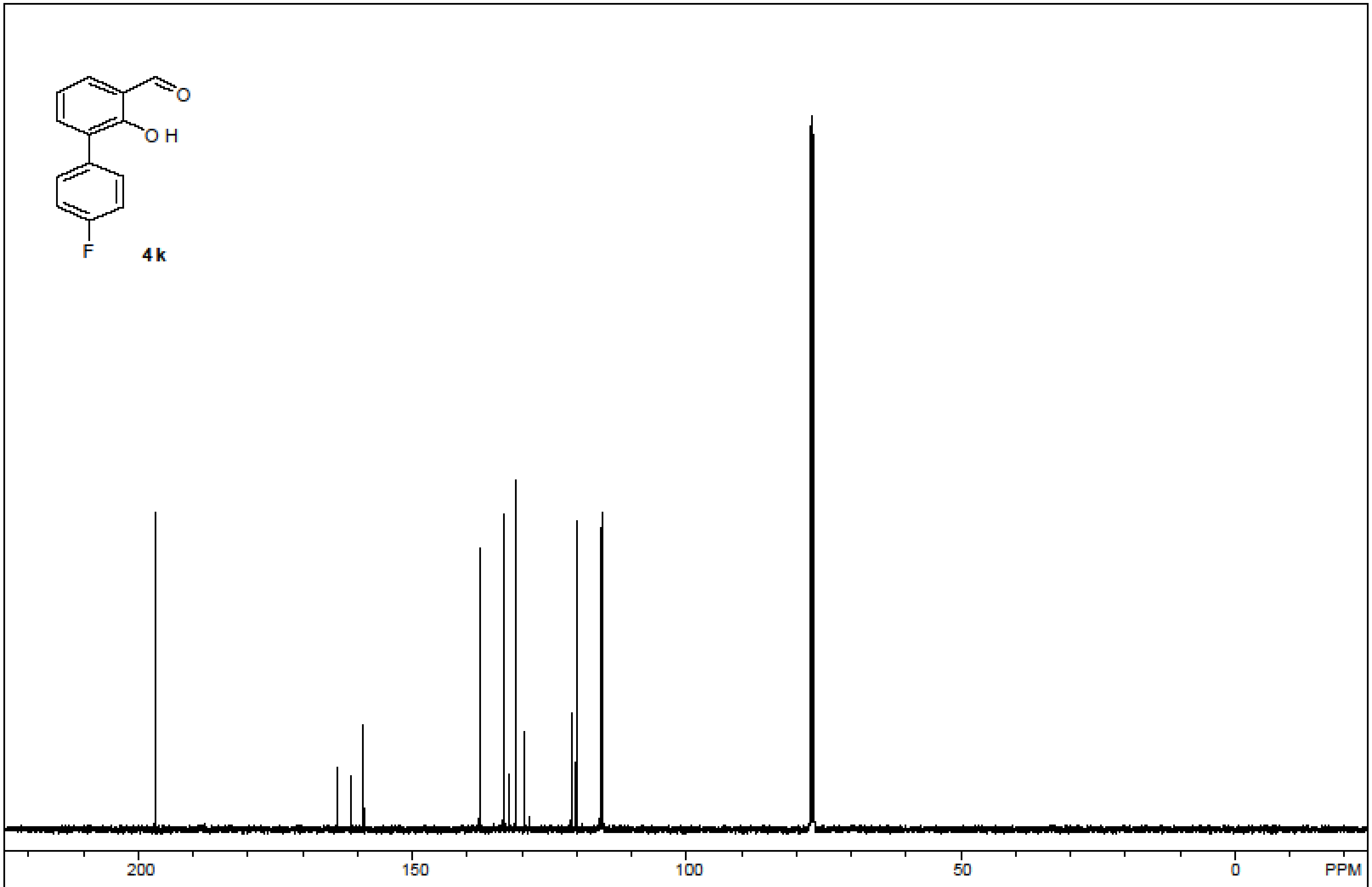

Avance, CDCl3, USER: nmrsu -- DATE: Mon Jan 11 11:52:40 2021

F1: 100.579	F2: 1.000	SW1: 25000		OF1: 10069.2		PTS1d: 32768	
EX: zgpg30		PW: 10.0 us	PD: 2.0 sec	NA: 256	LB: 0.0	Nuts - $pdata	

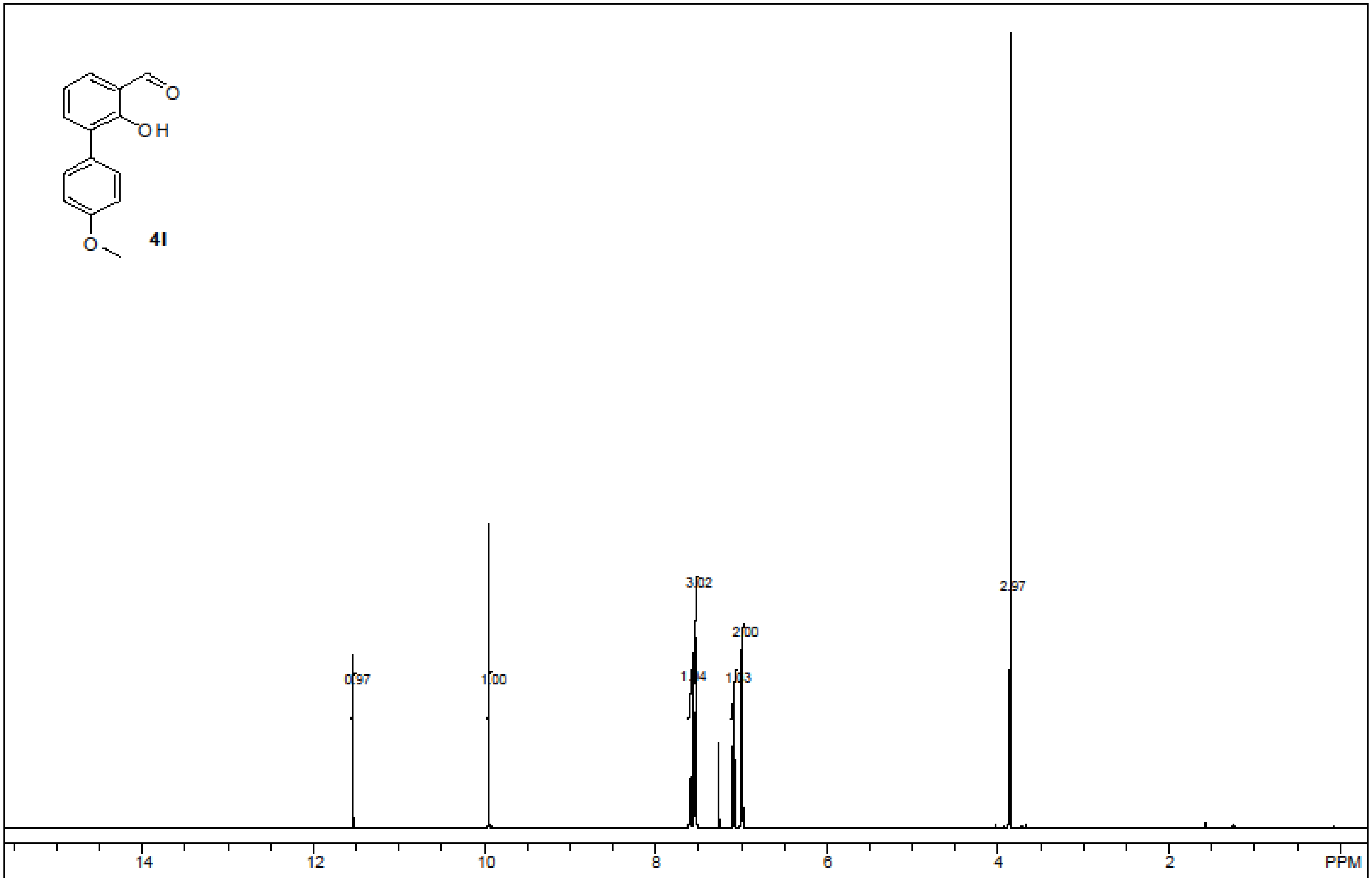

Avance, CDCl3, USER: nmrsu -- DATE: Wed Jan 20 15:05:51 2021

F1: 399.956	F2: 1.000	SW1: 7813		OF1: 2460.1		PTS1d: 65536	
EX: zg30		PW: 12.0 us	PD: 1.0 sec	NA: 16	LB: 0.0		Nuts - $pdata

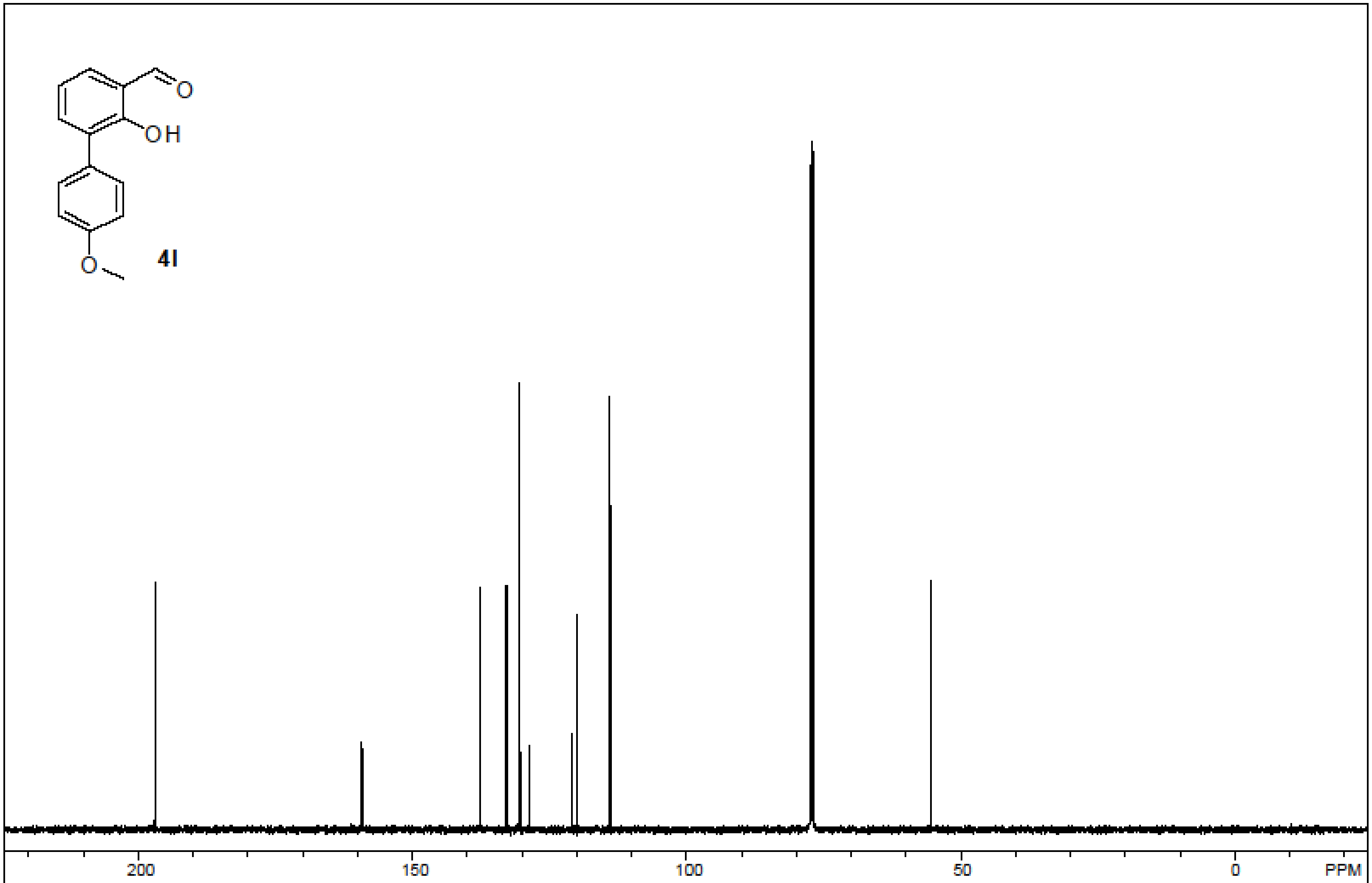

Avance, CDCl3,						USER: nmrsu -- DATE: Wed Jan 20 15:18:24 2021	
F1: 100.579	F2: 1.000	SW1: 25000		OF1: 10069.2		PTS1d: 32768	
EX: zgpg30		PW: 10.0 us	PD: 2.0 sec	NA: 256	LB: 0.0		Nuts - $pdata

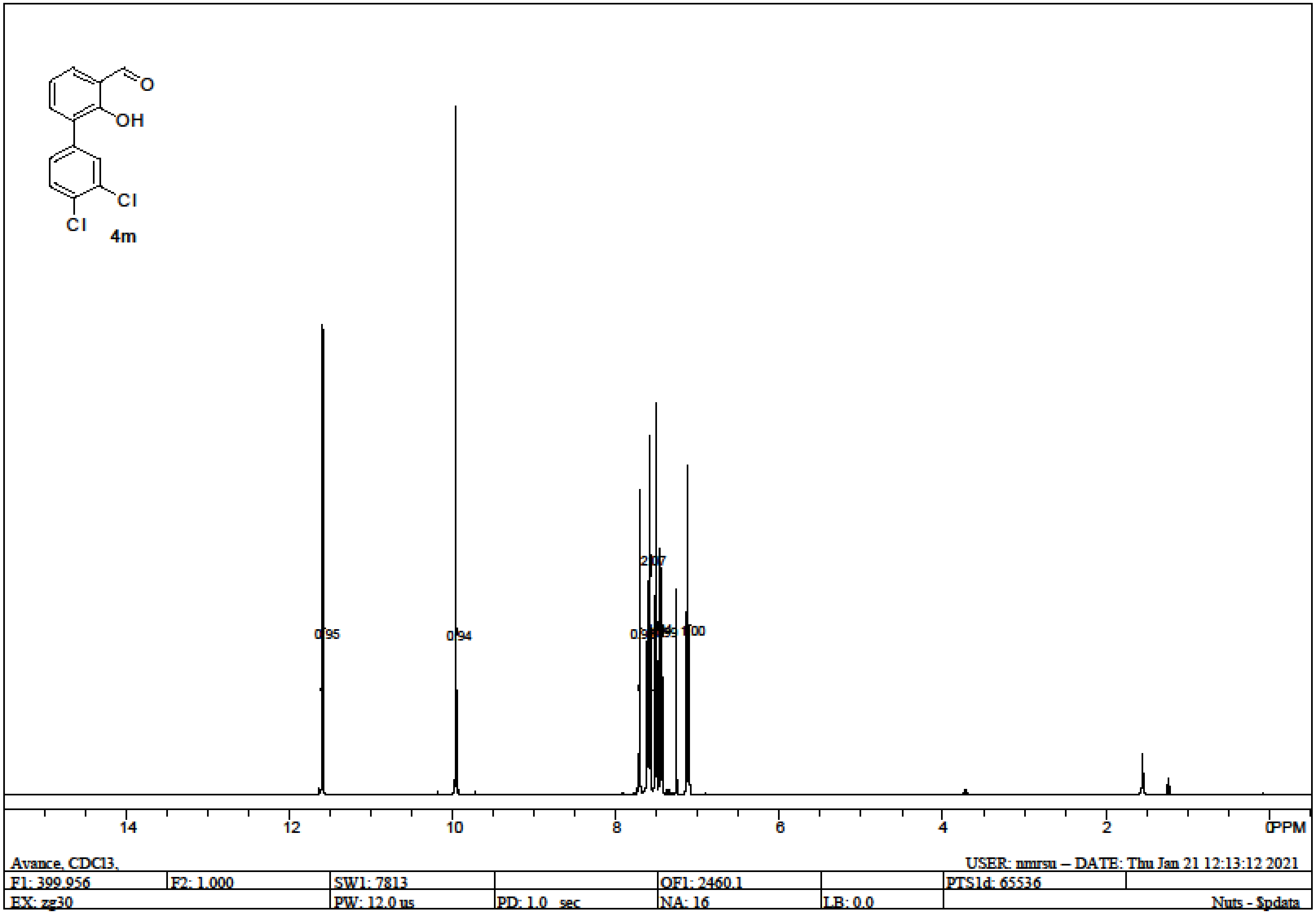

O
OH
Cl
Cl
4m
0.95
0.94
2.07
1.00
14
12
10
8
6
4
2
0PPM
Avance, CDCl3,
USER: nmrsu -- DATE: Thu Jan 21 12:13:12 2021
F1: 399.956
F2: 1.000
SW1: 7813
OF1: 2460.1
PTS1d: 65536
EX: zg30
PW: 12.0 us
PD: 1.0 sec
NA: 16
LB: 0.0
Nuts - $pdata

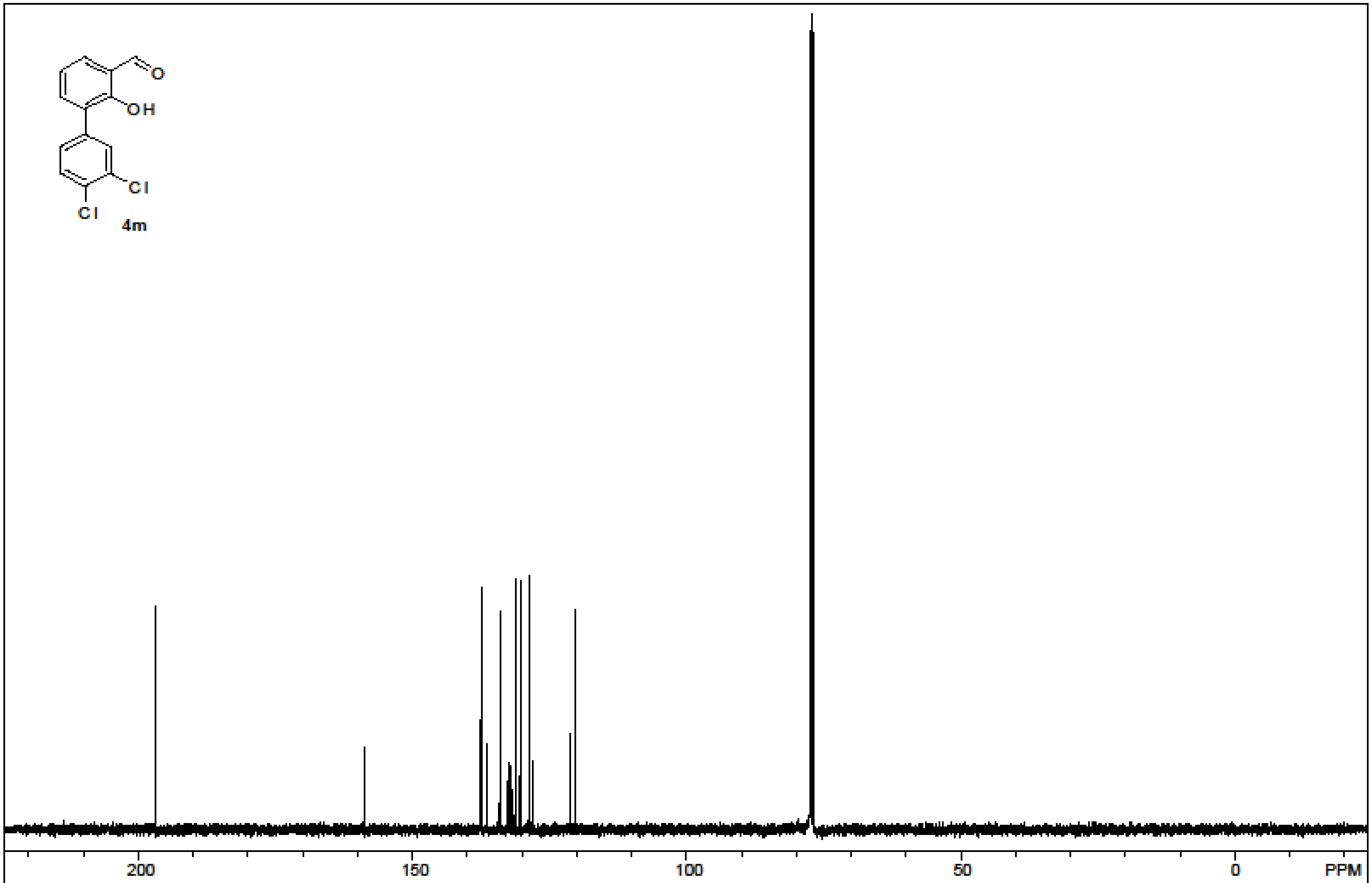

Avance, CDCl3, USER: nmrsu -- DATE: Thu Jan 21 12:20:50 2021

F1: 100.579	F2: 1.000	SW1: 25000		OF1: 10069.7		PTS1d: 32768	
EX: zgpg30		PW: 10.0 us	PD: 2.0 sec	NA: 135	LB: 0.0	Nuts - $pdata	

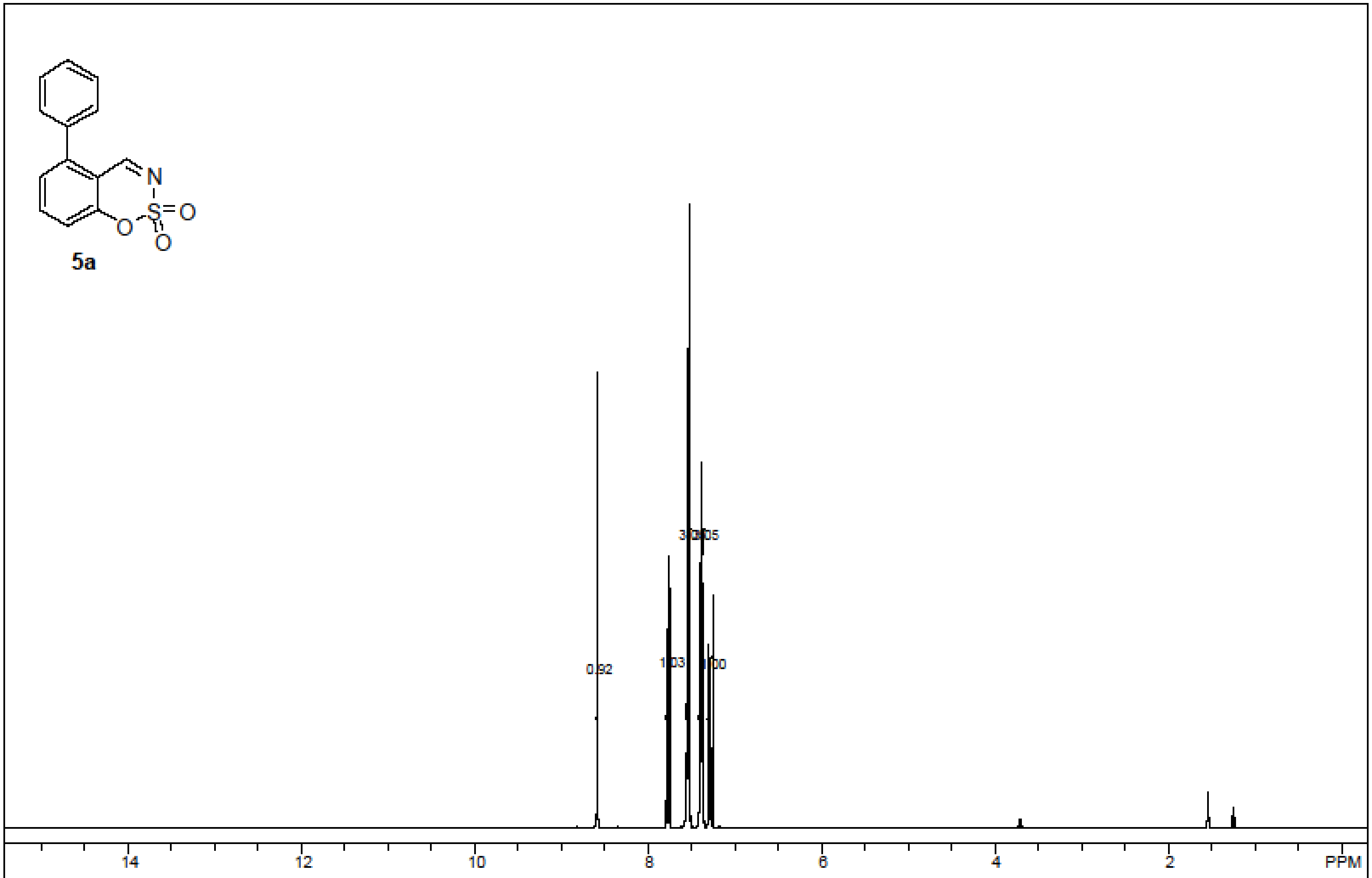

Avance, CDCl3, USER: nmrsu -- DATE: Fri Oct 23 15:41:02 2020

F1: 399.956	F2: 1.000	SW1: 7813		OF1: 2460.1		PTS1d: 65536	
EX: zg30		PW: 12.0 us	PD: 1.0 sec	NA: 16	LB: 0.0	Nuts - $pdata	

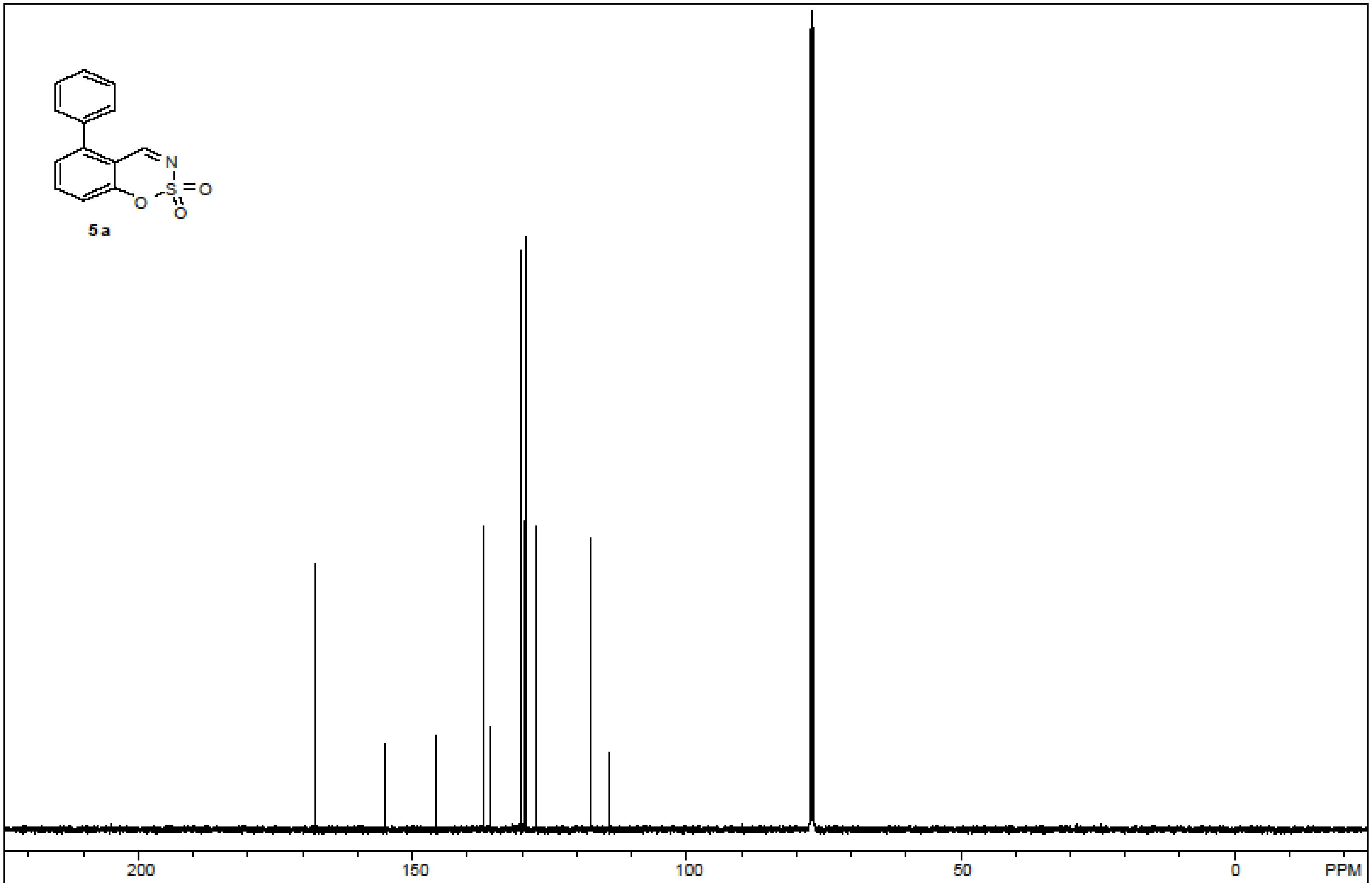

Avance, CDCl3, USER: nmrsu -- DATE: Fri Oct 23 15:59:24 2020

F1: 100.579	F2: 1.000	SW1: 25000		OF1: 10069.0		PTS1d: 32768	
EX: zgpg30		PW: 10.0 us	PD: 2.0 sec	NA: 350	LB: 0.0	Nuts - $pdata	

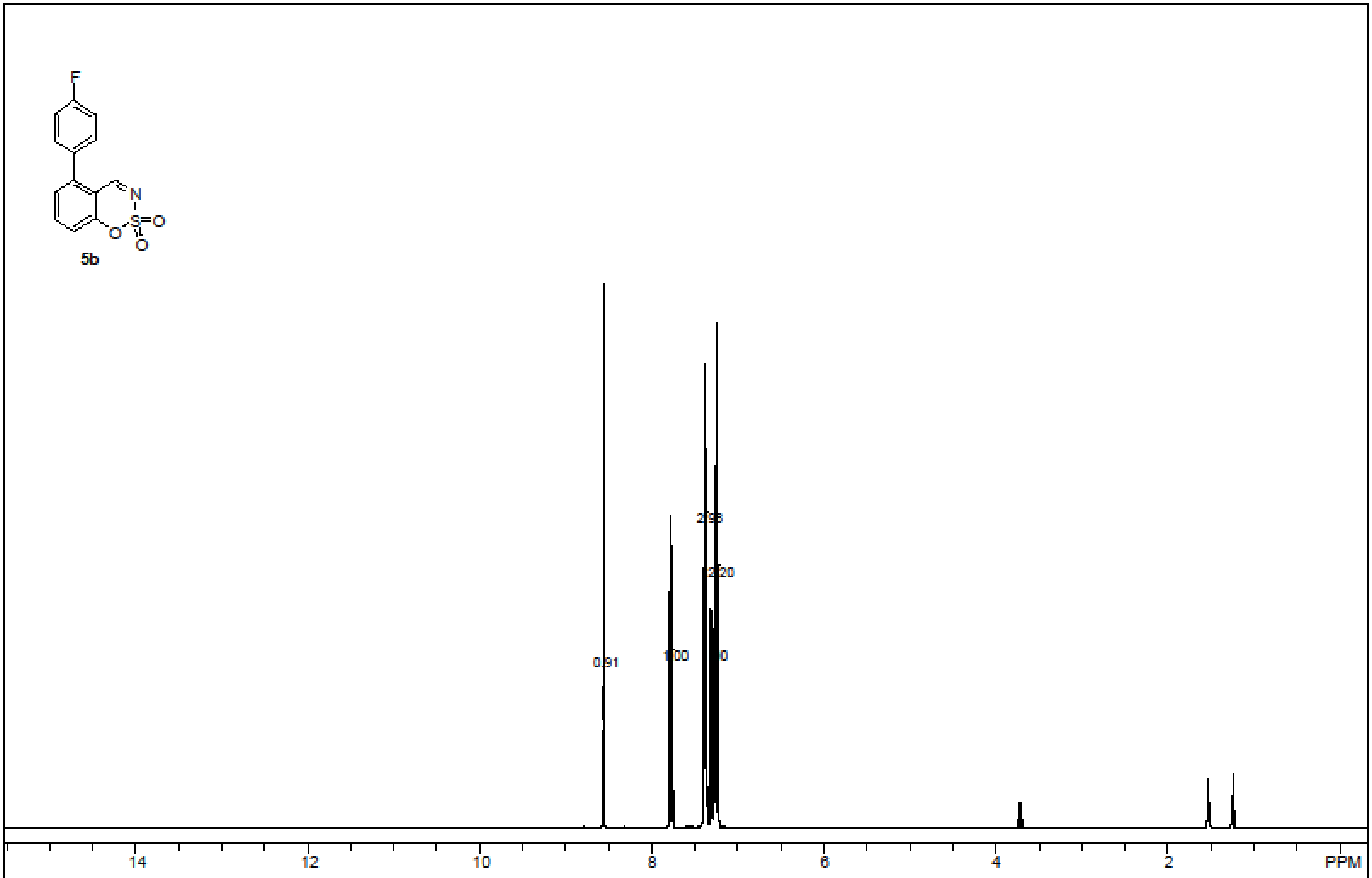

Avance, CDCl3,

USER: nmrsu -- DATE: Fri Oct 23 17:27:42 2020

F1: 399.956	F2: 1.000	SW1: 7813		OF1: 2460.5		PTS1d: 65536	
EX: zg30		PW: 12.0 us	PD: 1.0 sec	NA: 16	LB: 0.0		Nuts - $pdata

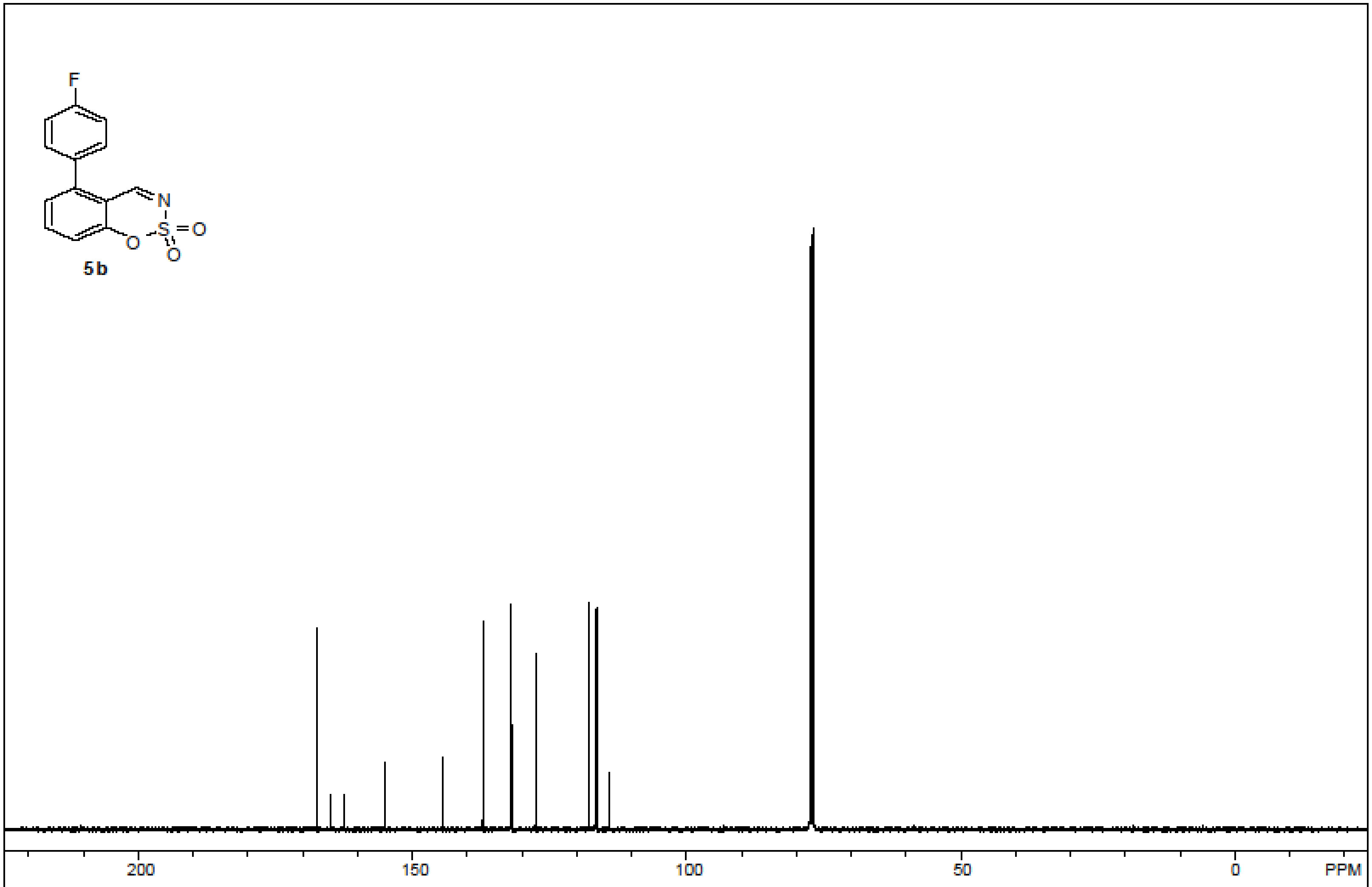

Avance, CDCl3,						USER: nmrsu -- DATE: Fri Oct 23 17:52:03 2020	
F1: 100.579	F2: 1.000	SW1: 25000		OF1: 10069.7		PTS1d: 32768	
EX: zgpg30		PW: 10.0 us	PD: 2.0 sec	NA: 512	LB: 0.0	Nuts - $pdata	

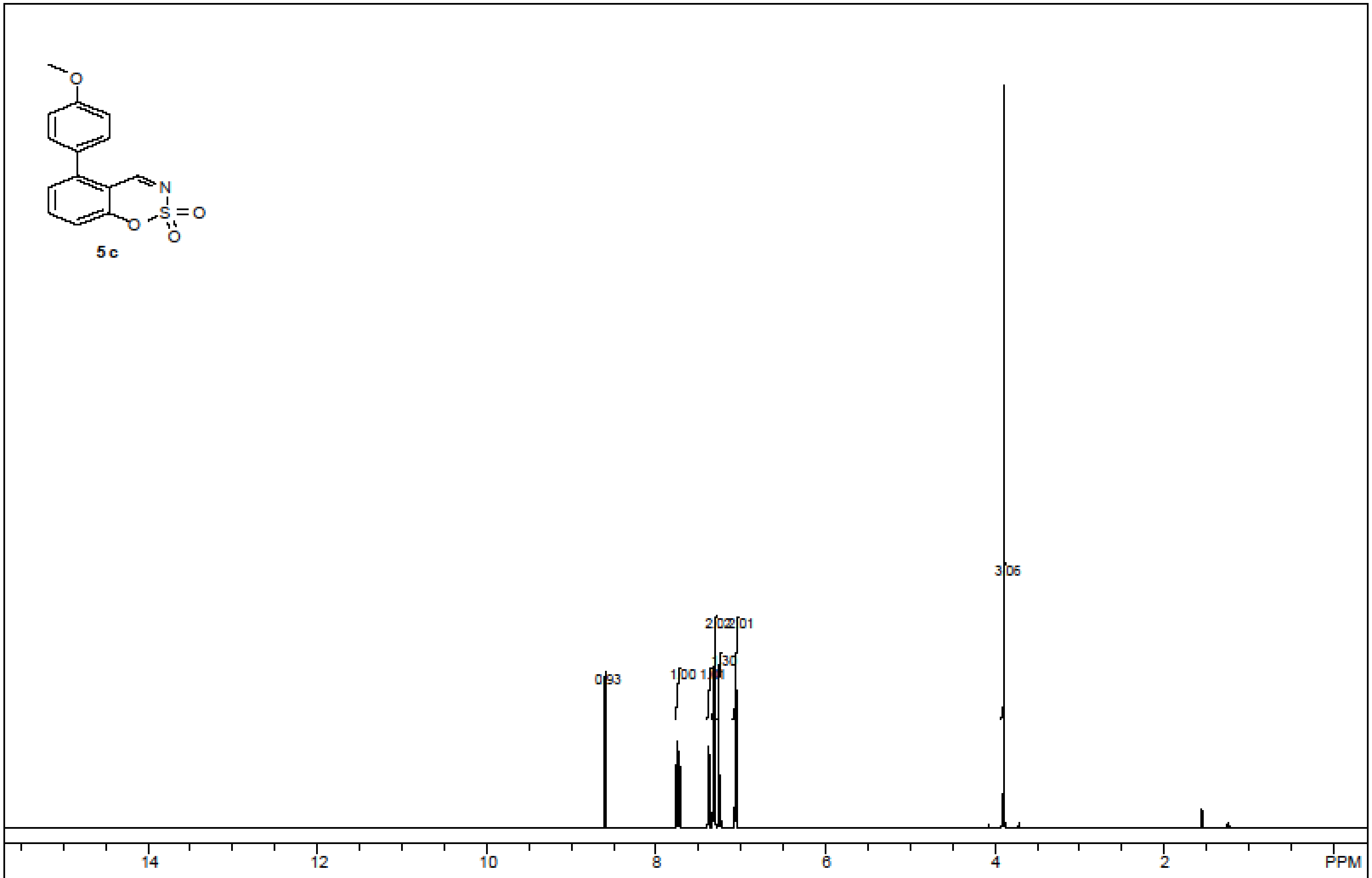

Avance, CDCl3, USER: nmrsu -- DATE: Fri Oct 23 16:57:58 2020

F1: 399.956	F2: 1.000	SW1: 7813		OF1: 2460.1		PTS1d: 65536	
EX: zg30		PW: 12.0 us	PD: 1.0 sec	NA: 16	LB: 0.0		Nuts - $pdata

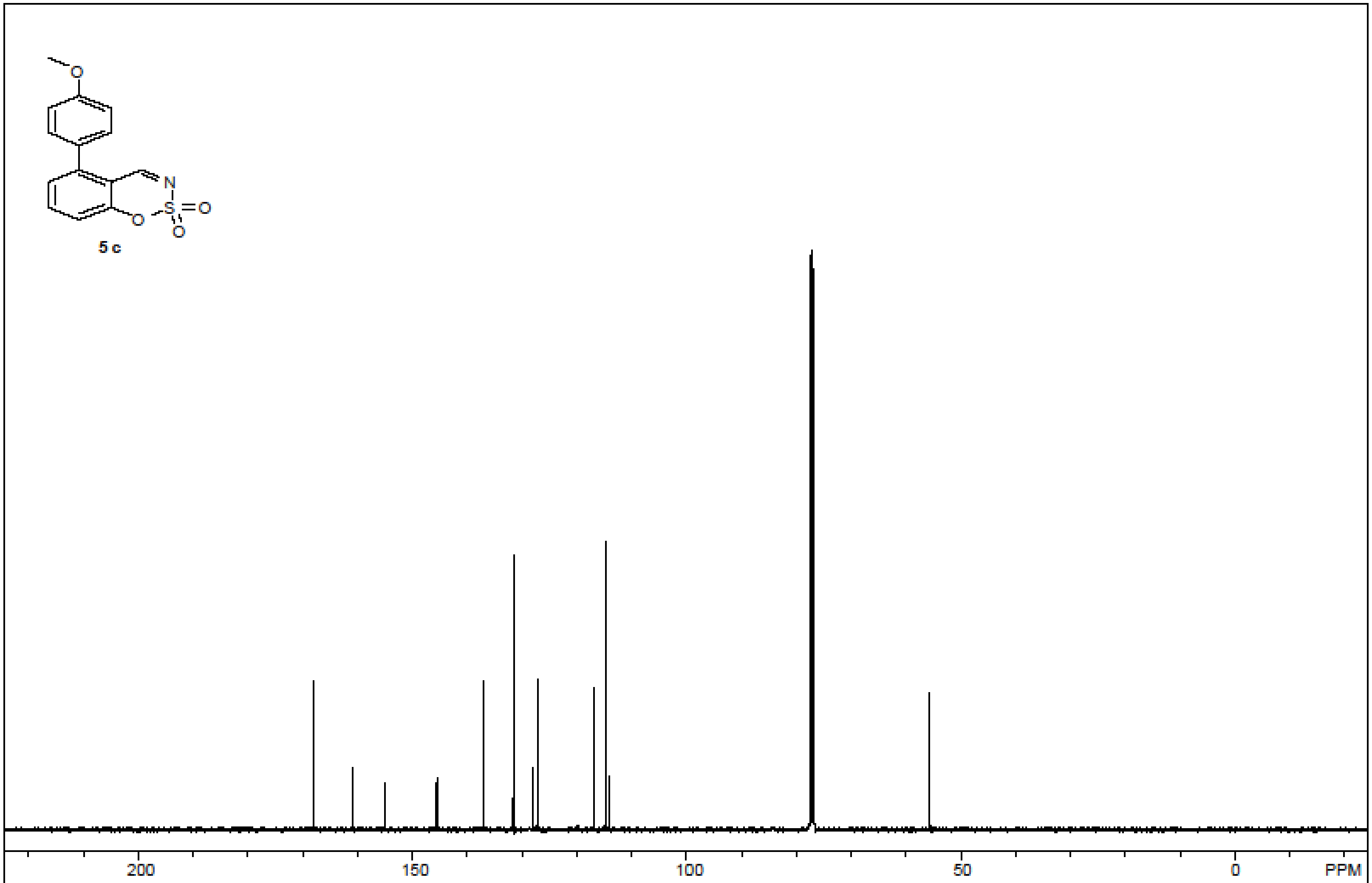

Avance, CDCl3, USER: nmrsu -- DATE: Fri Oct 23 17:22:52 2020

F1: 100.579	F2: 1.000	SW1: 25000		OF1: 10070.1		PTS1d: 32768	
EX: zgpg30		PW: 10.0 us	PD: 2.0 sec	NA: 512	LB: 0.0		Nuts - $pdata

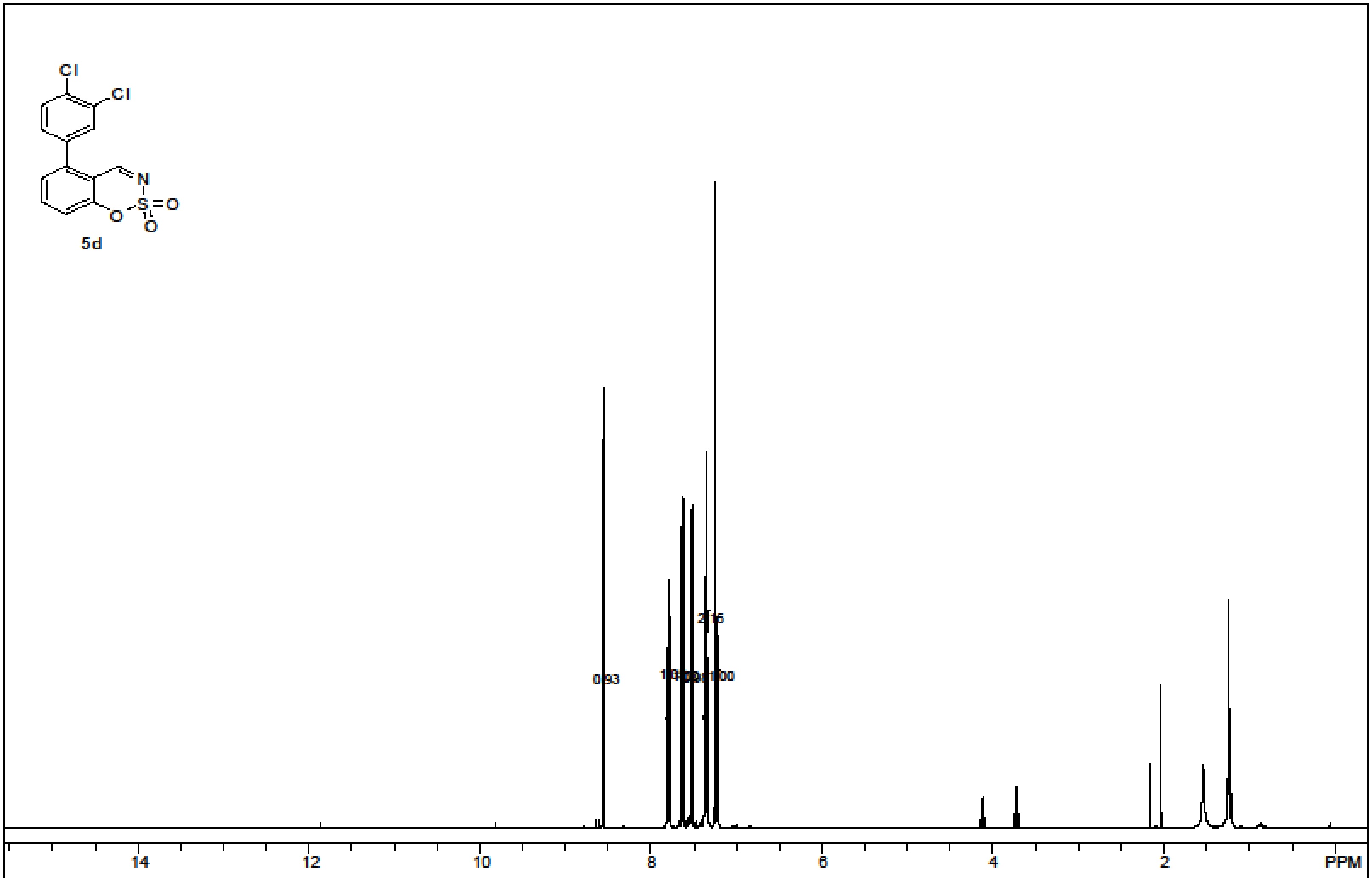

Avance, CDCl3, USER: nmrsu -- DATE: Wed Nov 11 12:22:23 2020

F1: 399.956	F2: 1.000	SW1: 7813		OF1: 2460.0		PTS1d: 65536	
EX: zg30		PW: 12.0 us	PD: 1.0 sec	NA: 16	LB: 0.0		Nuts - $pdata

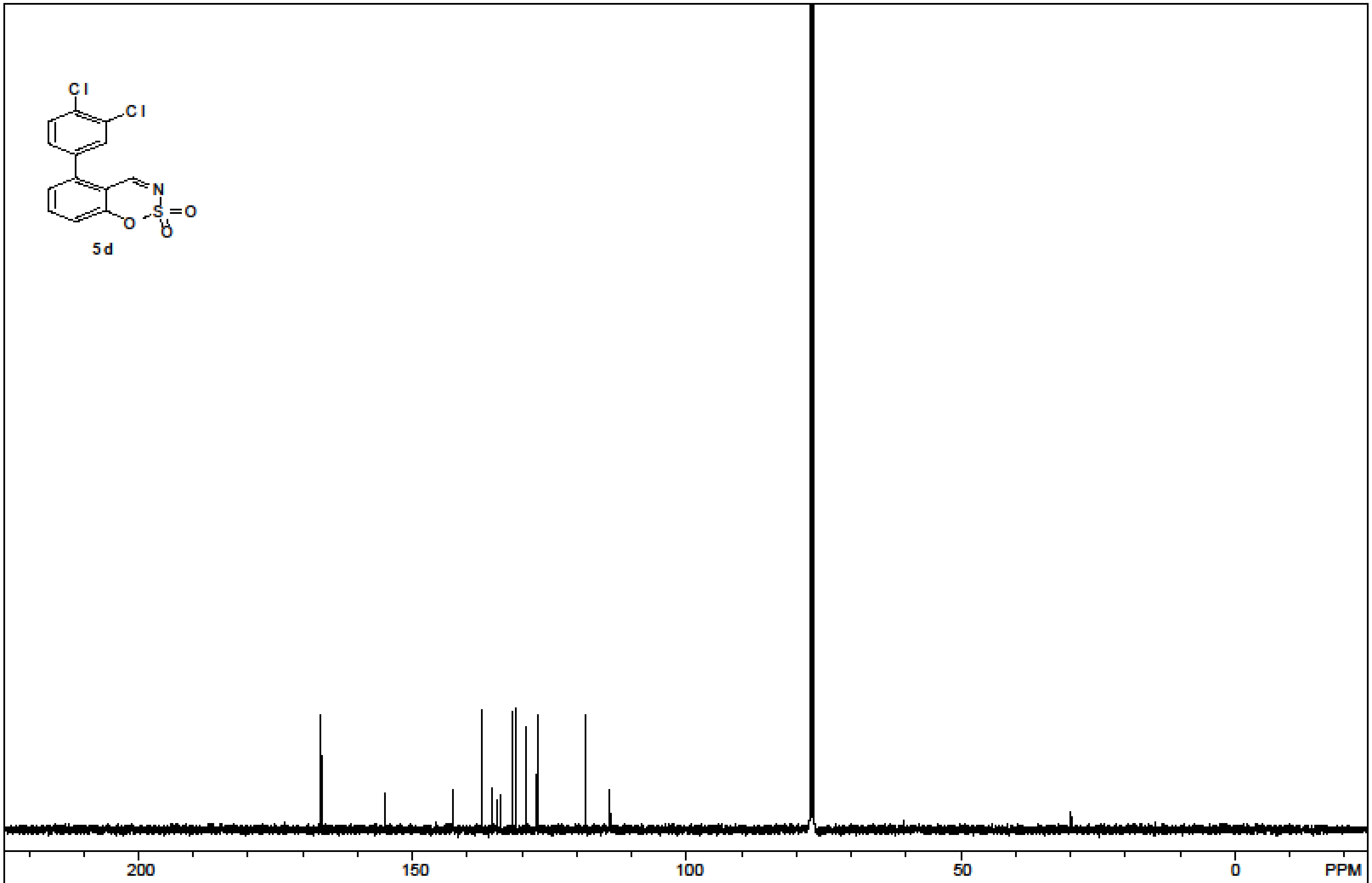

Avance, CDCl3,

USER: nmrsu -- DATE: Wed Nov 11 12:35:31 2020

F1: 100.579	F2: 1.000	SW1: 25000		OF1: 10070.6		PTS1d: 32768	
EX: zgpg30		PW: 10.0 us	PD: 2.0 sec	NA: 256	LB: 0.0	Nuts - $pdata	

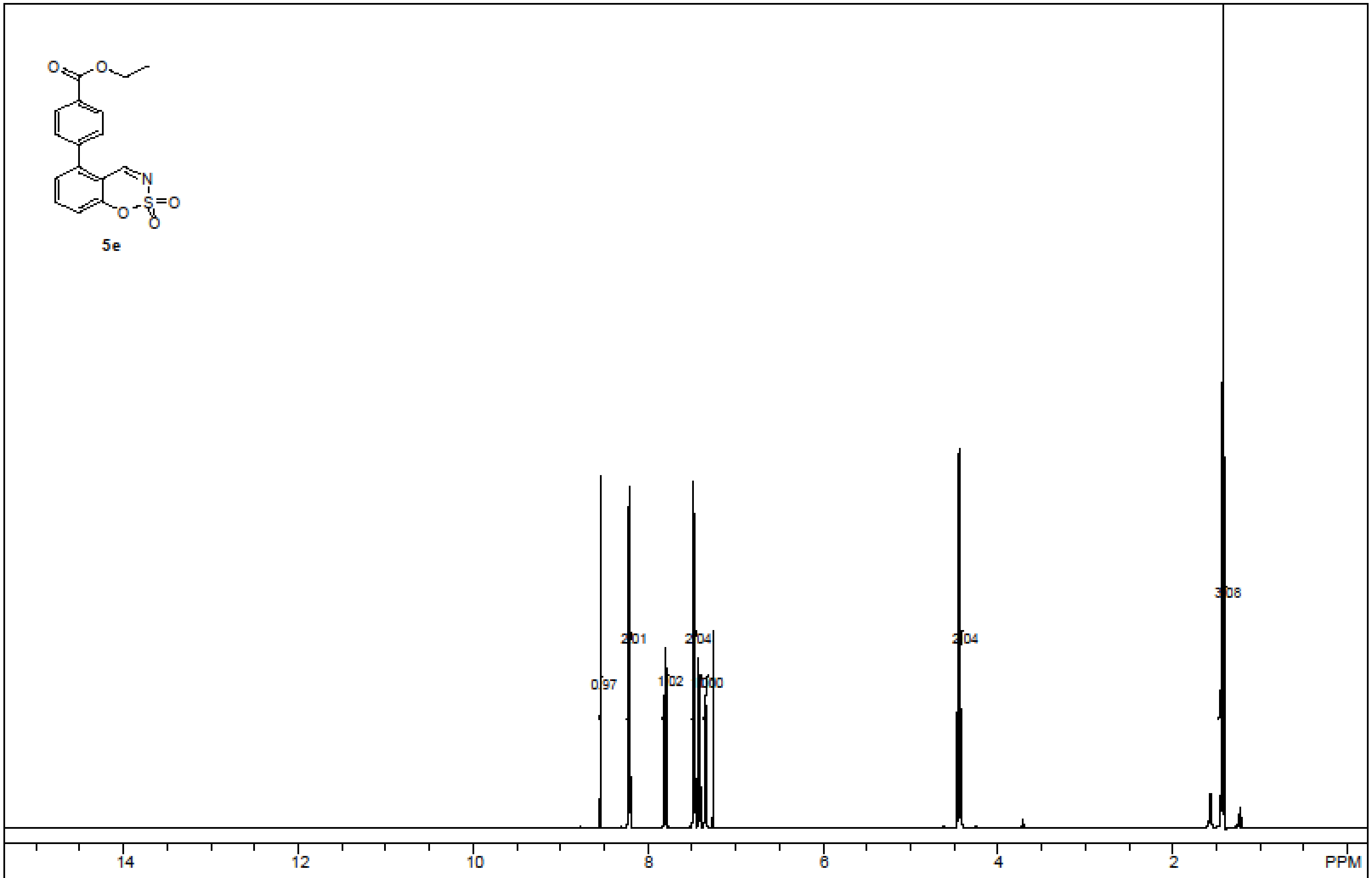

Avance, CDCl3,

USER: nmrsu -- DATE: Wed Nov 11 12:45:50 2020

F1: 399.956	F2: 1.000	SW1: 7813		OF1: 2460.0		PTS1d: 65536	
EX: zg30		PW: 12.0 us	PD: 1.0 sec	NA: 16	LB: 0.0		Nuts - $pdata

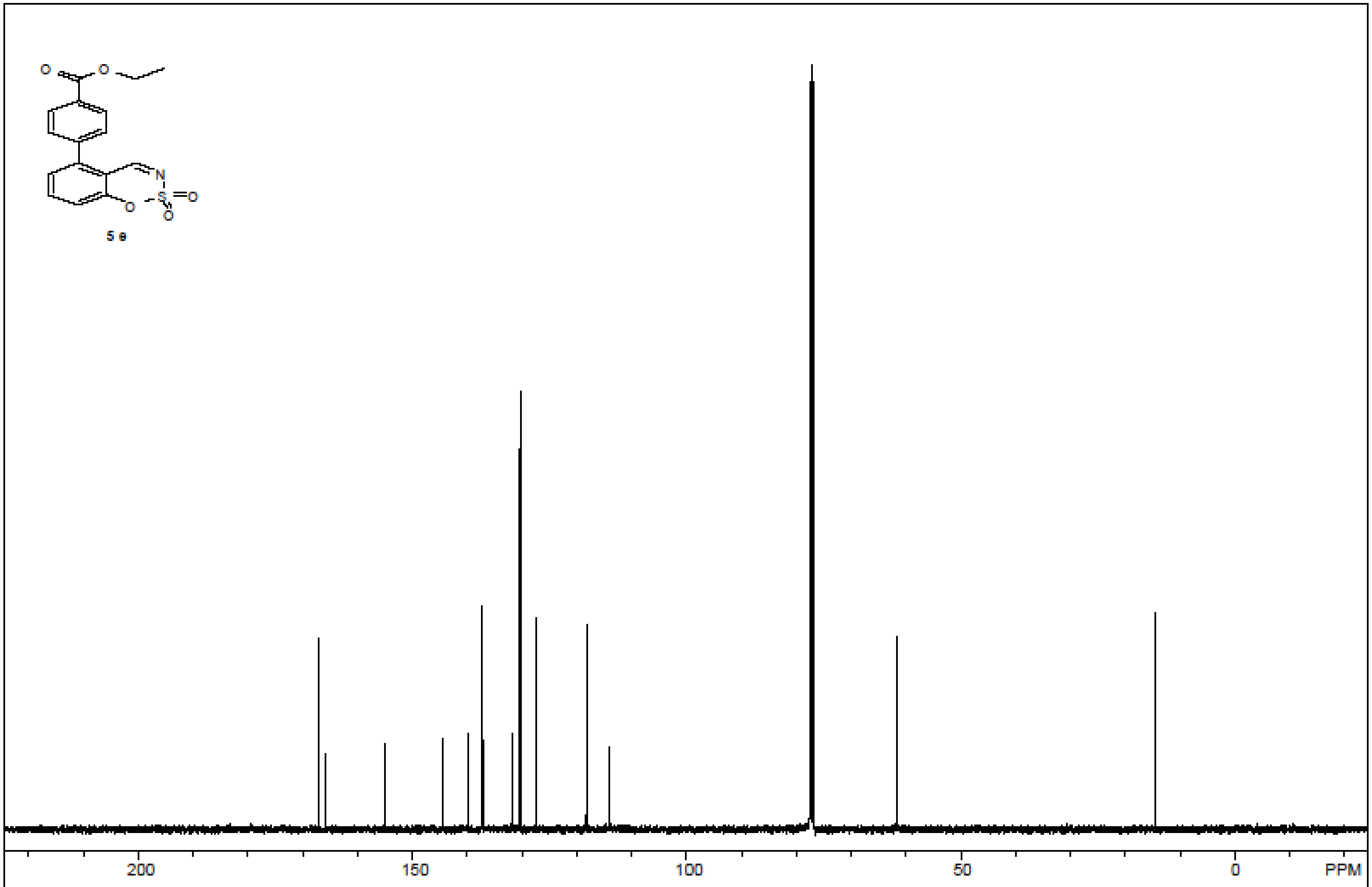

Avance, CDCl3, USER: nmrsu -- DATE: Wed Nov 11 12:58:24 2020

F1: 100.579	F2: 1.000	SW1: 25000		OF1: 10069.0		PTS1d: 32768	
EX: zgpg30		PW: 10.0 us	PD: 2.0 sec	NA: 256	LB: 0.0	Nuts - $pdata	

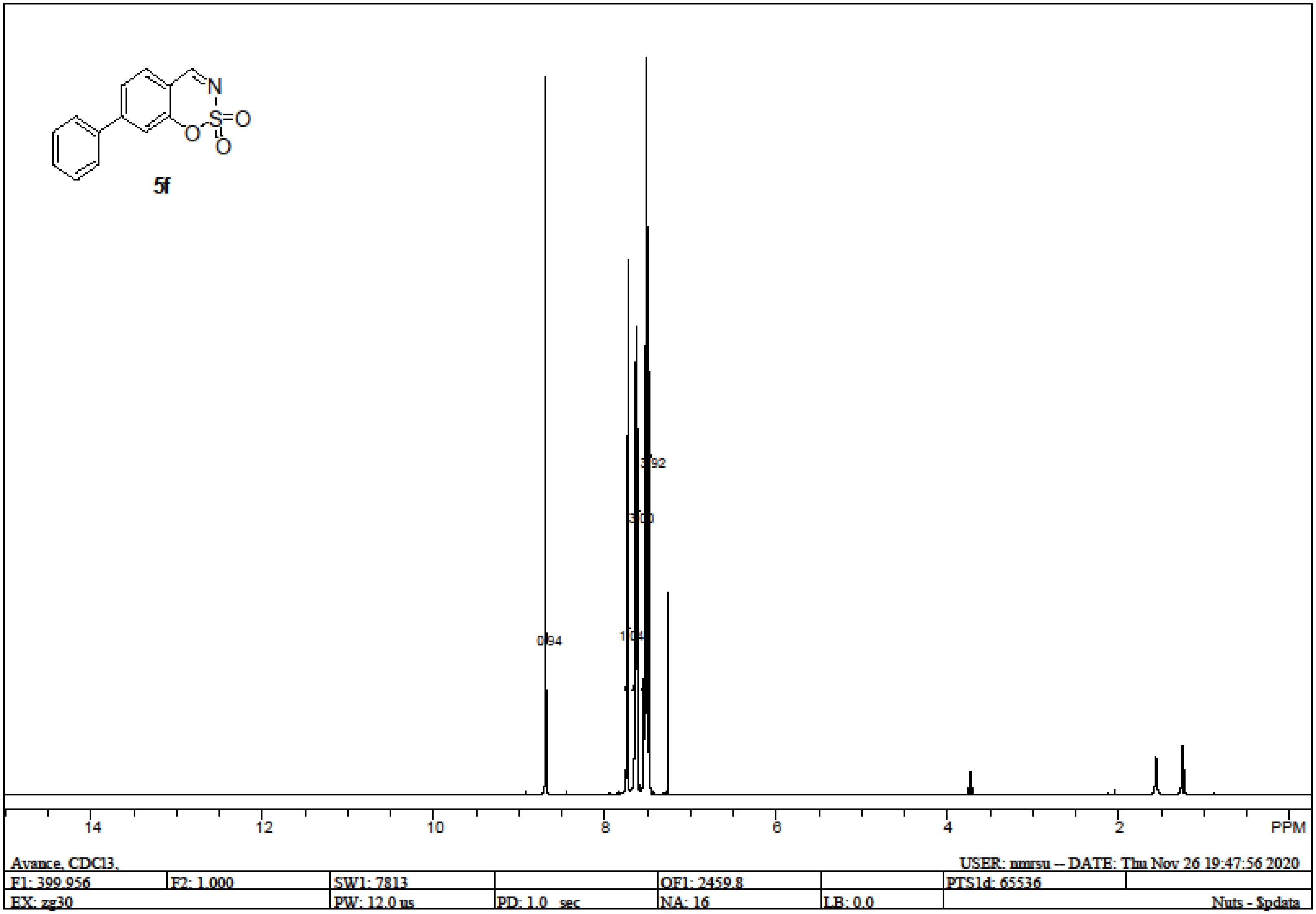
N
S
O
O
O
5f
0.94
1.04
3.00
3.92
14
12
10
8
6
4
2
PPM
Avance, CDCl3,
USER: nmrsu -- DATE: Thu Nov 26 19:47:56 2020
F1: 399.956
F2: 1.000
SW1: 7813
OF1: 2459.8
PTS1d: 65536
EX: zg30
PW: 12.0 us
PD: 1.0 sec
NA: 16
LB: 0.0
Nuts - $pdata

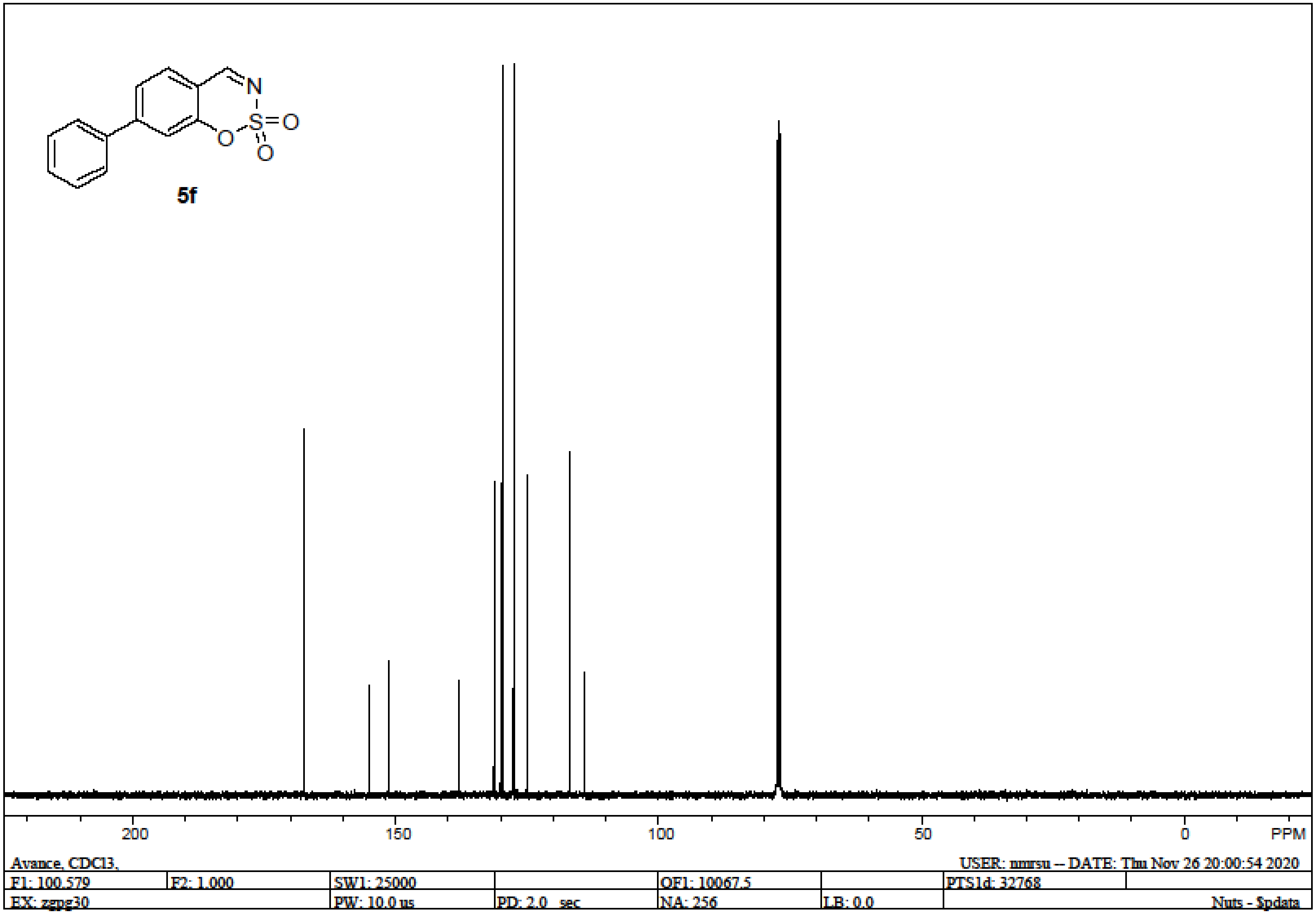

N
S
O
O
O
5f
200
150
100
50
0
PPM
Avance, CDCl3,
USER: nmrsu -- DATE: Thu Nov 26 20:00:54 2020
F1: 100.579
F2: 1.000
SW1: 25000
OF1: 10067.5
PTS1d: 32768
EX: zgpg30
PW: 10.0 us
PD: 2.0 sec
NA: 256
LB: 0.0
Nuts - $pdata

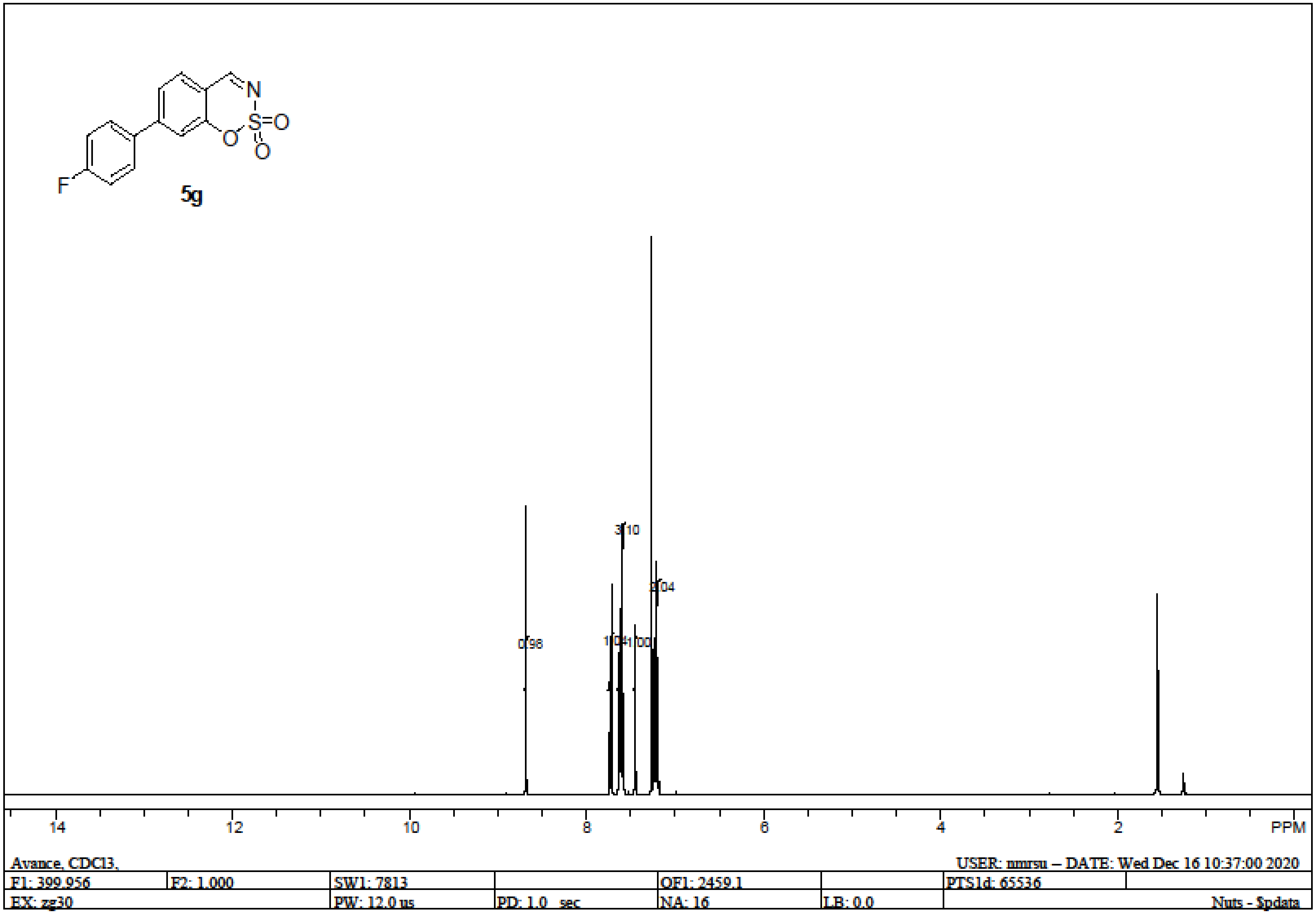

F
N
S
O
O
O
5g
0.98
1.04
3.10
1.00
2.04
14
12
10
8
6
4
2
PPM
Avance, CDCl3,
USER: nmrsu -- DATE: Wed Dec 16 10:37:00 2020
F1: 399.956
F2: 1.000
SW1: 7813
OF1: 2459.1
PTS1d: 65536
EX: zg30
PW: 12.0 us
PD: 1.0 sec
NA: 16
LB: 0.0
Nuts - $pdata

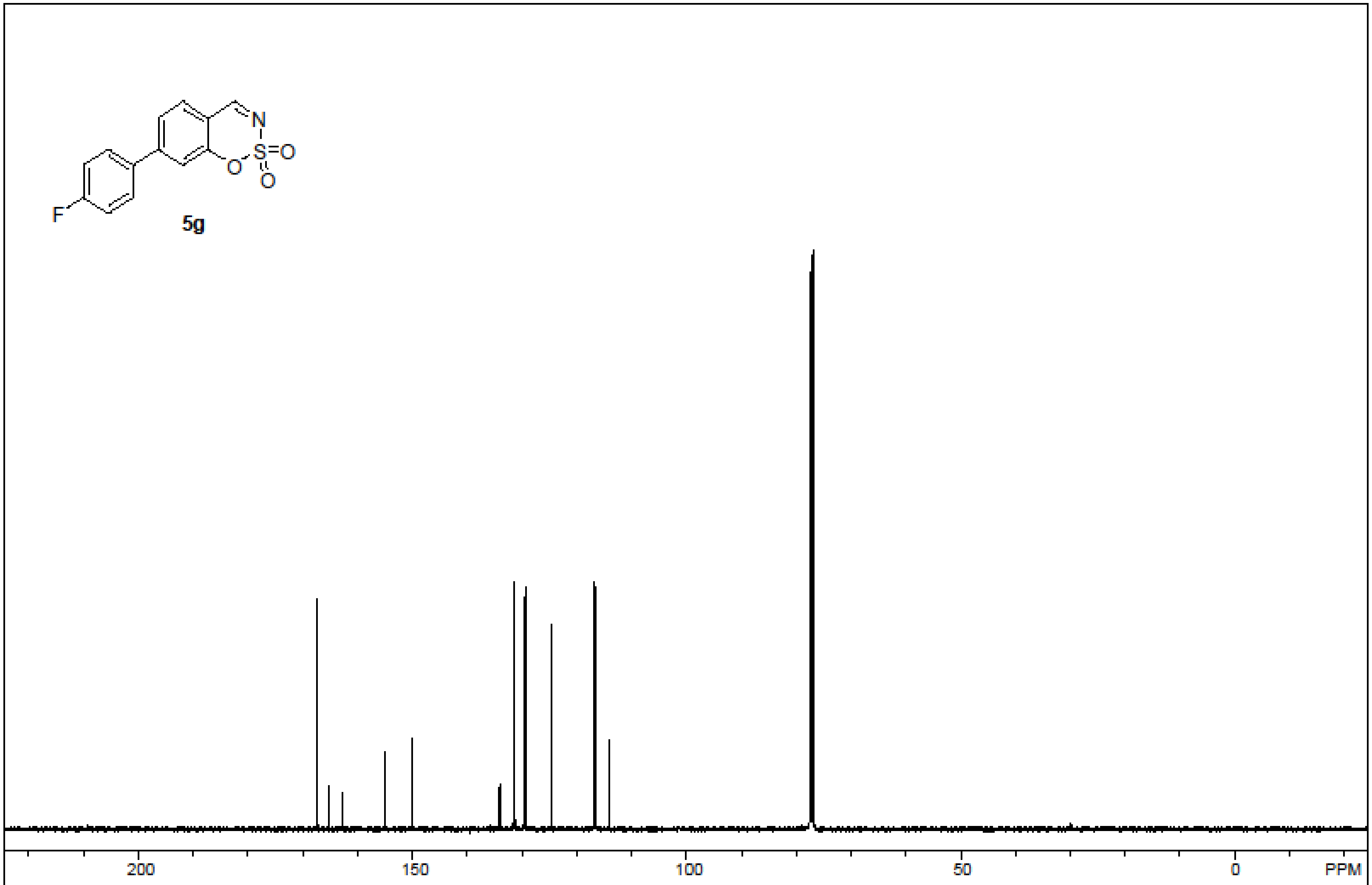

Avance, CDCl3, USER: nmrsu -- DATE: Thu Dec 17 15:33:24 2020

F1: 100.579	F2: 1.000	SW1: 25000		OF1: 10068.9		PTS1d: 32768	
EX: zgpg30		PW: 10.0 us	PD: 2.0 sec	NA: 512	LB: 0.0		Nuts - $pdata

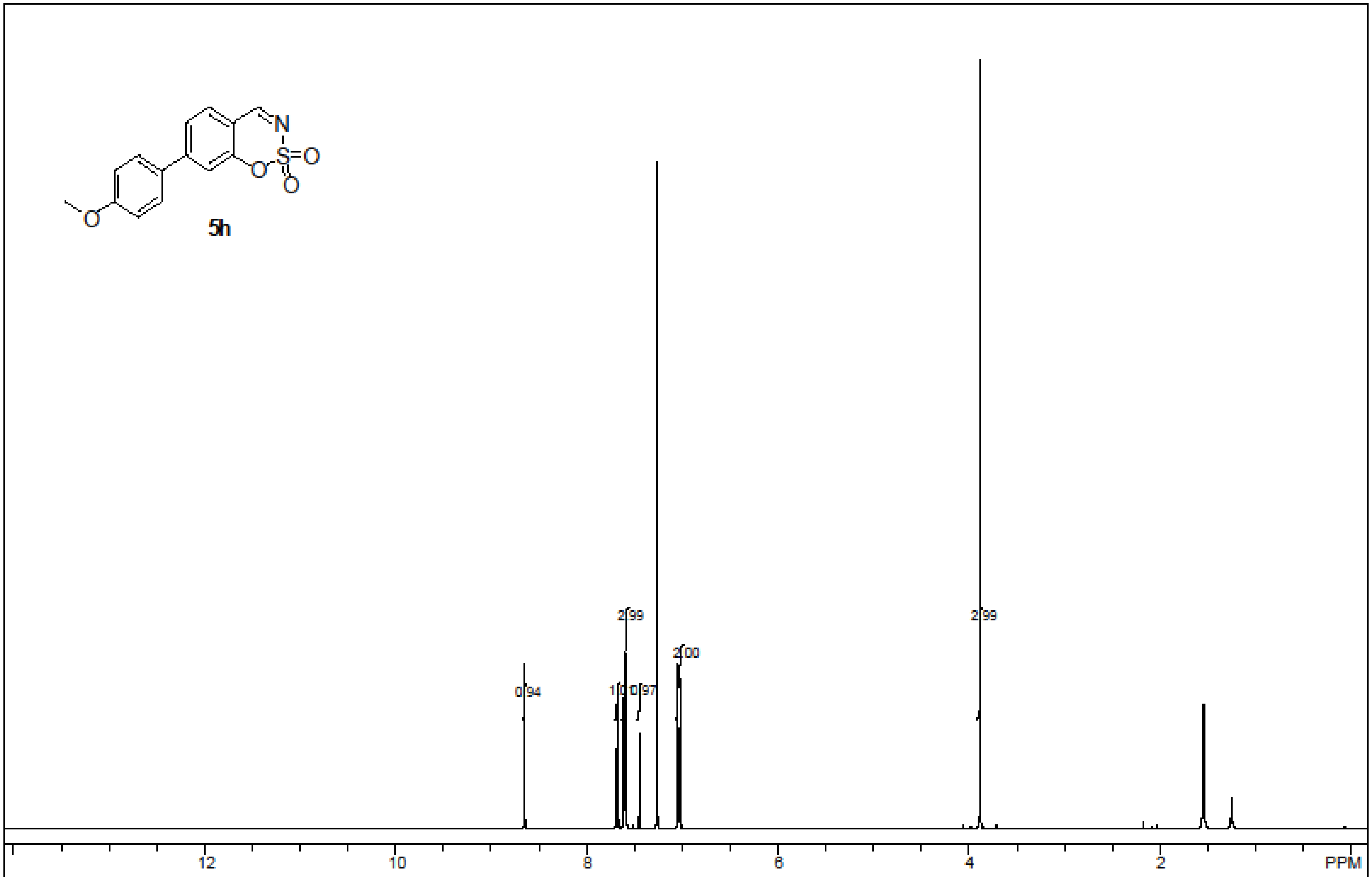

Avance, CDCl3,						USER: nmrsu -- DATE: Wed Dec 16 08:59:27 2020	
F1: 399.956	F2: 1.000	SW1: 7813		OF1: 2460.1		PTS1d: 65536	
EX: zg30		PW: 12.0 us	PD: 1.0 sec	NA: 16	LB: 0.0		Nuts - $pdata

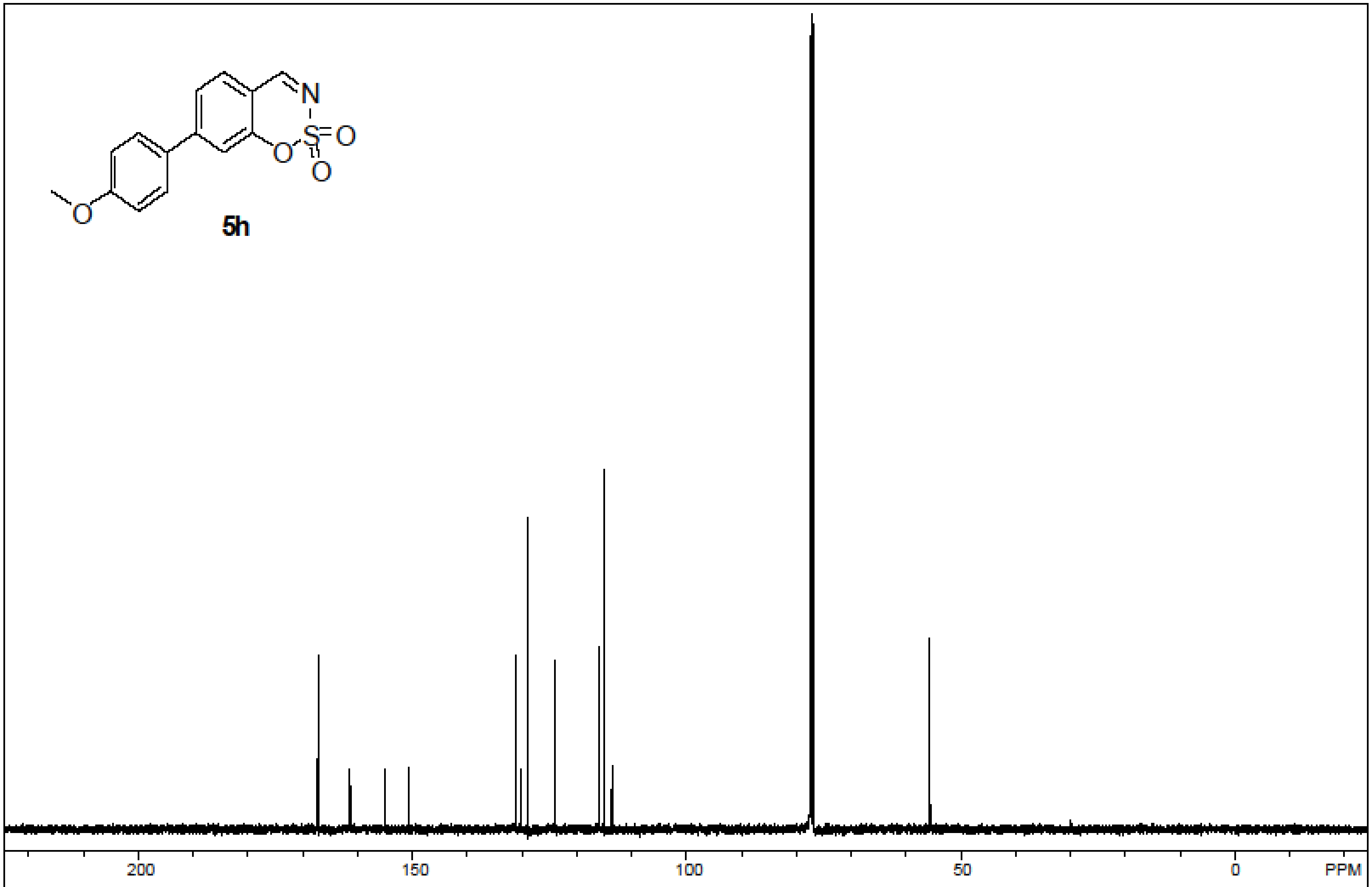

Avance, CDCl3,						USER: nmrsu -- DATE: Thu Dec 17 14:44:52 2020	
F1: 100.579	F2: 1.000	SW1: 25000		OF1: 10069.9		PTS1d: 32768	
EX: zgpg30		PW: 10.0 us	PD: 2.0 sec	NA: 240	LB: 0.0	Nuts - $pdata	

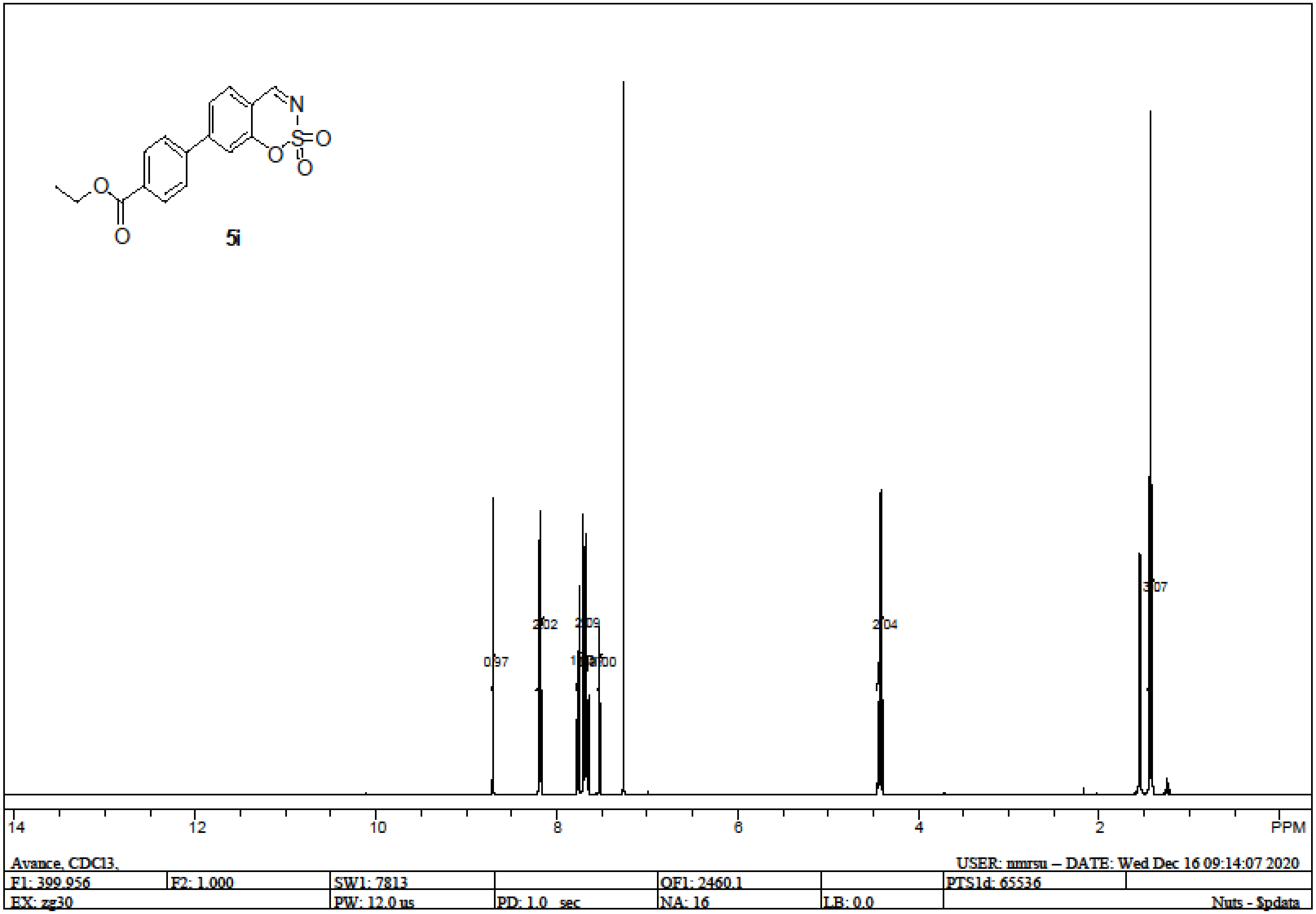

5i
0.97
2.02
2.09
2.04
3.07
14
12
10
8
6
4
2
PPM
Avance, CDCl3,
USER: nmrsu -- DATE: Wed Dec 16 09:14:07 2020
F1: 399.956
F2: 1.000
SW1: 7813
OF1: 2460.1
PTS1d: 65536
EX: zg30
PW: 12.0 us
PD: 1.0 sec
NA: 16
LB: 0.0
Nuts - $pdata

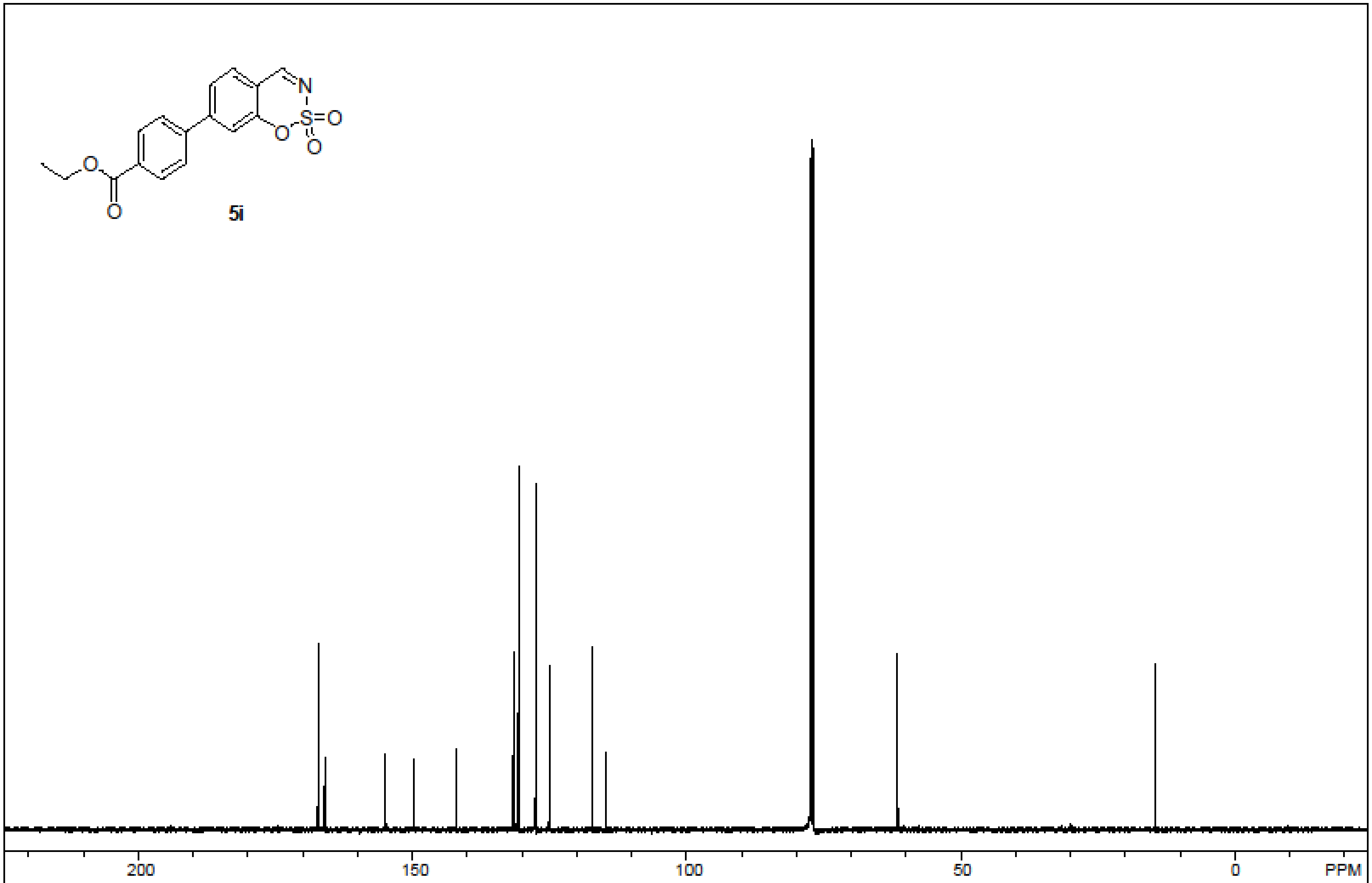

Avance, CDCl3, USER: nmrsu -- DATE: Thu Dec 17 16:38:37 2020

F1: 100.579	F2: 1.000	SW1: 25000		OF1: 10069.2		PTS1d: 32768	
EX: zgpg30		PW: 10.0 us	PD: 2.0 sec	NA: 512	LB: 0.0	Nuts - $pdata	

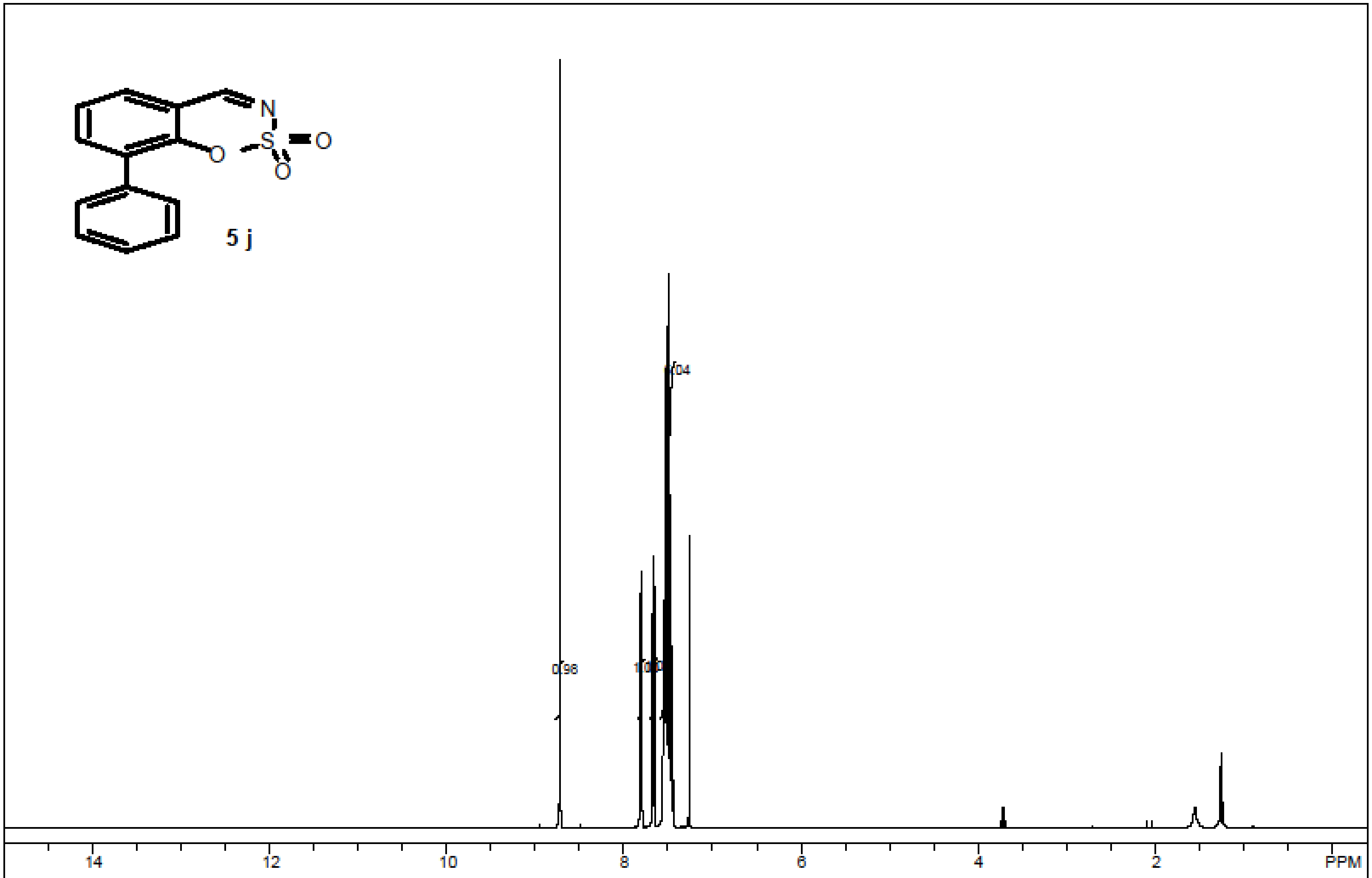

Avance, CDCl3, USER: nmrsu -- DATE: Mon Jan 11 11:21:31 2021

F1: 399.956	F2: 1.000	SW1: 7813		OF1: 2460.1		PTS1d: 65536	
EX: zg30		PW: 12.0 us	PD: 1.0 sec	NA: 16	LB: 0.0		Nuts - $pdata

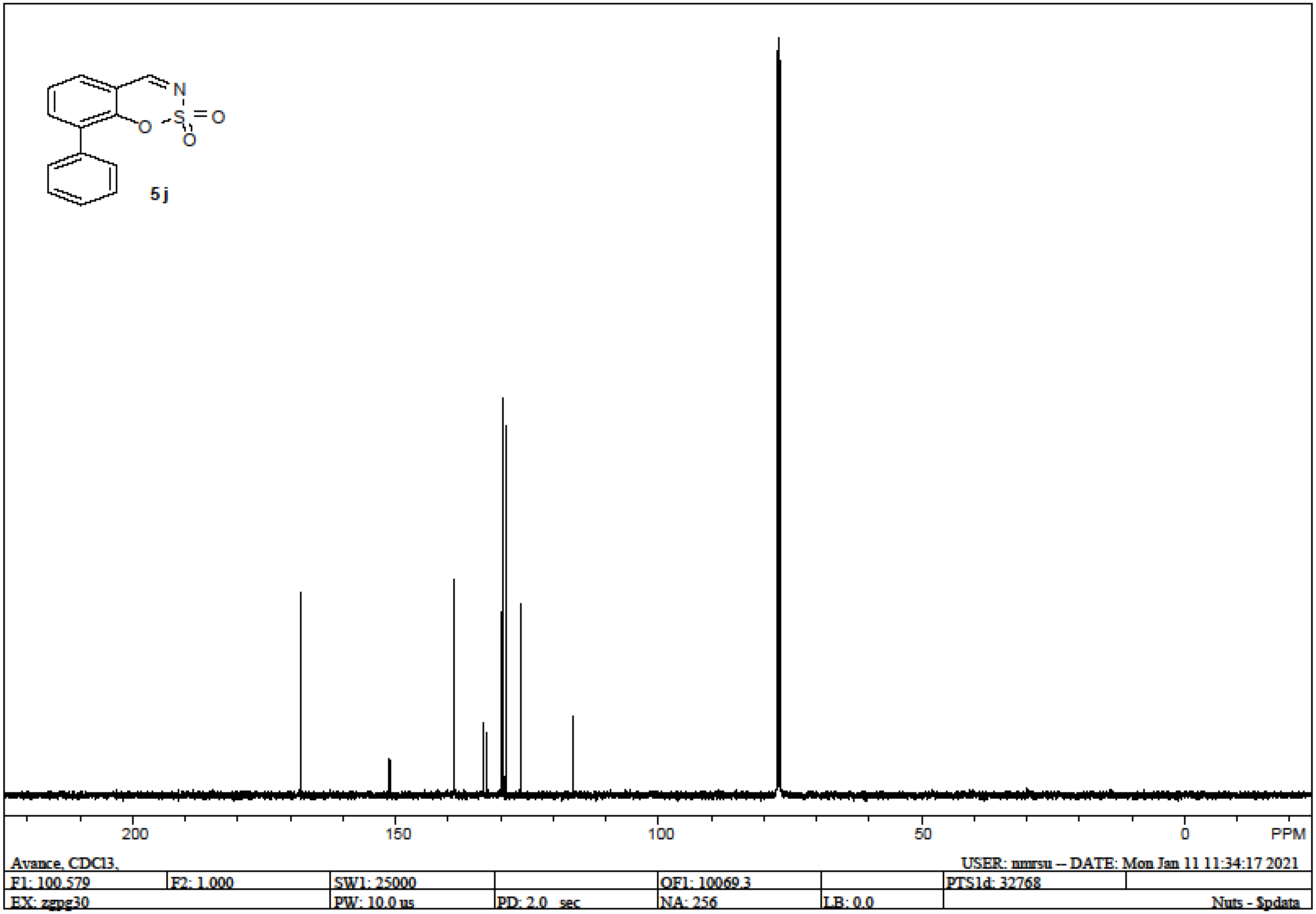
N
S
O
O
O
5j
200
150
100
50
0
PPM
Avance, CDCl3,
USER: nmrsu -- DATE: Mon Jan 11 11:34:17 2021
F1: 100.579
F2: 1.000
SW1: 25000
OF1: 10069.3
PTS1d: 32768
EX: zgpg30
PW: 10.0 us
PD: 2.0 sec
NA: 256
LB: 0.0
Nuts - $pdata

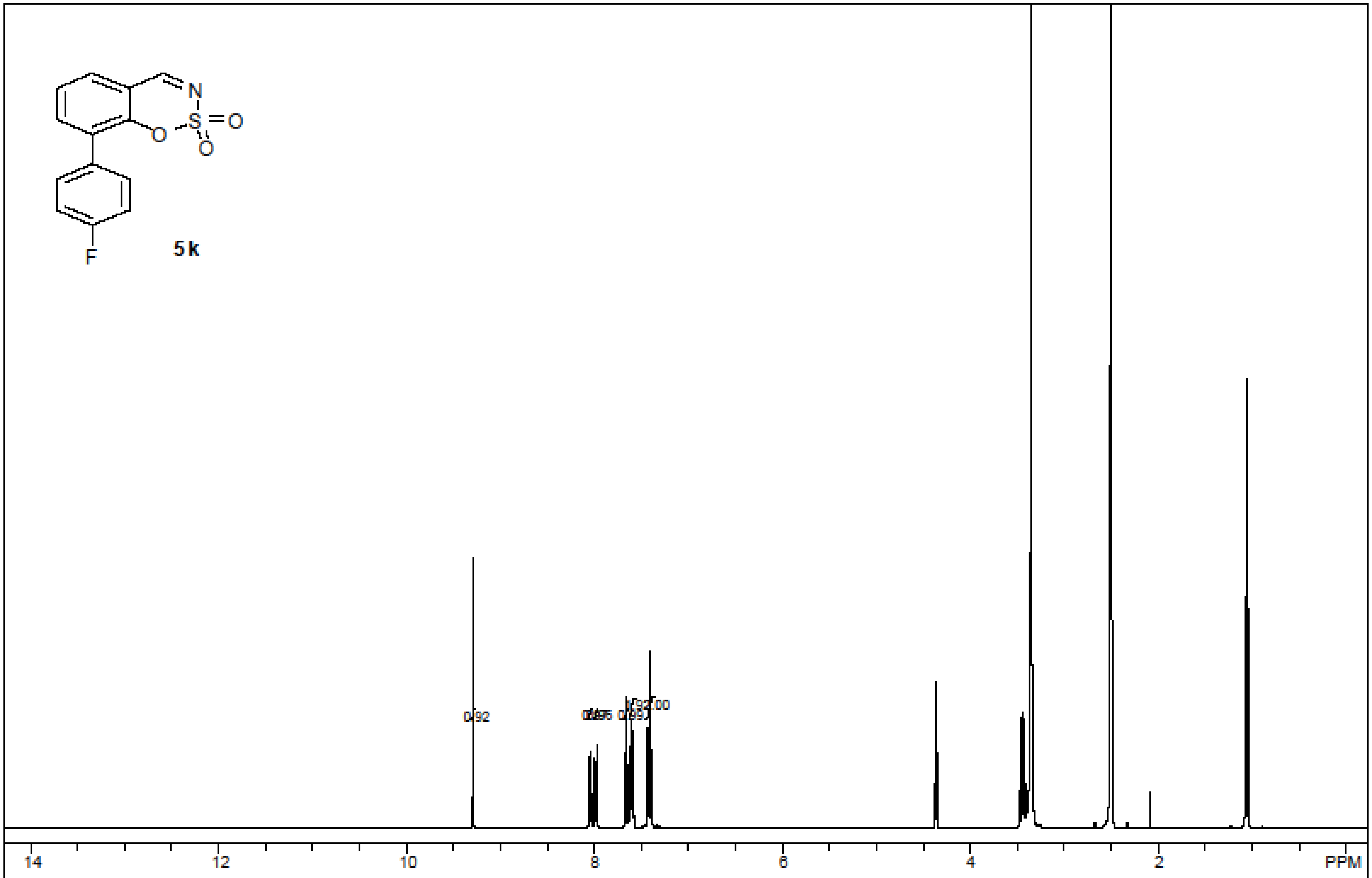

Avance, DMSO, USER: nmrsu -- DATE: Wed Jan 27 10:50:47 2021

F1: 399.956	F2: 1.000	SW1: 7813		OF1: 2466.5		PTS1d: 65536	
EX: zg30		PW: 12.0 us	PD: 1.0 sec	NA: 16	LB: 0.0		Nuts - $pdata

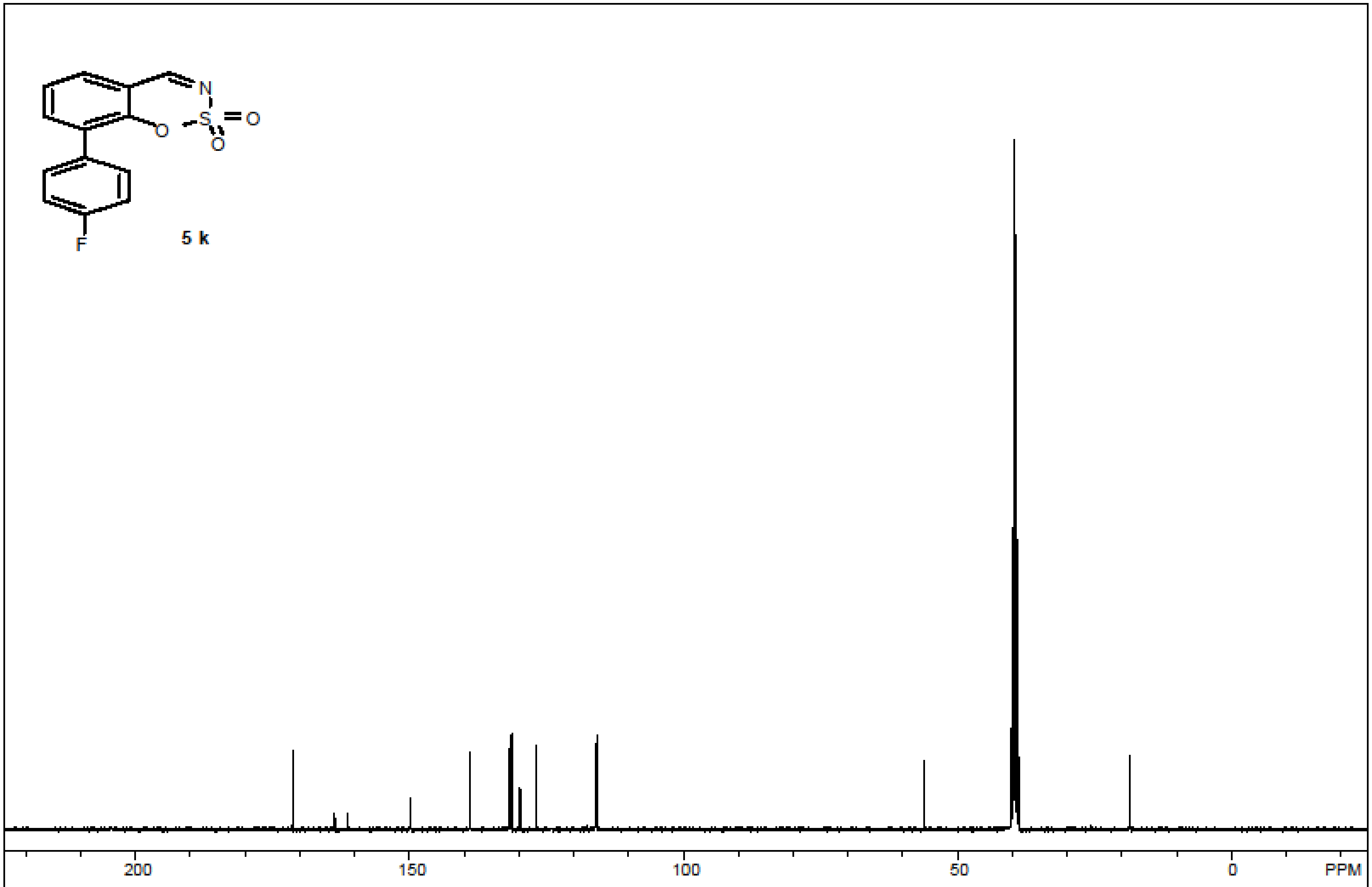

Avance, DMSO, USER: nmrsu -- DATE: Tue Feb 23 12:53:26 2021

F1: 100.579	F2: 1.000	SW1: 25000		OF1: 10012.8		PTS1d: 32768	
EX: zgpg30		PW: 10.0 us	PD: 2.0 sec	NA: 256	LB: 0.0		Nuts - $pdata

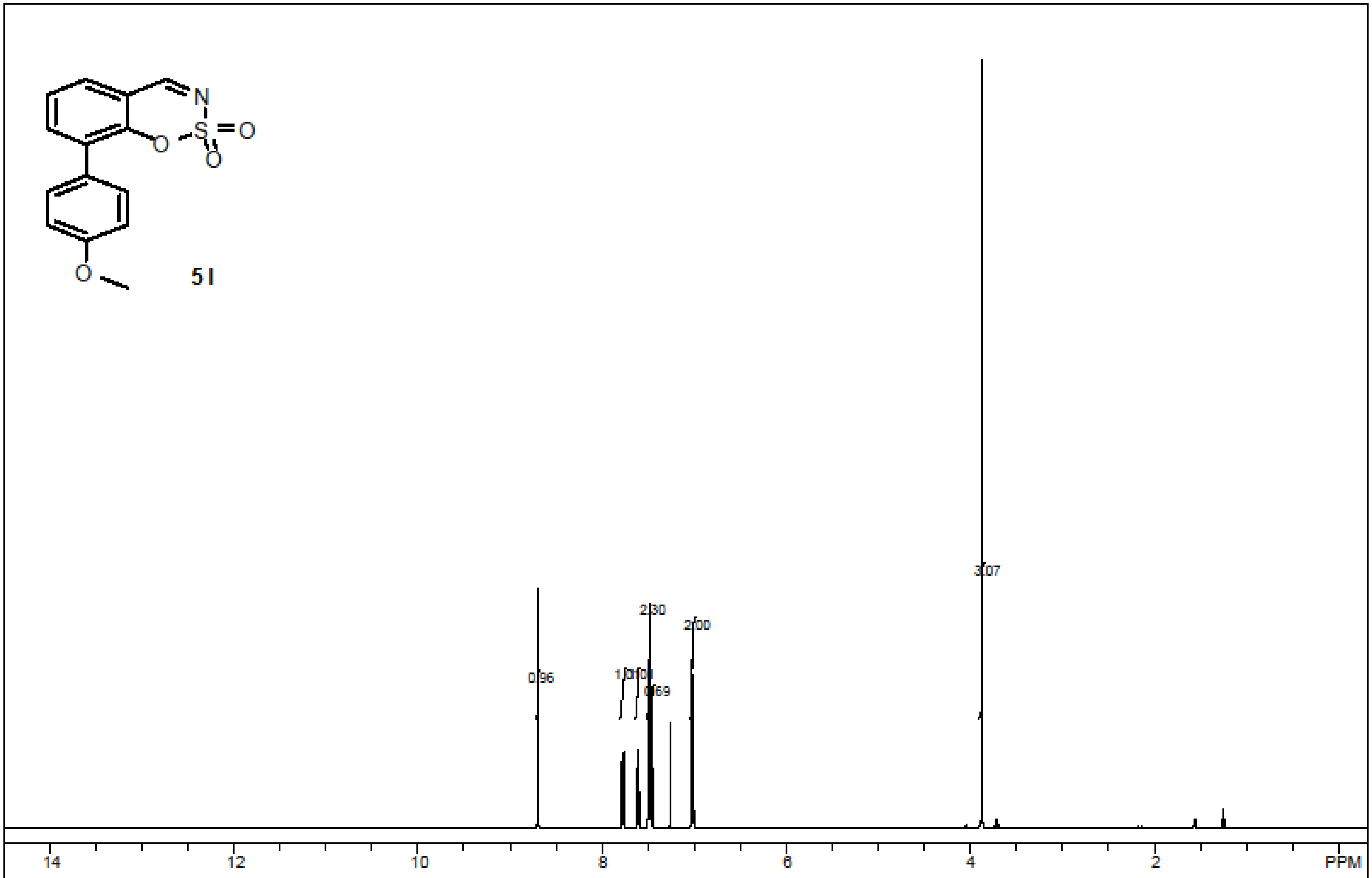

Avance, CDCl3, USER: nmrsu -- DATE: Wed Jan 20 14:30:44 2021

F1: 399.956	F2: 1.000	SW1: 7813		OF1: 2460.1		PTS1d: 65536	
EX: zg30		PW: 12.0 us	PD: 1.0 sec	NA: 16	LB: 0.0		Nuts - $pdata

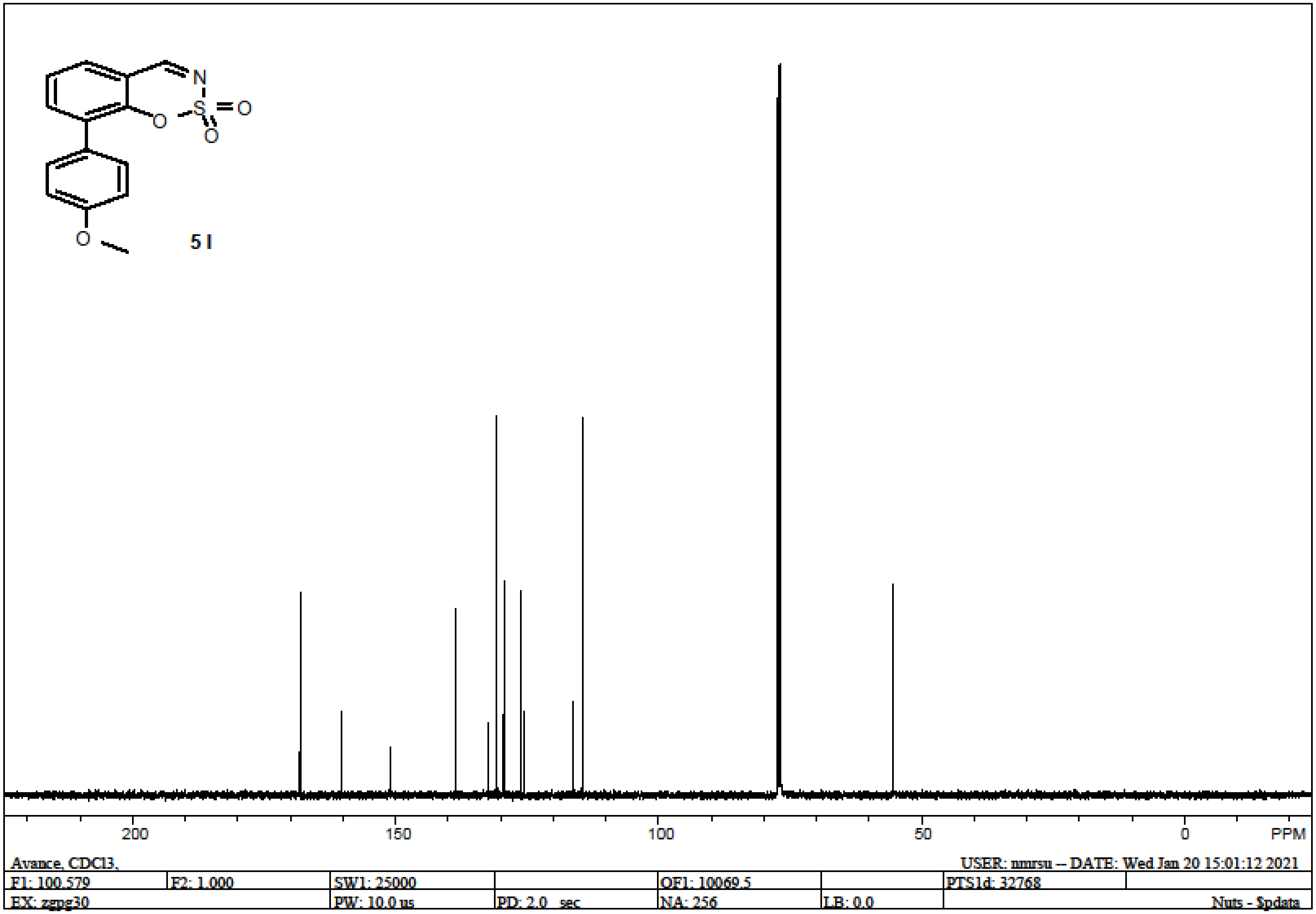
5 l
200
150
100
50
0
PPM
Avance, CDCl3,
USER: nmrsu -- DATE: Wed Jan 20 15:01:12 2021
F1: 100.579
F2: 1.000
SW1: 25000
OF1: 10069.5
PTS1d: 32768
EX: zgpg30
PW: 10.0 us
PD: 2.0 sec
NA: 256
LB: 0.0
Nuts - $pdata

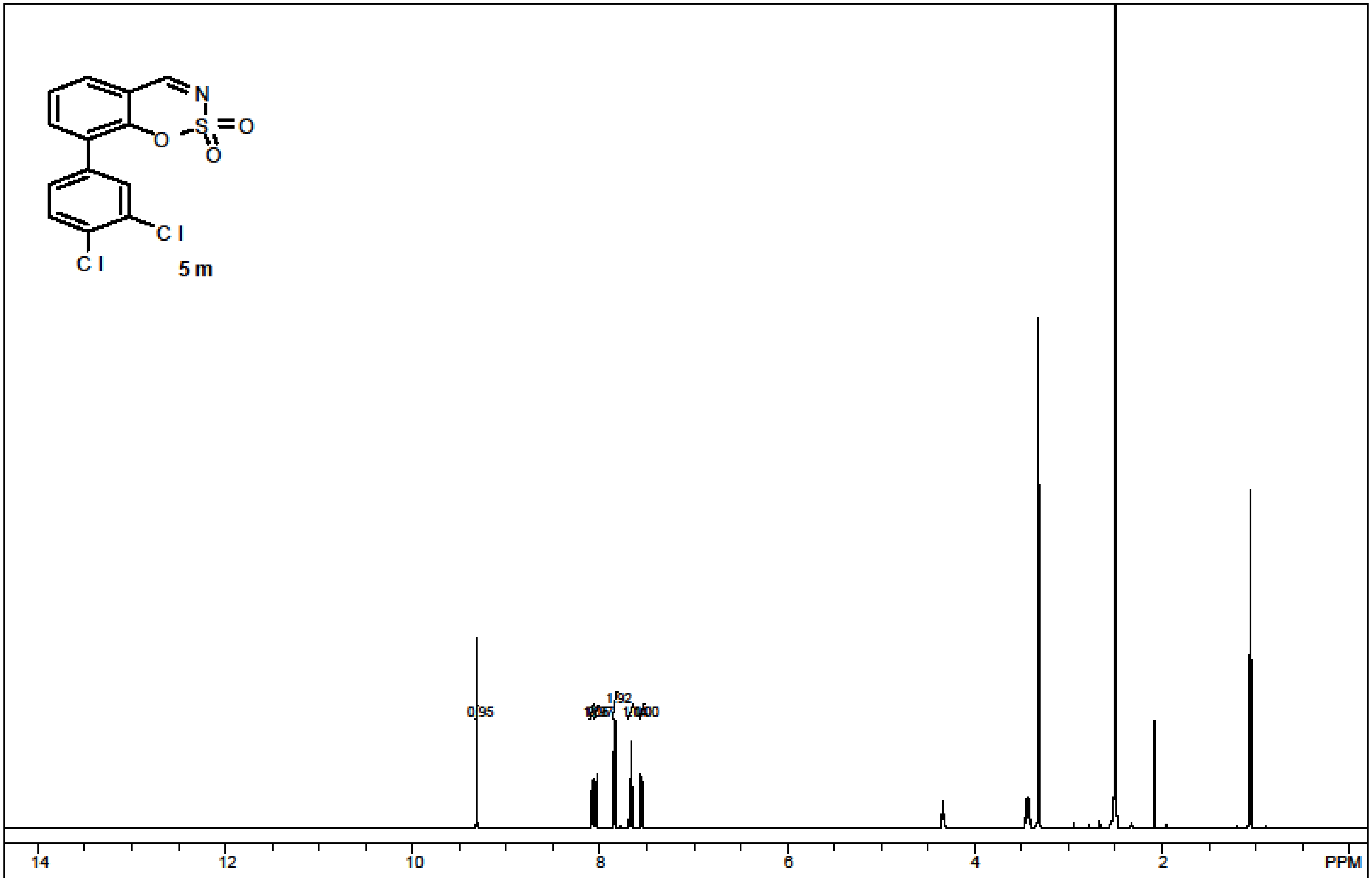

Avance, DMSO, USER: nmrsu -- DATE: Mon Jan 25 08:51:52 2021

F1: 399.956	F2: 1.000	SW1: 7813		OF1: 2466.5		PTS1d: 65536	
EX: zg30		PW: 12.0 us	PD: 1.0 sec	NA: 16	LB: 0.0		Nuts - $pdata

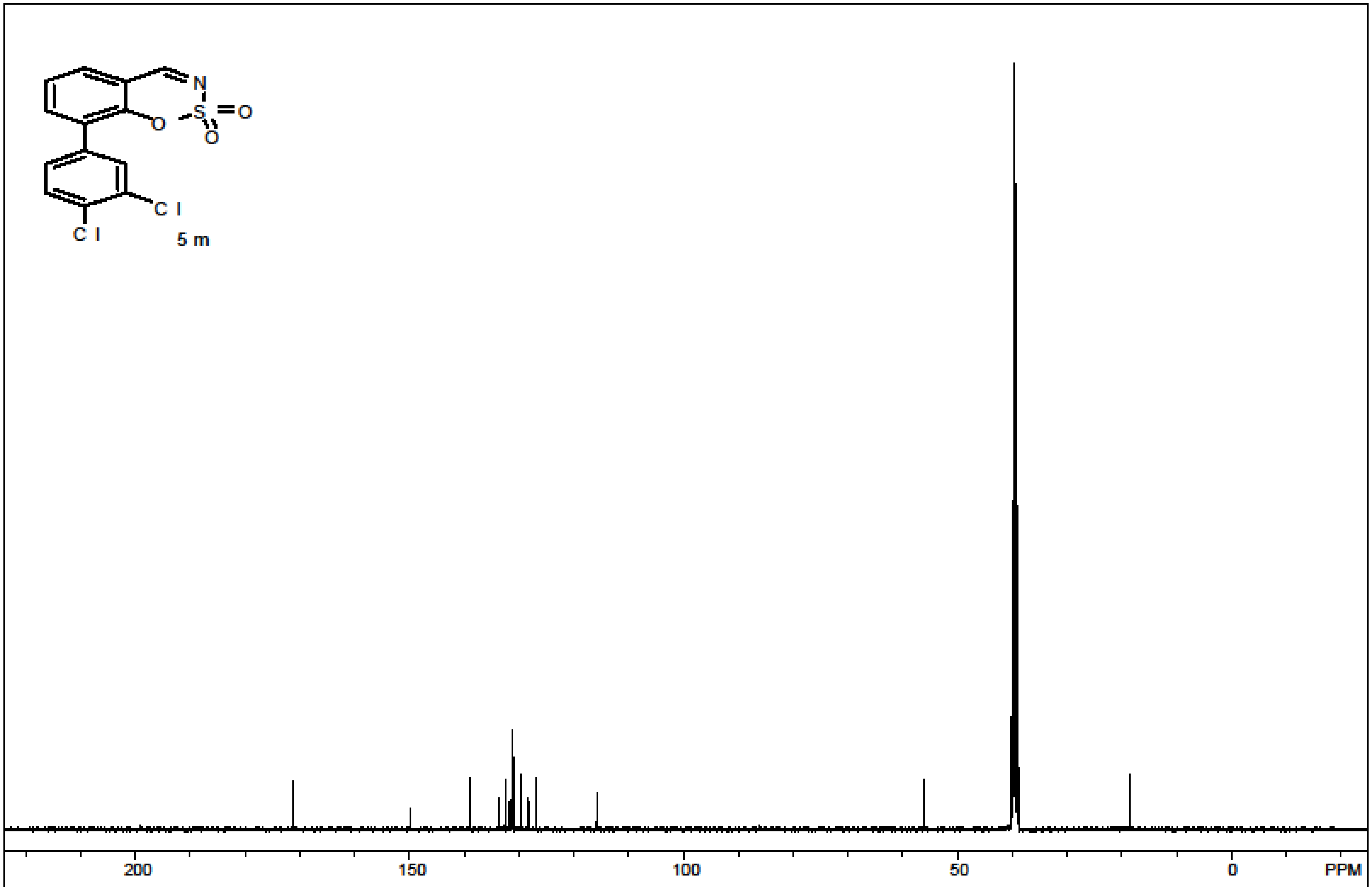

Avance, DMSO,

USER: nmrsu -- DATE: Tue Feb 23 12:36:17 2021

F1: 100.579	F2: 1.000	SW1: 25000		OF1: 10012.7		PTS1d: 32768	
EX: zgpg30		PW: 10.0 us	PD: 2.0 sec	NA: 256	LB: 0.0	Nuts - $pdata	
